# Supplementary material for: Cell fate in antiviral response arises in the crosstalk of IRF, NF-κB and JAK/STAT pathways
Source: Nat Commun. 2018 Feb 5;9:493. doi: 10.1038/s41467-017-02640-8 (PMC5799375; doi:10.1038/s41467-017-02640-8)
Supplement: Supplementary file 1 — Supplementary Information [file 41467_2017_2640_MOESM1_ESM.pdf]

Supplementary Information

"Cell fate in antiviral response arises in the crosstalk of IRF, NF-κB and JAK/STAT pathways"

by Czerkies *et al.*, *Nature Communications*, 2018

Supplementary Figures . . . 2
- Supplementary Figure 1 . . . 2
- Supplementary Figure 2 . . . 3
- Supplementary Figure 3 . . . 4
- Supplementary Figure 4 . . . 5
- Supplementary Figure 5 . . . 6
- Supplementary Figure 6 . . . 7
- Supplementary Figure 7 . . . 8
- Supplementary Figure 8 . . . 9
- Supplementary Figure 9 . . . 10
- Supplementary Figure 10 . . . 11

Supplementary Tables . . . 12
- Supplementary Table 1 . . . 12
- Supplementary Table 2 . . . 13
- Supplementary Table 3 . . . 14
- Supplementary Table 4 . . . 15
- Supplementary Table 5 . . . 16
- Supplementary Table 6 . . . 17
- Supplementary Table 7 . . . 18
- Supplementary Table 8 . . . 20

Supplementary Note – Computational model . . . 21

1 Computational model description . . . 21
- 1.1 System inputs . . . 21
- 1.2 Signal processing . . . 22
- 1.3 Gene regulation and transcription . . . 23
- 1.4 Numerical simulation protocols . . . 23
- 1.5 Model components and structure . . . 25
  - Table A. Molecular species . . . 25
  - Table B. Numbers of molecules per cell . . . 28
  - Table C. Denotational conventions . . . 29
  - Table D. Reactions and coefficients . . . 30

2 mRNA levels: RT-PCR results *vs.* model predictions . . . 43
- Figure A. MEF WT cells stimulated with poly(I:C) . . . 43
- Figure B. MEF WT cells stimulated with IFNβ . . . 44
- Figure C. MEF WT cells incubated with α-IFNAR & stimulated with poly(I:C) . . . 45
- Figure D. MEF WT cells stimulated with LPS . . . 46
- Figure E. MEF *RelA*$^{-/-}$ cells stimulated with poly(I:C) . . . 47
- Figure F. MEF *Stat1*$^{-/-}$ cells stimulated with poly(I:C) . . . 48

Supplementary References . . . 49

Supplementary Figures

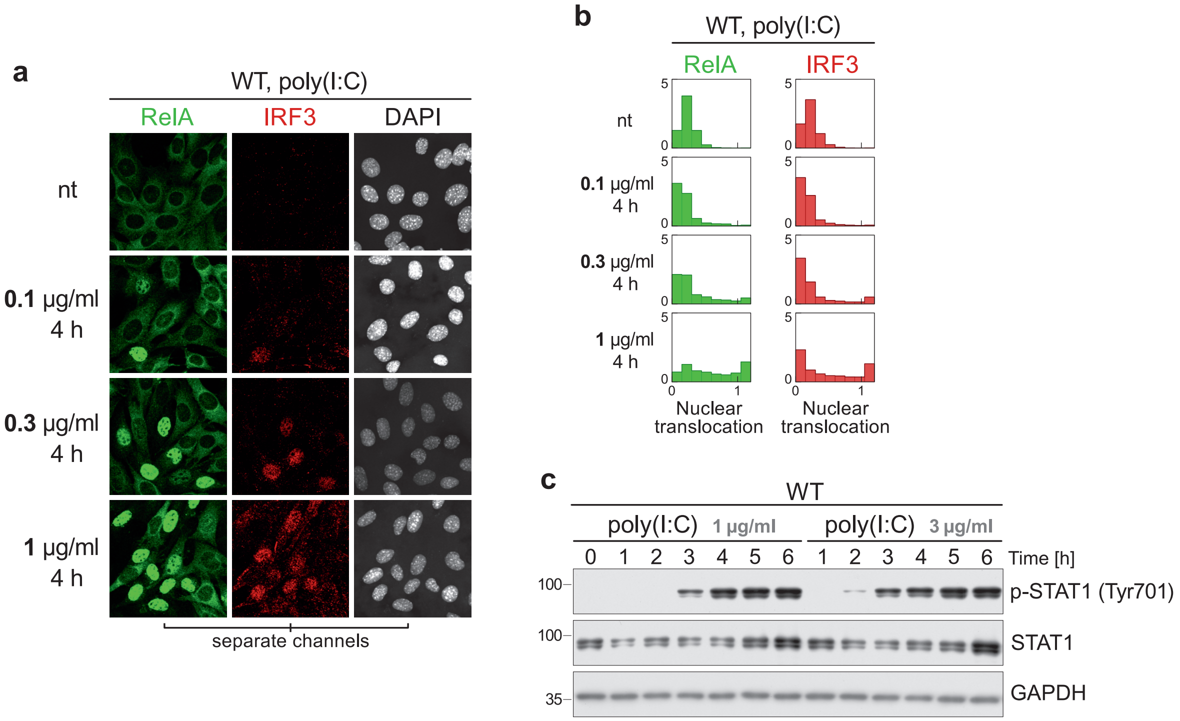

Supplementary Figure 1. RelA and IRF3 activation in MEF WT cells: (**a**) confocal images of immunofluorescence staining for RelA and IRF3 (scale bar: 50 µm) and (**b**) histograms ($n \geq 500$, from a representative experiment out of 2) from immunofluorescence analysis after 4 hr poly(I:C) 0.1, 0.3, and 1 µg/ml. In both panels, 'nt' denotes non-treated cells. Full immunostaining images are shown in Supplementary Data 9. (**c**) Western blot for STAT1 and p-STAT1 with reference GAPDH after poly(I:C) 1 µg/ml and 3 µg/ml in WT cells. A representative replicate (out of 2) is shown.

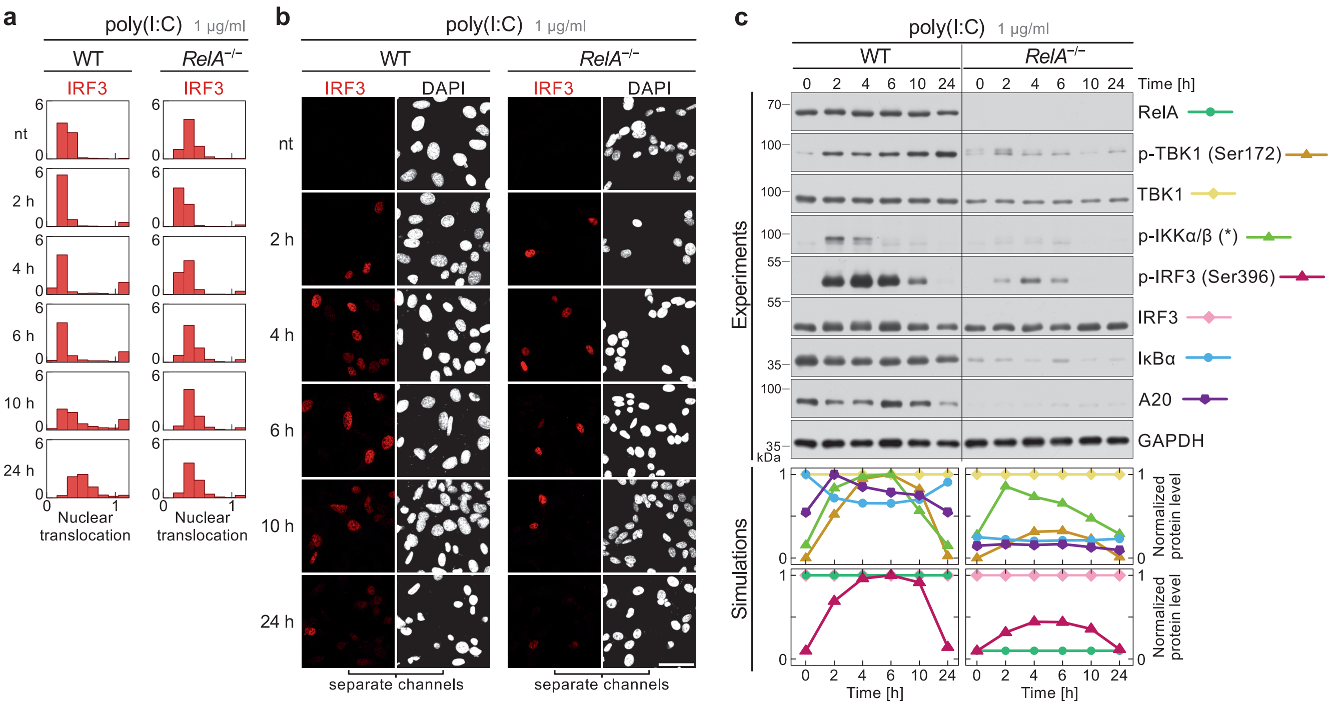

Supplementary Figure 2. RelA and IRF3 activation after poly(I:C) 1 µg/ml dose in MEF WT and *RelA*$^{-/-}$ cells: (**a**) histograms ($n \geq 300$, from a representative experiment out of 2), (**b**) confocal images from immunostaining (scale bar: 50 µm; full immunostaining images for *RelA*$^{-/-}$ cells are shown in Supplementary Data 10), (**c**) Western blots and model predictions: RelA, p-TBK1, p-IKKα/β, p-IRF3, IRF3, IκBα, A20, and reference GAPDH. A representative replicate out of 2 is shown. In (a) and (b), 'nt' denotes non-treated cells. In (c), asterisk (*) denotes IKK isoform-dependent phosphorylation sites: Ser176/180 in the case of IKKα and Ser177/181 in the case of IKKβ. Numerical trajectories result from averaging over 200 independent stochastic simulations. Colour key of numerical trajectories is located next to blot labels. To compare simulation trajectories with experimental data, numerical solutions are given in experimental time points only and then connected by line segments to guide the eye.

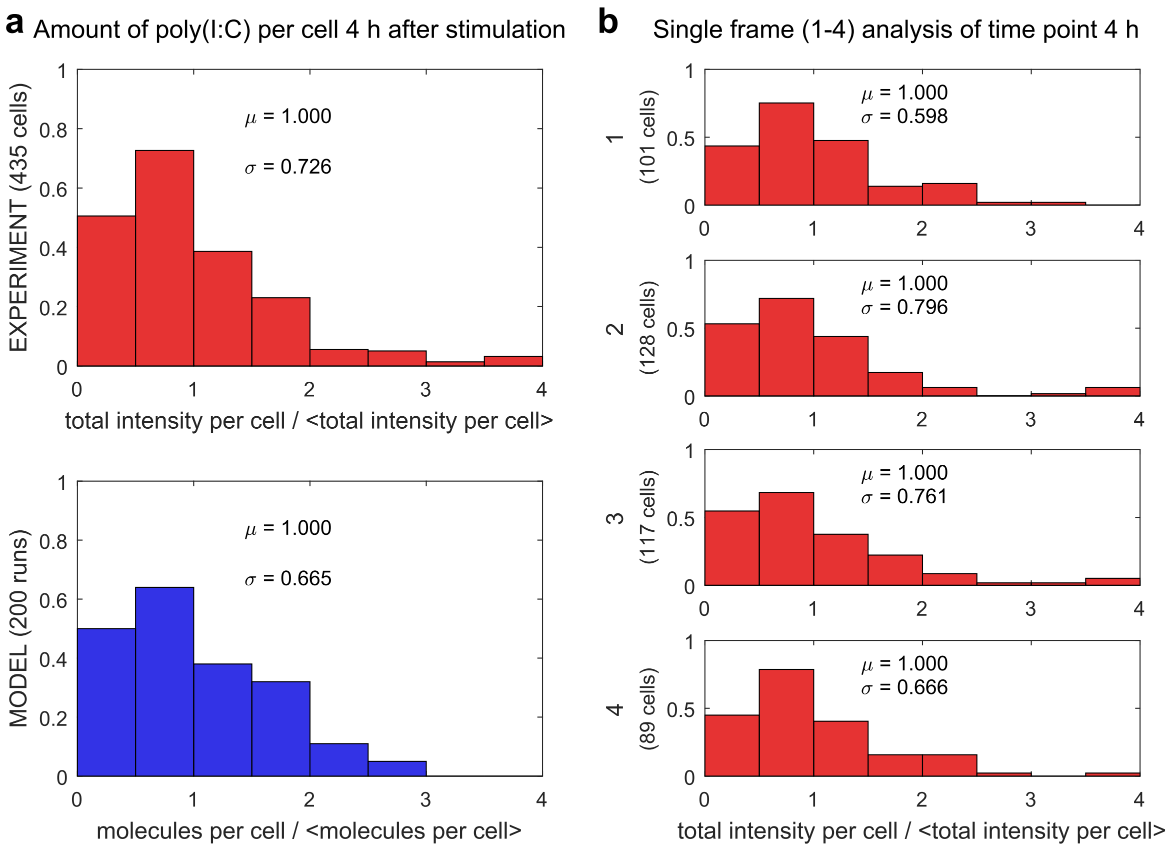

Supplementary Figure 3. Histograms of the amount of intracellular poly(I:C) 4 hr after stimulation: (**a**) experiments (top histogram) compared to model simulations (bottom histogram); (**b**) single-frame analysis of time point 4 hr from experiments summarized in (a).

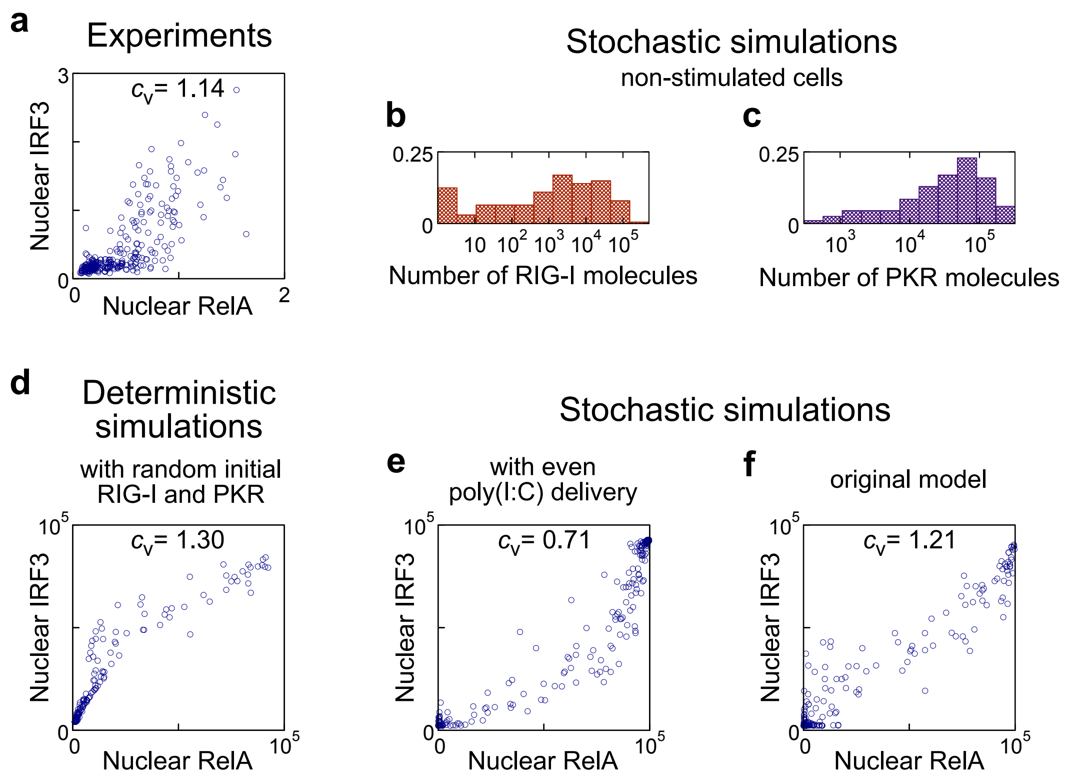

g

	Experiments	Deterministic simulations with random initial RIG-I and PKR	Stochastic simulations with even poly(I:C) delivery	Stochastic simulations original model
c_v	1.14	1.3	0.71	1.21
$\sigma_{RelA,IRF3}$	0.57	34461	54400	48205
σ_{RelA}	0.32	24573	40543	37896
σ_{IRF3}	0.46	24161	36271	29792
$\mu_{RelA,IRF3}$	0.61	26480	76377	39808
μ_{RelA}	0.43	14818	60161	29747
μ_{IRF3}	0.42	21946	47055	26455

Supplementary Figure 4. Variability in RelA and IRF3 activation in MEF WT in response to poly(I:C) stimulation – experiments compared to numerical simulations: (**a**) scatter plots of nuclear IRF3 and nuclear RelA 4 hr after stimulation in experiments; (**b**) distribution of RIG-I and (**c**) distribution of PKR in non-stimulated cells used to assign random initial RIG-I and PKR values in (**d**) numerical deterministic simulations with even poly(I:C) delivery; (**e**) stochastic simulations with even poly(I:C) delivery and (**f**) stochastic simulations of the original model. Table (**g**) shows coefficients of variation, cv, standard deviations, σ, and average values, μ, calculated from data shown in (a) and (d–f).

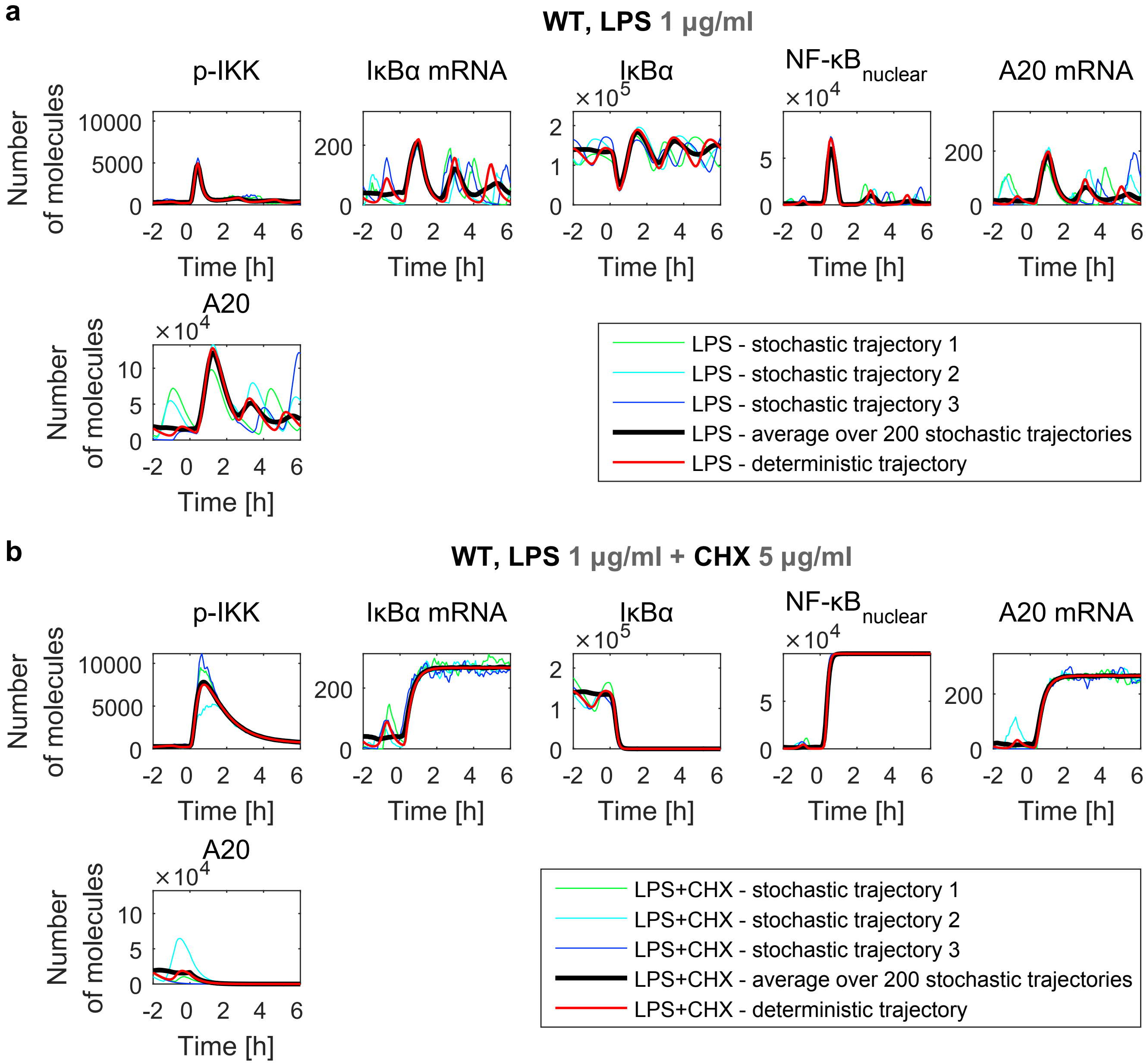

Supplementary Figure 5. Numerical simulations of WT cells (**a**) stimulated with LPS 1 µg/ml (since time = 0) and (**b**) stimulated with LPS 1 µg/ml and CHX (since time = 0).

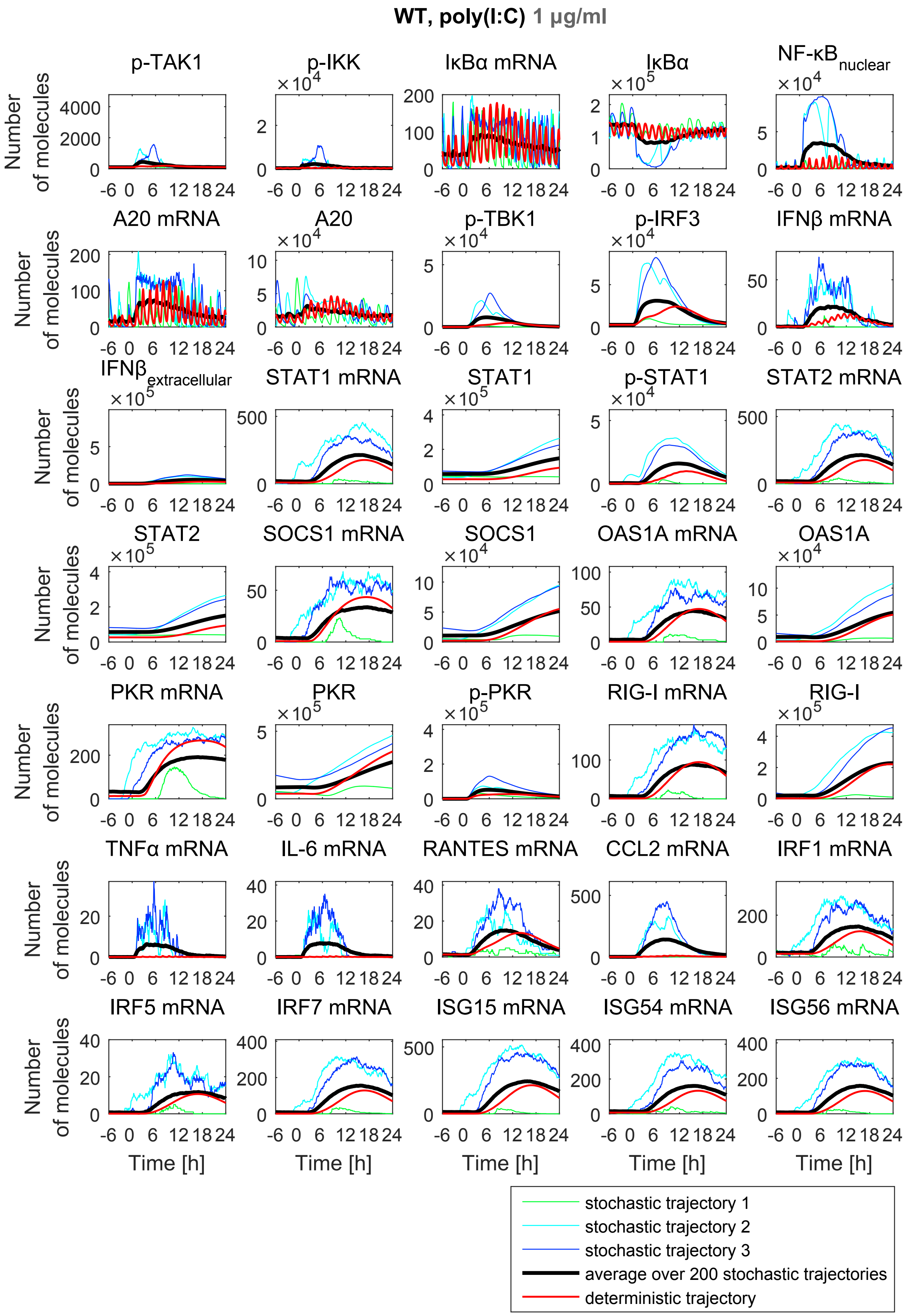

Supplementary Figure 6. Numerical simulations of MEF WT cells stimulated with poly(I:C) 1 µg/ml (since time = 0).

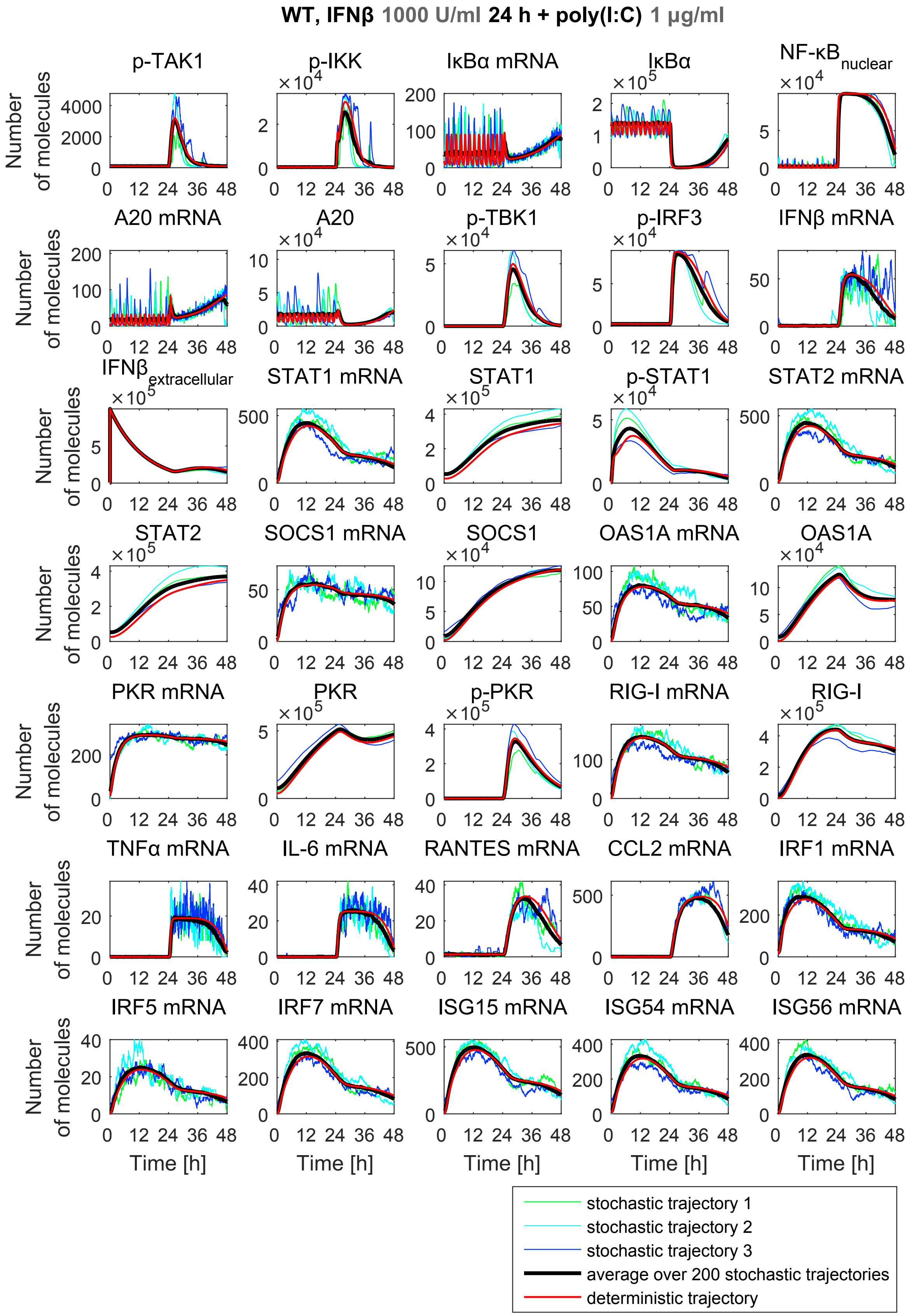

Supplementary Figure 7. Numerical simulations of MEF WT cells treated with IFNβ 1000 U/ml (from time = 0 till 24 hr) and stimulated with poly(I:C) 1 μg/ml (at 24 hr).

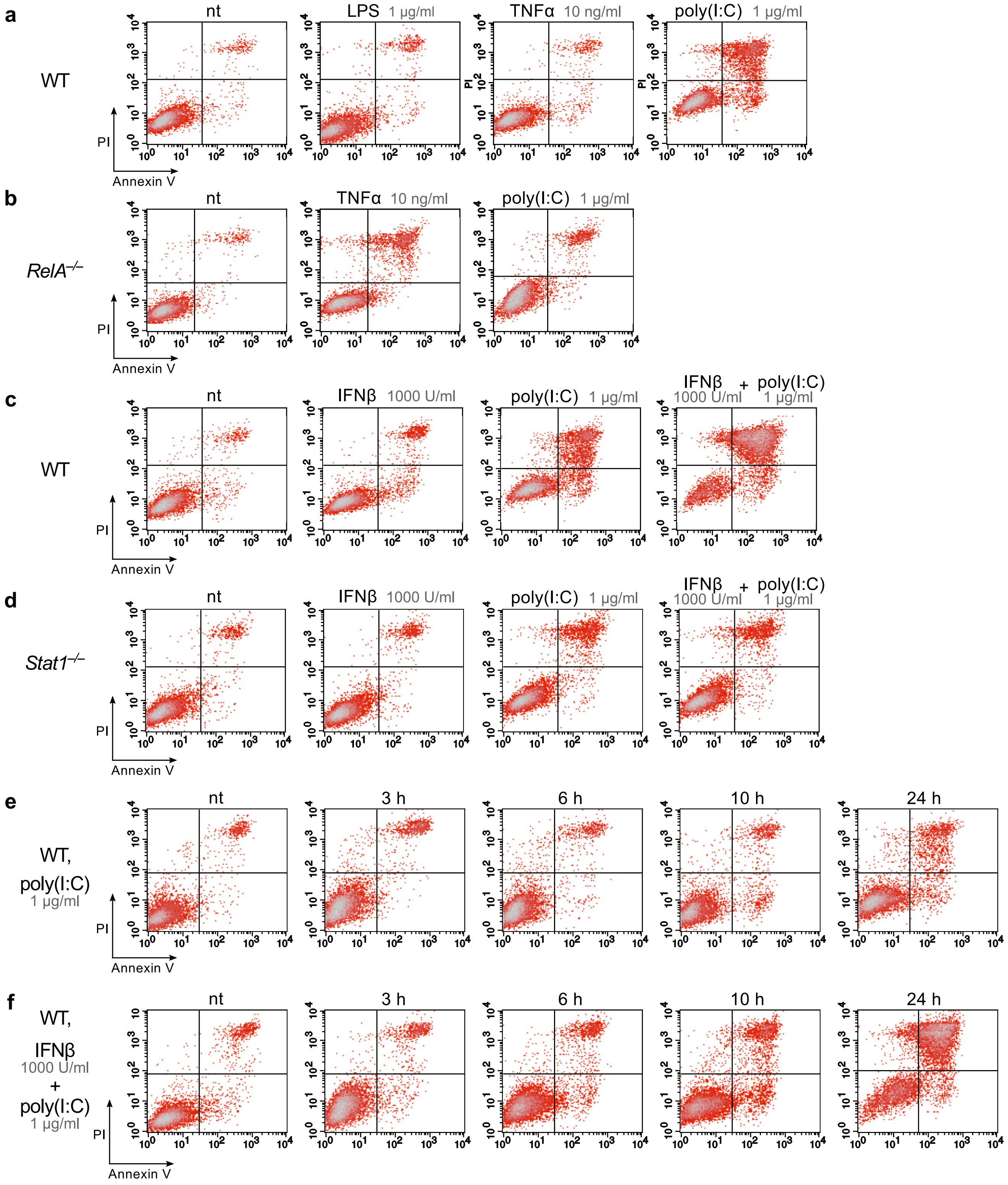

Supplementary Figure 8. Density plots of Annexin V/PI staining.

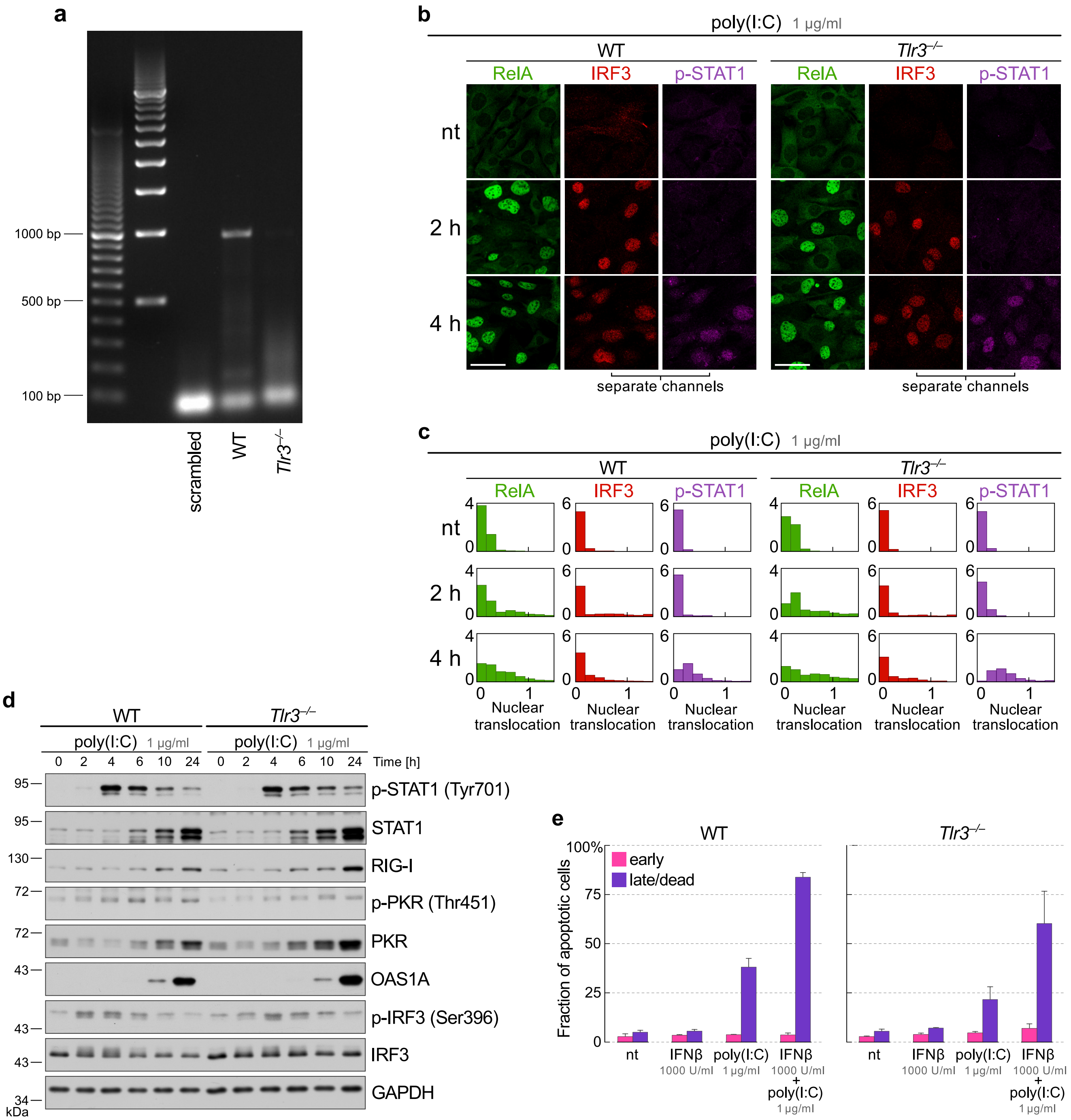

Supplementary Figure 9. Poly(I:C) delivered by lipid-based transfection elicits activation of RelA, IRF3 and STAT1 in *Tlr3*$^{-/-}$ MEFs. The figure compares results obtained on Tlr3–/– and WT MEFs purchased from OrientalBioService. (**a**) Genotyping of Tlr3 for WT and *Tlr3*$^{-/-}$ cell lines, by PCR assay, according to OrientalBioService protocol. Tlr3 band localizes at 1000 bp. (**b**) Confocal images of immunofluorescence staining for RelA, IRF3, and p-STAT1 (scale bar: 50 μm). See Supplementary Data 11 for the corresponding uncropped immunostaining images. (**c**) Histograms ($n \geq 500$, from a representative experiment out of 2) from immunofluorescence analysis after 0 (nt), 2, 4 hr of poly(I:C) stimulation. (**d**) Western blots for the proteins as indicated after poly(I:C) stimulation for WT and *Tlr3*$^{-/-}$ MEFs. Representative replicates out of 3 are shown. (**e**) Apoptotic cell fraction in response to poly(I:C) either with or without 24-hr IFNβ prestimulation for WT versus *Tlr3*$^{-/-}$ cells. Bars represent s.d., $n \geq 3$, data for all replicates are given in Supplementary Data 14.

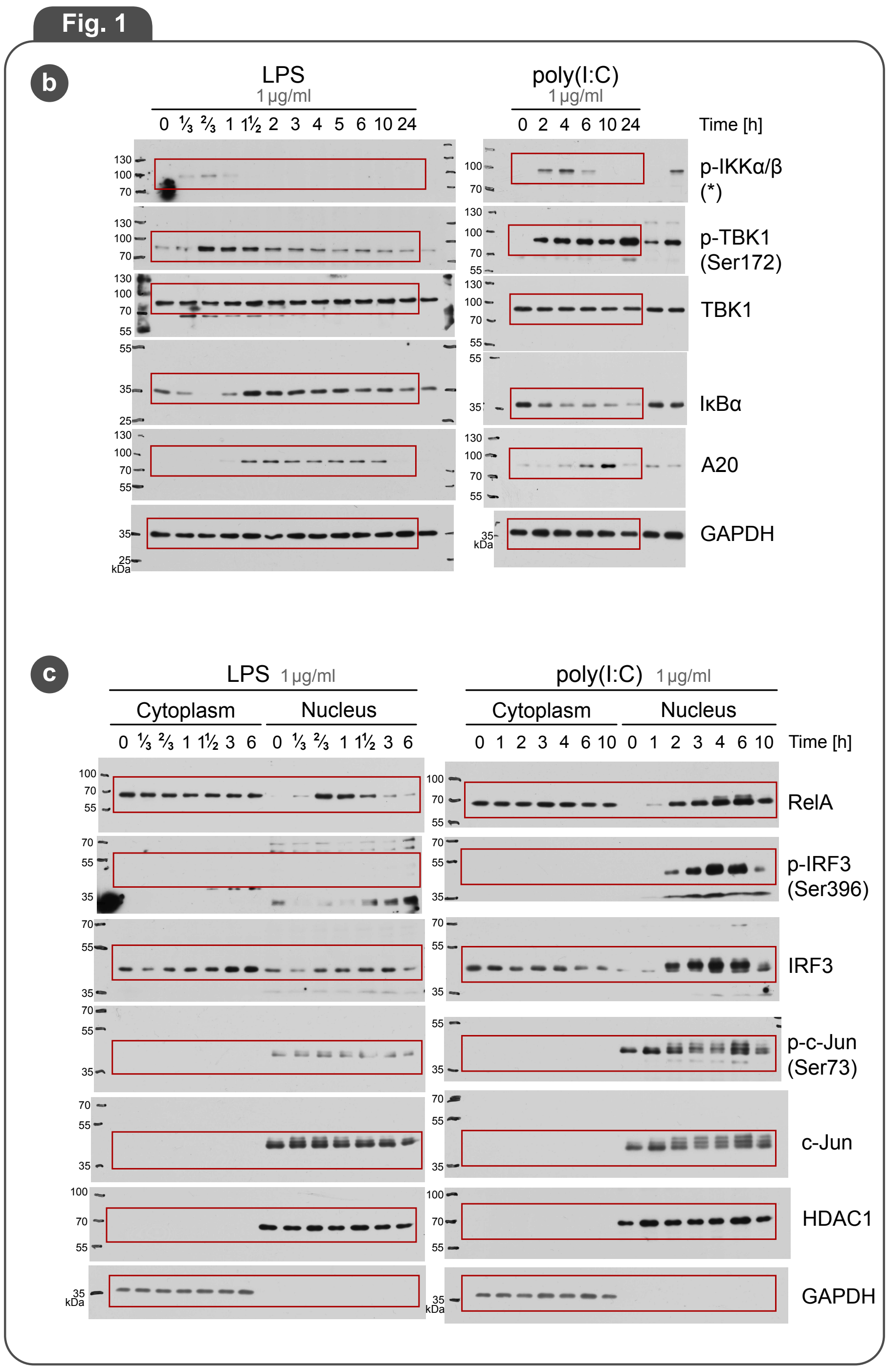

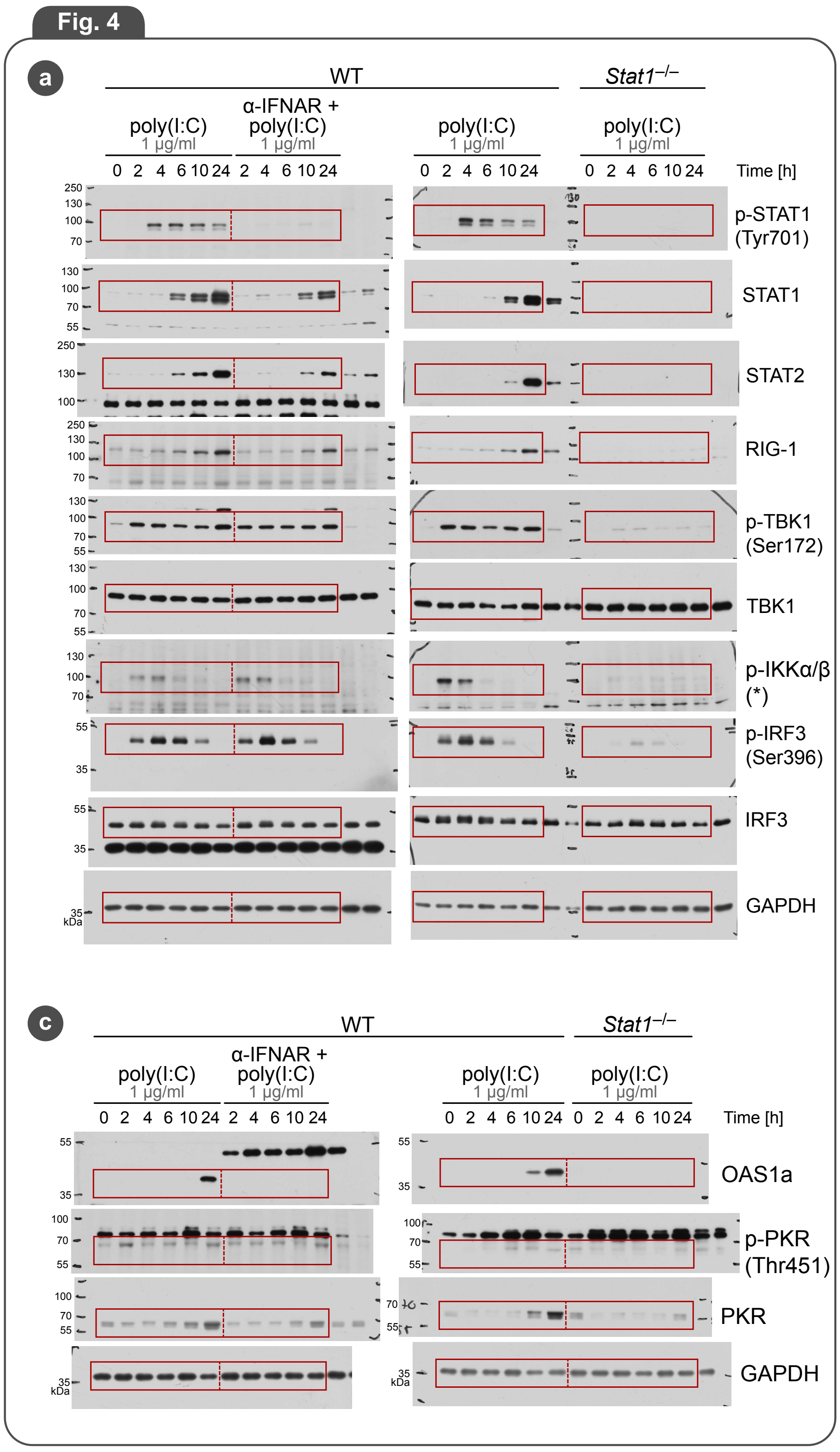

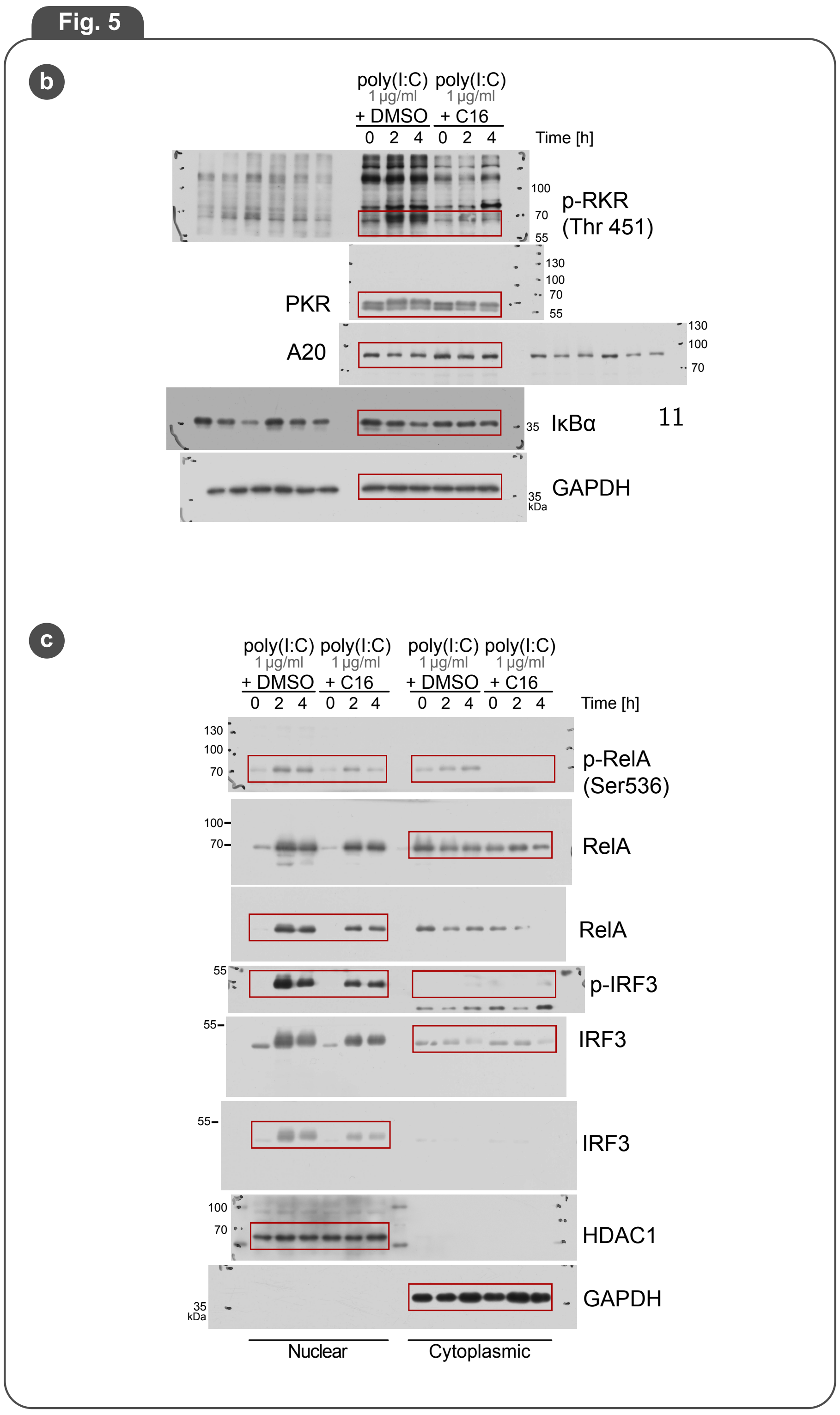

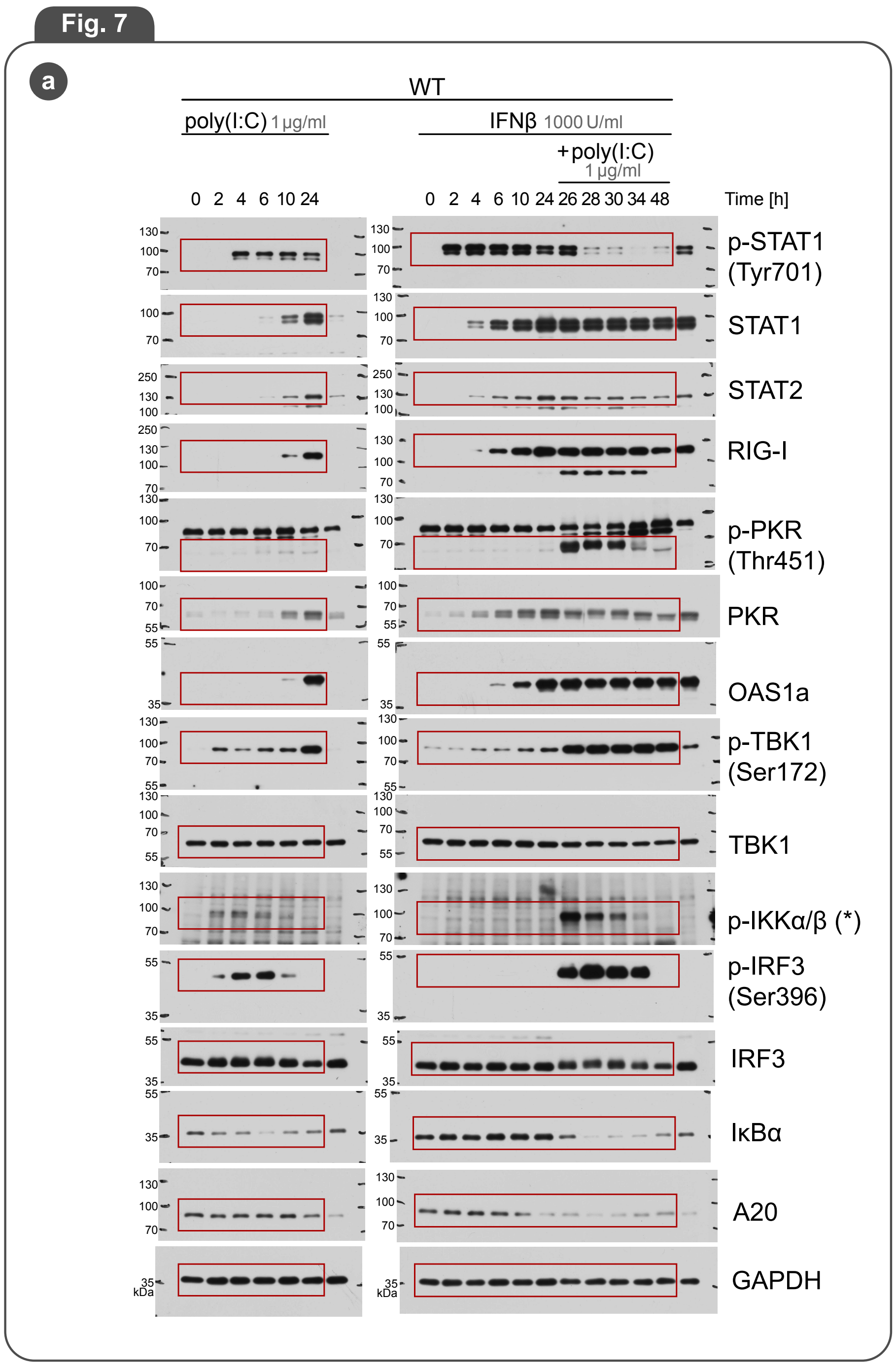

Supplementary Figure 10. Uncropped scans of blots shown in Figs 1b, 1c, 4a, 4c, 5b, 5c, 7a.

Supplementary Tables

Supplementary Table 1. Cell lines and stimulation protocols.

Cell line	Stimulations
MEF WT	poly(I:C) [0.1 μg/ml, 0.3 μg/ml, 1 μg/ml, 3 μg/ml],<br>poly(I:C) [1 μg/ml] + α-IFNAR,<br>poly(I:C) [1 μg/ml] + C16
MEF WT	LPS [1 μg/ml],<br>LPS [1 μg/ml] + CHX [5 μg/ml],<br>IFNβ [1000 U/ml]
MEF *RelA-GFP*	poly(I:C) [1 μg/ml, 3 μg/ml],<br>LPS [1 μg/ml]
MEF *RelA*$^{-/-}$	poly(I:C) [1 μg/ml, 3 μg/ml]
MEF *Stat1*$^{-/-}$	poly(I:C) [1 μg/ml],<br>IFNβ [1000 U/ml]
MEF WT<br>(Oriental BioService)	poly(I:C) [1 μg/ml],<br>IFNβ [1000 U/ml]
MEF *Tlr3*$^{-/-}$<br>(Oriental BioService)	poly(I:C) [1 μg/ml],<br>IFNβ [1000 U/ml]

Supplementary Table 2. Network components and experimental methods used to characterize their dynamics.

Protein	Gene	mRNA level	Protein level	Phosphorylation state	Subcellular localization
FEEDBACK PROTEINS/GENES					
A20	*Tnfaip3*	RT PCR + dPCR	WB		
c-Jun	*Jun*		WB	Ser73	WB
IKKα/β	*Chuk/Ikbkb*		WB	Ser176/180	
IFNβ1	*Ifnb1*	RT PCR + dPCR	ELISA		
IRF3	*Irf3*	RT PCR + dPCR	WB	Ser396	WB + IF
IκBα	*Nfkbia*	RT PCR + dPCR	WB		IF
OAS1A	*Oas1a*	RT PCR + dPCR	WB		
p65	*RelA*		WB		WB + IF
PKR	*Eif2ak2*	RT PCR + dPCR	WB	Thr451	
RIG-I	*Ddx58*	RT PCR + dPCR	WB		
SOCS1	*Socs1*	RT PCR + dPCR			
STAT1	*Stat1*	RT PCR + dPCR	WB	Tyr701	WB + IF
STAT2	*Stat2*	RT PCR + dPCR	WB		
TBK1	*Tbk1*		WB	Ser172	
REFERENCE PROTEINS/GENES					
GAPDH	*Gapdh*	RT PCR + dPCR	WB		WB
HDAC1	*Hdac1*	RT PCR	WB		WB
HGPRT	*Hprt1*	RT PCR			
READ-OUT GENES					
CCL2	*Ccl2*	RT PCR			
IL-6	*Il6*	RT PCR			
IRF1	*Irf1*	RT PCR			
IRF5	*Irf5*	RT PCR			
IRF7	*Irf7*	RT PCR + dPCR			
ISG15	*Isg15*	RT PCR			
ISG54	*Ifit2*	RT PCR			
ISG56	*Ifit1*	RT PCR			
RANTES	*Ccl5*	RT PCR			
TNFα	*Tnf*	RT PCR			

Supplementary Table 3. Gene expression assays.

Protein/gene	TaqMan gene expression assay #
A20/*Tnfaip3*	Mm00437121_m1
CCL2/*Ccl2*	Mm00441242_m1
GAPDH/*Gapdh*	Mm99999915_g1
IFNβ1/*Ifnb1*	Mm00439552_s1
IL-6/*Il6*	Mm00446190_m1
IRF1/*Irf1*	Mm01288580_m1
IRF3/*Irf3*	Mm00516784_m1
IRF5/*Irf5*	Mm00496477_m1
IRF7/*Irf7*	Mm00516788_m1
ISG15/*Isg15*	Mm01705338_s1
ISG54/*Ifit2*	Mm00492606_m1
ISG56/*Ifit1*	Mm00515153_m1
IκBα/*Nfkbia*	Mm00477798_m1
OAS1A/*Oas1a*	Mm00836412_m1
PKR/*Eif2ak2*	Mm01235643_m1
RANTES/*Ccl5*	Mm01302427_m1
RIG-I/*Ddx58*	Mm00554529_m1
SOCS1/*Socs1*	Mm00782550_s1
STAT1/*Stat1*	Mm00439531_m1
STAT2/*Stat2*	Mm00490880_m1
TNFα/*Tnf*	Mm99999068_m1

Supplementary Table 4. Primary antibodies.

Target protein (clone)	Manufacturer	Catalogue #	Application (concentration)
phospho-NF-κB p65 (Ser536) (93H1)	Cell Signaling Technology, Inc.	3033	WB (1:1000)
NF-κB p65 (D14E12) XP	Cell Signaling Technology, Inc.	8242	WB (1:1000), IF (1:1000)
phospho-IRF-3 Ser396 (4D4G)	Cell Signaling Technology, Inc.	4947	WB (1:1000)
IRF-3 (D83B9)	Cell Signaling Technology, Inc.	4302	WB (1:1000)
phospho-IKKα/β Ser176/180 (16A6)	Cell Signaling Technology, Inc.	2697	WB (1:1000)
phospho-STAT1 Tyr701 (58D6)	Cell Signaling Technology, Inc.	9167	WB (1:1000), IF (1:200)
RIG-I (D14G6)	Cell Signaling Technology, Inc.	3743	WB (1:1000)
A20/TNFAIP3 (D13H3)	Cell Signaling Technology, Inc.	5630	WB (1:1000)
phospho-TBK1/NAK Ser172 (D52C2) XP	Cell Signaling Technology, Inc.	5483	WB (1:1000)
TBK1/NAK (D1B4)	Cell Signaling Technology, Inc.	3504	WB (1:1000)
phospho-c-Jun Ser73 (D47G9) XP	Cell Signaling Technology, Inc.	3270	WB (1:1000)
c-Jun (60A8)	Cell Signaling Technology, Inc.	9165	WB (1:1000)
IκBα (L35A5)	Cell Signaling Technology, Inc.	4814	WB (1:1000), IF (1:500)
IRF-3 (D-3)	Santa Cruz Biotechnology	sc-376455	IF (1:1000)
Stat1 p84/p91 (E-23)	Santa Cruz Biotechnology	sc-346	WB (1:1000)
Stat2 (L-20)	Santa Cruz Biotechnology	sc-950	WB (1:1000)
phospho-PKR Thr451	Santa Cruz Biotechnology	sc-101784	WB (1:500)
PKR (B-10)	Santa Cruz Biotechnology	sc-6282	WB (1:500)
OAS1a (C-8)	Santa Cruz Biotechnology	sc-365357	WB (1:500)
HDAC1 (H-11)	Santa Cruz Biotechnology	sc-8410	WB (1:1000)
GAPDH (6C5)	EMD Millipore	MAB374	WB (1:20,000)

Supplementary Table 5. Secondary antibodies.

Target protein (clone)	Manufacturer	Catalogue #	Application (concentration)
Donkey anti-rabbit IgG (H+L), Alexa Fluor® 488 conjugate	Thermo Fisher Scientific	A-21206	IF (1:500)
Donkey anti-mouse IgG (H+L), Alexa Fluor® 555 conjugate	Thermo Fisher Scientific	A-31570	IF (1:500)
Goat anti-rabbit immunoglobulins/HRP	Dako	P 0448	WB (1:10,000)
Goat anti-mouse immunoglobulins/HRP	Dako	P 0447	WB (1:10,000)

Supplementary Table 6. Reagents, kits, and assays (other than that mentioned in Suppl. Tables 3, 4, 5).

Reagent	Manufacturer	Catalogue #
4′,6-Diamidino-2-phenylindole dihydrochloride (DAPI)	Sigma-Aldrich	D8417
Bio-Rad Protein Assay Dye Reagent	Bio-Rad	500-0006
bisBenzimide H 33342 trihydrochloride (Hoechst 33342)	Sigma-Aldrich	B2261
Bovine Serum Albumin	Sigma-Aldrich	A7906
Clarity™ Western ECL Blotting Substrate	Bio-Rad	1705061
cOmplete™, Mini, EDTA-free Protease Inhibitor Cocktail Tablets	Sigma-Aldrich	04693159001
Cycloheximide	Roth	8682.3
Dimethyl sulfoxide (DMSO)	Sigma-Aldrich	D8418
DL-Dithiothreitol (DTT)	Sigma-Aldrich	D9163
DMEM, high glucose	ThermoFischer Scientific	41965
Fetal Bovine Serum, qualified, E.U.-approved, South America origin	ThermoFischer Scientific	10270
Formaldehyde solution	Sigma-Aldrich	2525249
IGEPAL® CA-630	Sigma-Aldrich	I8896
Imidazolo-oxindole PKR inhibitor C16	Sigma-Aldrich	I9785
Interferon β from mouse	Sigma-Aldrich	I9032
Lipofectamine® LTX with Plus™ Reagent	ThermoFischer Scientific	15338-100
Lipopolysaccharide from *Escherichia coli* 0111:B4 (LPS)	Sigma-Aldrich	L3024
MEM Non-essential Amino Acid Solution	Sigma-Aldrich	M7145
Mowiol® 4-88	Sigma-Aldrich	81381
PageRuler™ Plus Prestained Protein Ladder, 10-250 kDa	ThermoFischer Scientific	26620
PBS, pH 7.4	ThermoFischer Scientific	10010-015
Penicillin-Streptomycin	Sigma-Aldrich	P0781
Polyinosinic–polycytidylic acid potassium salt (poly(I:C))	Sigma-Aldrich	P9582
Purified NA/LE Mouse anti-Mouse IFN-α/β Receptor 1	BD	561183
QuantStudio™ 3D Digital PCR Master Mix v2	ThermoFischer Scientific	A26358
Sodium fluoride	Sigma-Aldrich	S6776
Sodium orthovanadate	Sigma-Aldrich	450243
Sodium pyruvate	Sigma-Aldrich	S8636
TaqMan® Gene Expression Master Mix	ThermoFischer Scientific	4369016
Triton™ X-100	Sigma-Aldrich	T9284
Tumor Necrosis Factor-α from mouse	Sigma-Aldrich	T7539
TWEEN® 20	Sigma-Aldrich	P7949
Vectashield HardSet Antifade Mounting Medium with DAPI	Vector Laboratories	H-1500
**Kits**		
FITC Annexin V Apoptosis Detection Kit II	BD	556570
High-Capacity cDNA Reverse Transcription Kit	ThermoFischer Scientific	4368813
PureLink® RNA Mini Kit	ThermoFischer Scientific	12183018A
VeriKine Mouse Interferon Beta ELISA Kit	PBL Assay Science	42400-1

Supplementary Table 7. Juxtaposition of digital PCR versus real-time PCR quantifications.

Protein	Experiment		dPCR: $\log_2(\mathrm{mRNA}_n)$	dPCR & RT PCR: $\log_2(\mathrm{mRNA}_n) + \Delta C_T$	dPCR: $\log_2(GAPDH_{\mathrm{mRNA}})$
IκBα	WT, LPS, **D2**, 1 h		8.11	10.27	10.71
	WT, poly(I:C) 1 μg/ml, **A7**, 4 h		7.5	10.26	10.02
	WT, poly(I:C) 1 μg/ml, **A3**, 10 h		5.81	12.00	11.03
	WT, CHX + LPS	1 h	10.87	9.98	10.92
		2 h	10.72	9.93	11.33
				Average: 10.49<br>SEM: 0.38	
A20	WT, poly(I:C) 1 μg/ml, **A8**, 4 h		7.87	11.22	-
	WT, poly(I:C) 3 μg/ml, **A1**, 4 h		7.52	12.53	11.06
	WT, LPS, **D1**, 1h		8.67	11.79	10.8
				Average: 11.85<br>SEM: 0.38	
IRF3	WT, poly(I:C) 1 μg/ml, **A8**, 0 h		1.94	11.15	10.77
	WT, poly(I:C) 1 μg/ml, **A8**, 6 h		3.42	11.84	10.94
	WT, poly(I:C) 1 μg/ml, **A8**, 10h		2.59	11.29	10.09
				Average: 11.42<br>SEM: 0.21	
IRF7	WT, poly(I:C) 1 μg/ml, **A3**, 0 h		0.14	9.88	10.26
	WT, poly(I:C) 1 μg/ml, **A3**, 10 h		7.46	11.07	10.55
	RelA$^{-/-}$ control in **A3** (poly(I:C) 1 μg/ml), 10 h		2.54	11.17	11.56
				Average: 10.71<br>SEM: 0.41	
IFNβ1	WT, poly(I:C) 1 μg/ml, **A7**, 0 h		-0.74	9.16	-
	WT, poly(I:C) 1 μg/ml, **A7**, 4 h		3.95	9.53	10.02
	WT, poly(I:C) 3 μg/ml, **A2**, 4 h		4.94	9.98	-
				Average: 9.56<br>SEM: 0.24	
STAT1	WT, poly(I:C) 1 μg/ml, **A5**, 0 h		2.93	12.28	11.16
	WT, poly(I:C) 1 μg/ml, **A5**, 6 h		6.61	12.16	11.29
	WT, poly(I:C) 1 μg/ml, **A5**, 10 h		8.17	12.49	11.03
	WT, poly(I:C) 1 μg/ml, **A9**, 0 h		3.35	11.45	10.4
	WT, poly(I:C) 1 μg/ml, **A9**, 6 h		8.14	11.4	10.31
	WT, poly(I:C) 1 μg/ml, **A9**, 10 h		9.13	11.85	10.56
				Average: 11.94<br>SEM: 0.18	

continued				
STAT2	WT, IFNβ 1000 U/ml, **B3**, 2 h	7.39	12.23	-
	WT, IFNβ 1000 U/ml, **B3**, 4 h	7.99	12.15	-
	WT, IFNβ 1000 U/ml, **B3**, 10 h	8.58	12.19	-
			Average: 12.19<br>SEM: 0.02	
SOCS1	WT, IFNβ 1000 U/ml, **B2**, 0 h	1.72	9.68	-
	WT, IFNβ 1000 U/ml, **B2**, 6 h	5.29	10.27	-
	WT, IFNβ 1000 U/ml, **B2**, 10 h	4.74	10.8	-
			Average: 10.25<br>SEM: 0.32	
OAS1A	WT, poly(I:C) 1 μg/ml, **A5**, 0 h	0.26	13.21	11.16
	WT, poly(I:C) 1 μg/ml, **A5**, 6 h	4.26	13.33	11.29
	WT, poly(I:C) 1 μg/ml, **A5**, 10 h	6.05	13.61	11.03
			Average: 13.38<br>SEM: 0.12	
PKR	WT, poly(I:C) 1 μg/ml, **A9**, 0 h	4.72	13.32	10.4
	WT, poly(I:C) 1 μg/ml, **A9**, 6 h	7.58	12.96	10.31
	WT, poly(I:C) 1 μg/ml, **A9**, 10 h	7.95	13.58	10.56
			Average: 13.29<br>SEM: 0.18	
RIG-I	WT, poly(I:C) 1 μg/ml, **A3**, 0 h	2.10	10.31	10.26
	WT, poly(I:C) 1 μg/ml, **A3**, 10 h	6.71	11.13	10.55
	RelA$^{-/-}$ control in **A3** (poly(I:C) 1 μg/ml), 10 h	5.69	11.83	11.56
			Average: 11.09<br>SEM: 0.44	
All above proteins			Average: 11.47<br>SD: 1.16	
GAPDH	All above experiments			Average: 10.76<br>SEM: 0.1

Supplementary Table 8. Secreted IFNβ levels measured by ELISA in WT cells after different stimuli or in perturbed cells after poly(I:C). The table contains source data for Fig. 3b with all replicates in pg/ml. In thin blue frames are technical replicates from measurements obtained using a 72-well ELISA plate from the same experiment.

	**2 hr**	**4 hr**	**6 hr**	**10 hr**	**24 hr**
**WT, LTX**				1.85 1.66	2.60 1.60
**WT, TNFα** 10 ng/ml	3.56 3.05	3.30 3.96	3.05 3.17	6.15 3.33<br>9.96 9.56 9.89	3.84 3.44<br>9.86 9.82 9.51
**WT, LPS** 1 μg/ml	5.28 2.63 3.75	7.76 2.61 2.96	2.44 2.63 2.26	2.17 2.63 2.40<br>9.35 10.01 9.47	2.19 2.40 1.98<br>9.73 9.45 9.41
**WT, poly(I:C)** 0.1 μg/ml		11.88 9.41 8.08<br>12.48 10.81 11.22	21.01 22.29 27.83<br>30.19 25.11 22.40	83.33 84.22 95.34<br>104.64 93.22 78.36	1041.45 1025.60 912.05<br>960.42 919.03 710.00
**WT, poly(I:C)** 0.3 μg/ml		17.30 12.46 7.64	46.38 24.22 18.90	101.69 99.17 190.31	1107.25 1044.64 719.02
**WT, poly(I:C)** 1 μg/ml	2.49 1.26 1.79	5.89 7.38 4.49<br>19.15 29.97 19.54<br>23.86 38.33 29.58	30.20 29.74 33.46<br>141.65 67.81 119.33<br>167.65 101.46 181.69	108.36 123.71 81.40<br>609.09 510.15 539.61<br>748.17 606.59 1052.79	761.21 699.30 866.83<br>1267.40 1155.35 1135.40<br>1279.66 1296.70 1311.00
**WT, poly(I:C)** 3 μg/ml	1.82 3.28 2.24	9.91 8.50 6.61<br>32.18 20.37 32.89	52.81 55.66 71.43<br>152.56 202.88 182.71	155.24 165.52<br>714.07 777.05 722.22	568.59 634.76<br>1619.18 1671.74 1412.32
**WT, α-IFNAR AB + poly(I:C)** 1 μg/ml	2.93 3.63 2.14	3.79 10.87 3.65	29.69 45.47	64.11 55.47	178.66 87.58 88.93
***RelA$^{-/-}$*, poly(I:C)** 1 μg/ml		4.11 2.67 2.79	6.00 7.52 9.67	20.56 24.42 23.64<br>48.67 46.79 50.21	188.10 175.24 178.56<br>334.74 474.80 409.52
***RelA$^{-/-}$*, poly(I:C)** 3 μg/ml		2.54 3.17 5.69	17.43 11.44 18.58	34.58 37.13 41.12	279.36 444.25 376.95
***Stat1$^{-/-}$*, poly(I:C)** 1 μg/ml				70.82 94.24 111.24	59.80 52.61 59.74
***Stat1$^{-/-}$*, poly(I:C)** 3 μg/ml					385.93 309.06 233.93

Supplementary Note – Computational model

1 Computational model description

We used the model specification language of BioNetGen[1] (BNGL) to define types of molecules and to specify biochemical reactions. Overall, in the model there are 60 types of molecules (giving rise to 147 molecular species) and 291 reactions; nearly all reactions follow the mass action kinetics. The list of molecular species, assumed numbers of molecules per cell and list of reactions with rate coefficients are provided in Table A, Table B, and Table D, respectively; Table C explains denotational conventions used in Table D. As discussed in the main text, the model parameters cannot be uniquely identified based on available experimental data, i.e., changes of a given parameter in most cases can be compensated by changes of the remaining parameters[2]. Below, we briefly discuss the structure of the model and single-cell simulation protocols. Model execution instructions are provided in a separate ReadMe file featuring machine-readable model implementations.

1.1 System inputs

LPS stimulation and IFNβ stimulation. We assume that 1 μg/ml of LPS corresponds to 10^6 mlcs/cell and 1000 U/ml IFNβ corresponds to 10^6 mlcs/cell. These numbers are not very important for the downstream signaling in the sense that the strength of the signal also depends on the number of receptors and the receptor–ligand binding/unbinding constants.

Poly(I:C) stimulation. In experiments, poly(I:C) is administered within liposomes. Time-lapse images (see Supplementary Video 1) indicated that the expected number of liposomes per cell is of order of 10, and that the liposomes have different sizes and contain different amounts of poly(I:C). To account for the heterogeneity in the amounts of molecules of poly(I:C) entering each cell, we simulated poly(I:C) delivery in the following way: We assume that 100 liposomes are distributed between 10 cells so that each liposome has equal probability to enter any given cell. This is, we assume that the simulated cell internalizes liposomes with the rate $V_{liposome}$, while the total liposomes internalization rate (by the simulated cell and other 9 non-simulated cells) is equal $10 \times V_{liposome}$. After internalization, each liposome secretes poly(I:C) with the rate S_{polyIC} and is degraded with the rate $L_{liposome\_cyt}$. As a result, in stochastic simulations the numbers of poly(I:C) molecules secreted by single liposomes follow the geometric distribution. The parameters are adjusted so that for 1 μg/ml dose the average number of poly(I:C) molecules entering a cell equals 10^5. This is about 1% of total poly(I:C) molecules/cell for that dose, but we expect that majority of poly(I:C) molecules do not reside within liposomes and cannot enter cells.

In Supplementary Fig. 3 we show that the experimentally measured distribution of poly(I:C) per cell closely matches the distribution obtained in simulations as described above. In Supplementary Fig. 4 we show that heterogeneity of poly(I:C) uptake by cells contributes to heterogeneity of their responses (panels e *vs.* f). The uneven uptake of poly(I:C) causes that the fraction of cells exhibiting significant nuclear translocation is smaller (see panel g) and as a result coefficient of variation ($c_{\mathrm{v}} = \sigma/\mu$) is higher.

1.2 Signal processing

LPS stimulation. LPS stimulation leads to NF-κB activation. This part of the model follows closely our previous model of the NF-κB module[3,4]. LPS binds TLR4 receptor; then, LPS-bound TLR4 receptor phosphorylates (activates) kinase TAK1 which subsequently phosphorylates (activates) kinase IKK. IKK exists in one of four different states: non-active IKK_n, active IKK_a, inactive IKK_i and second inactive IKK_{ii}. TAK1 may phosphorylate only IKK_n to IKK_a, then IKK_a is inactivated to the form IKK_i, which is converted to IKK_{ii} and then to the initial form IKK_n. Active IKK_a phosphorylates IκBα, priming it for degradation (phosphorylation increases degradation rate of free IκBα 100 times and of IκBα complexed with NF-κB 500 times). IκBα degradation results in NF-κB release and its nuclear translocation. There are the following translocation rules: unbound NF-κB translocates to the nucleus; IκBα–NF-κB complexes translocate to the cytoplasm; free unphosphorylated IκBα may translocate between these two cell compartments. Nuclear NF-κB binds gene promoters and activates transcription of *Nfkbia* (IκBα), *Tnfaip3* (A20), *Tnf*, *Il6,* and *Ccl2* genes.

IκBα and A20 mediate two negative feedback loops: IκBα synthesized in the cytoplasm enters the nucleus, binds NF-κB and the complex translocates to the cytoplasm. A20 inhibits IKK_a by increasing conversion rate from IKK_a to IKK_i. A20 also inhibits TBK1 activity (by disrupting the TBK1–TRAF3–RIG-I–MAVS complex[5], see below). Although LPS-bound TLR4 receptor phosphorylates TBK1, TBK1 phosphorylation does not lead to IRF3 phosphorylation, which requires a scaffolding protein. For the sake of clarity, this dead-end LPS-induced TBK1 phosphorylation is not shown in the model scheme in Fig 6A in the main text.

IFNβ stimulation. IFNβ stimulation leads to the activation of IFNAR followed by STAT1 and STAT2 phosphorylation and dimerization. STAT1 and STAT2 can form heterodimers STAT1–STAT2, or homodimers STAT2–STAT2 which have 10 times lower binding rate than the heterodimers. STAT hetero- and homodimers enter the nucleus and activate *Socs1*, *Stat1*, *Stat2*, *Pkr*, *Oas1a*, *Ddx58* (RIG-I), *Ifit1* (ISG56), *Ifit2* (ISG54), *Irf1*, *Irf5*, *Irf7*, and *Isg15* genes. SOCS1 mediates negative feedback limiting STAT signaling by binding to active IFNAR receptors and deactivating them.

Poly(I:C) stimulation. Poly(I:C) binds RIG-I–MAVS complex. Poly(I:C)–RIG-I–MAVS complex activates TAK1, which leads to the activation of the NF-κB pathway, as described for LPS signaling. This complex also binds TRAF3[6] and TBK1, leading to phosphorylation of TBK1. Poly(I:C)–RIG-I–MAVS–TRAF3–p-TBK1 complex phosphorylates IRF3. Protein TRAF3, which enables phosphorylation of IRF3 by TBK1, is not shown in the model schematic in Fig 6A in the main text for the sake of simplicity. Phosphorylated IRF3 forms dimers which translocate to the nucleus. IRF3 dimers and NF-κB bind IFNβ promoter; simultaneous binding of these two transcription factors triggers IFNβ transcription. Synthetized IFNβ is secreted and may immediately bind cell receptors (autocrine signaling) or accumulates in the extracellular space. Extracellular IFNβ may also bind IFNAR, as in the case of IFNβ stimulation (paracrine signaling). This simplified model of paracrine signaling is a reasonable approximation in the case of homogeneous cell population. However, it underestimates paracrine signaling in the case of heterogeneous populations, where cells which do not produce IFNβ can be activated by IFNβ secreted by other cells. The realistic modeling of paracrine signaling in heterogenous populations is problematic since it depends on diffusivity, which is very different in the tissue and in the experimental setup.

As said above, IFNβ signaling leads to STAT activation and transcription of OAS1A and PKR. OAS1A and PKR proteins are activated by poly(I:C) and inhibit IκBα and A20. Active OAS1A enhances degradation rate of IκBα and A20 transcripts, while active PKR disrupts translation of these two proteins. In this way PKR

and OAS1A mediate a positive feedback by inhibiting the inhibitors of transcription factors NF-κB and IRF3. For the sake of simplicity, we do not explicitly consider the influence of OAS1A and PKR on synthesis of other proteins. We expect that this influence is strongest in the case of A20 and IκBα, because these proteins have a relatively fastest turnover; the influence on other components is approximately accounted for by fitting their transcription and translation coefficients to the experimental data.

To account for the resting cell activity of NF-κB and IRF3, we assume small basal phosphorylation rates of TAK1 and IRF3.

1.3 Gene regulation and transcription

We assume that each gene is present in two copies and the states of all gene copies are regulated (independently) by binding of transcription factors: NF-κB, IRF3, STAT1–STAT2 and STAT2–STAT2:

1) NF-κB activates *Nfkbia* (IκBα), *Tnfaip3* (A20), *Tnf*, *Il6* and *Ccl2* genes and, cooperatively with IRF3, *Ifnb* gene;
2) IRF3 homodimers activate *Ccl5* (RANTES) gene and, cooperatively with NF-κB, *Ifnb1* gene;
3) STAT1–STAT2 and STAT2–STAT2 dimers activate *Stat1*, *Stat2*, *Pkr*, *Oas1a*, *Socs1*, *Ddx58* (RIG-I), *Ifit1* (ISG56), *Ifit2* (ISG54), *Irf1*, *Irf5*, *Irf7, Ccl2* and *Isg15* genes.

When a gene is OFF, the transcription is fully inhibited. *Stat1*, *Stat2*, *Ifit2* (ISG54), and *Irf1* genes can be activated by STAT dimers but also spontaneously (i.e., by binding of some implicit transcription factors, not included in the model). Such activation is necessary to obtain non-zero protein levels observed in the experiments in non-stimulated cells.

Activation of *Tnf*, *Il6* and *Ccl2* genes by NF-κB is cooperative with the coefficient of cooperativity (number of NF-κB molecules bound to the gene promoter necessary to activate the gene) assumed 4 for *Tnf* and *Il6* genes, and 2 for *Ccl2* gene. Such cooperativity coefficients (or additional transcription factors) are necessary to reproduce large (when compared to *Nfkbia* (IκBα) or *Tnfaip3* (A20)) increase of mRNA levels after stimulation.

For the sake of model simplicity, some proteins are assumed to have constant expression level (see Table B). For readout genes, i.e., for genes that code for proteins that have no regulatory role within the proposed model (see Fig 6A), we consider only the steps of gene regulation and mRNA transcription. The obtained mRNA profiles are then compared to the experimental PCR data. The *Irf7* gene, expression of which increases significantly after IFNβ or poly(I:C) stimulation, was considered as a readout gene, because we were unable to detect any increase of IRF7 protein level in Western blots using antibodies from Abcam (ab10925) and ThermoFisher (PA5-20280). We also did not observe any growth of phospho-IRF7 level using an antibody from Cell Signaling (#5184).

1.4 Numerical simulation protocols

Model validation was based on stochastic simulations employing Gillespie algorithm[7] as implemented in BioNetGen[1]. To compare with the population-type data coming from Western blots and PCRs, we averaged

over 200 independent stochastic simulation trajectories. Due to high nonlinearity of the system, the stochastic average does not match the deterministic approximation. To account for initial population heterogeneity, each simulation was preceded with 3,000,000 s (ca. 35 days) long equilibration phase.

In Supplementary Fig. 4b,c we show distributions of the levels of RIG-I and PKR after equilibration. The variability of initial RIG-I and PKR levels at the time of poly(I:C) delivery results in heterogeneity of cell responses. The deterministic simulations, in which initial RIG-I and PKR levels are drawn from distributions shown in Supplementary Fig. 4b,c, lead to heterogeneous responses (Supplementary Fig. 4d); however, the distribution of nuclear IRF3 and NF-κB levels is different from that obtained in stochastic simulations. For the sake of simplicity, in model simulations the total amounts of NF-κB and IRF3 are constant and equal 10^5. In reality, these levels vary among cells and consequently cells with full translocation of NF-κB and IRF3 show broad distributions of nuclear NF-κB and IRF3 levels (cf. Supplementary Fig. 4a and 4d,e,f).

After the equilibration phase one of the stimuli was applied: LPS, IFNβ, or poly(I:C). We also used a simulation protocol in which the 24 hr-long IFNβ stimulation was followed by poly(I:C) stimulation. In order to simulate experimental protocols in which IFNAR was inhibited, we reduced the number of IFNAR receptors to 20% of the default value; see also Table B.

1.5 Model components and structure

Table A. Molecular species.

Symbol	Description
	Signaling molecules
$Liposome_{ext}$, $Liposome_{cyt}$	Liposomes with poly(I:C), extracellular or cytoplasmic
$PolyIC$	Poly(I:C) molecule; may activate MAVS:RIG-I, PKR, OAS1A
LPS	LPS molecule; may activate receptor TLR4
$IFNb_{cyt}$, $IFNb_{ext}$	Cytoplasmic or extracellular IFNβ; may activate receptor IFNAR
	Receptors
$TLR4$	Inactive TLR4
$TLR4{:}LPS$	Active TLR4 with LPS bound
RIG	RIG-I protein
$MAVS$	MAVS protein
$MAVS{:}RIG$	Complex of MAVS and RIG-I
$MAVS{:}RIG{:}PolyIC$	Complex of MAVS, RIG-I and poly(I:C)
$IFNAR$	IFNAR (inactive)
$IFNAR{:}IFNb$	IFNAR with IFNβ bound (active)
$IFNAR{:}IFNb{:}SOCS1$	IFNAR with both IFNβ and repressor SOCS1 bound (inactive)
$IFNAR{:}SOCS1$	IFNAR with only repressor SOCS1 bound (inactive)
	Other proteins
$SOCS1$	SOCS1; repressor of IFNAR
$TBK1$	Unphosphorylated TBK1
$pTBK1$	TBK1 phosphorylated at Ser172
$TAK1_{inactive}$	Inactive TAK1
$TAK1_{active}$	Active TAK1
$TRAF3$	TRAF3
$MAVS{:}RIG{:}PolyIC{:}TRAF3$	Complex of poly(I:C), RIG-I, MAVS and TRAF3
$MAVS{:}RIG{:}PolyIC{:}TRAF3{:}pTBK1$	Complex of poly(I:C), RIG-I, MAVS, TRAF3 and TBK1 phosphorylated at Ser172
$A20$	A20; repressor of IKK and TBK1
IKK_n	Inactive IKK protein complex
IKK_a	Active IKK protein complex; can phosphorylate IκBα
IKK_i	Inactive IKK protein complex (arises from IKK_a)
IKK_{ii}	Inactive IKK protein complex (arises from IKK_i)
$PKR_{inactive}$	PKR (inactive)
PKR_{active}	PKR phosphorylated at Thr451 (active); decreases translation rates of IκBα and A20
$OAS1A_{inactive}$	Inactive OAS1A protein
$OAS1A_{active}$	Active OAS1A; increases mRNA degradation rates of IκBα, A20
$IkBa_{cyt}$, $IkBa_{nuc}$	Unphosphorylated IκBα, cytoplasmic or nuclear (repressor of NF-κB)
$NFkB_{cyt}$	Cytoplasmic NF-κB
$NFkB_{nuc}$	Nuclear NF-κB (activates transcription)
$IkBa{:}NFkB_{cyt}$, $IkBa{:}NFkB_{nuc}$	Complex of IκBα and NF-κB, cytoplasmic or nuclear
$pIkBa_{cyt}$	Cytoplasmic phosphorylated IκBα

$pIkBa{:}NFkB_{cyt}$	Cytoplasmic phosphorylated IκBα in complex with NF-κB
$IRF3_{cyt}$, $IRF3_{nuc}$	Unphosphorylated IRF3, cytoplasmic or nuclear
$pIRF3_{cyt}$, $pIRF3_{nuc}$	IRF3 phosphorylated at Ser396, cytoplasmic or nuclear
$pIRF3{:}pIRF3_{cyt}$	Cytoplasmic phosphorylated IRF3 dimer
$pIRF3{:}pIRF3_{nuc}$	Nuclear phosphorylated IRF3 dimer (activates transcription)
$STAT1_{cyt}$, $STAT1_{nuc}$	Unphosphorylated STAT1, cytoplasmic or nuclear
$pSTAT1_{cyt}$, $pSTAT1_{nuc}$	STAT1 phosphorylated at Tyr701, cytoplasmic or nuclear
$STAT2_{cyt}$, $STAT2_{nuc}$	Unphosphorylated STAT2, cytoplasmic or nuclear
$pSTAT2_{cyt}$, $pSTAT2_{nuc}$	STAT2 phosphorylated at Tyr690, cytoplasmic or nuclear
$pSTAT1{:}pSTAT2_{cyt}$	Cytoplasmic STAT1-STAT2 heterodimer
$pSTAT1{:}pSTAT2_{nuc}$	Nuclear STAT1-STAT2 heterodimer (activates transcription)
$pSTAT2{:}pSTAT2_{cyt}$	Cytoplasmic STAT2-STAT2 homodimer
$pSTAT2{:}pSTAT2_{nuc}$	Nuclear STAT2-STAT2 homodimer (activates transcription)
Genes	
$IkBa_{gene\_off}$<br>$A20_{gene\_off}$<br>$IFNb_{gene\_off}$<br>PKR_{gene_off}<br>$OAS1A_{gene\_off}$<br>$IRF7_{gene\_off}$<br>$STAT1_{gene\_off}$<br>$STAT2_{gene\_off}$<br>RIG_{gene_off}<br>$SOCS1_{gene\_off}$<br>$TNFa_{gene\_off}$<br>$IL6_{gene\_off}$<br>$RANTES_{gene\_off}$<br>$CCL2_{gene\_off}$<br>$ISG54_{gene\_off}$<br>$ISG56_{gene\_off}$<br>$IRF1_{gene\_off}$<br>$IRF5_{gene\_off}$<br>$ISG15_{gene\_off}$	Transcriptionally non-active genes
$IFNb_{gene\_off,NFkB}$	IFNβ gene with only NF-κB bound to its promoter, transcriptionally inactive (because of the lack of IRF3)
$IFNb_{gene\_on,NFkB,IRF3}$	Transcriptionally active IFNβ gene (with both NF-κB and IRF3 bound to its promoter)
$TNFa_{gene\_off,NFkB\times1}$<br>$TNFa_{gene\_off,NFkB\times2}$<br>$TNFa_{gene\_off,NFkB\times3}$<br>$IL6_{gene\_off,NFkB\times1}$<br>$IL6_{gene\_off,NFkB\times2}$<br>$IL6_{gene\_off,NFkB\times3}$<br>$CCL2_{gene\_off,NFkB\times1}$	Transcriptionally inactive genes which require binding of two (Ccl2) or four (Tnf, Il6) NF-κB molecules to become active<br><br>[Digits at the end of subscripts denote the number of NF-κB molecules already bound to the gene promoter.]

$STAT1_{gene\_on}$<br>$STAT2_{gene\_on}$<br>$ISG54_{gene\_on}$<br>$IRF1_{gene\_on}$	Transcriptionally active genes, activated by transcription factors not included in the model
$IkBa_{gene\_on,NFkB}$<br>$A20_{gene\_on,NFkB}$<br>$RANTES_{gene\_on,IRF3}$<br>$PKR_{gene\_on,STAT1\_2}$, $PKR_{gene\_on,STAT2\_2}$<br>$OAS1A_{gene\_on,STAT1\_2}$, $OAS1A_{gene\_on,STAT2\_2}$<br>$IRF7_{gene\_on,STAT1\_2}$, $IRF7_{gene\_on,STAT2\_2}$<br>$STAT1_{gene\_on,STAT1\_2}$, $STAT1_{gene\_on,STAT2\_2}$<br>$STAT2_{gene\_on,STAT1\_2}$, $STAT2_{gene\_on,STAT2\_2}$<br>$RIG_{gene\_on,STAT1\_2}$, $RIG_{gene\_on,STAT2\_2}$<br>$SOCS1_{gene\_on,STAT1\_2}$, $SOCS1_{gene\_on,STAT2\_2}$<br>$CCL2_{gene\_on,STAT1\_2}$, $CCL2_{gene\_on,STAT2\_2}$<br>$CCL2_{gene\_on,STAT1\_2,NFkB\times1}$<br>$CCL2_{gene\_on,STAT2\_2,NFkB\times1}$<br>$CCL2_{gene\_on,STAT1\_2,NFkB\times2}$<br>$CCL2_{gene\_on,STAT2\_2,NFkB\times2}$<br>$ISG54_{gene\_on,STAT1\_2}$, $ISG54_{gene\_on,STAT2\_2}$<br>$ISG56_{gene\_on,STAT1\_2}$, $ISG56_{gene\_on,STAT2\_2}$<br>$IRF1_{gene\_on,STAT1\_2}$, $IRF1_{gene\_on,STAT2\_2}$<br>$IRF5_{gene\_on,STAT1\_2}$, $IRF5_{gene\_on,STAT2\_2}$<br>$ISG15_{gene\_on,STAT1\_2}$, $ISG15_{gene\_on,STAT2\_2}$	Transcriptionally active genes activated by NF-κB, IRF3 dimers, STAT1-STAT2 heterodimers, or STAT2-STAT2 homodimers<br><br>[When a STAT dimer is bound to Ccl2 gene, the gene is transcriptionally active irrespective of NF-κB binding.]
$TNFa_{gene\_on,NFkB\times4}$<br>$IL6_{gene\_on,NFkB\times4}$<br>$CCL2_{gene\_on,NFkB\times2}$	Transcriptionally active genes, activated cooperatively by NF-κB
mRNAs	
$IkBa_{mRNA}$<br>$A20_{mRNA}$<br>$IFNb_{mRNA}$<br>PKR_{mRNA}<br>$OAS1A_{mRNA}$<br>$STAT1_{mRNA}$, $STAT2_{mRNA}$<br>RIG_{mRNA}<br>$SOCS1_{mRNA}$<br>$TNFa_{mRNA}$<br>$IL6_{mRNA}$<br>$RANTES_{mRNA}$<br>$CCL2_{mRNA}$<br>$ISG54_{mRNA}$, $ISG56_{mRNA}$, $ISG15_{mRNA}$<br>$IRF1_{mRNA}$, $IRF5_{mRNA}$, $IRF7_{mRNA}$	Gene transcripts

Table B. Numbers of molecules per cell.

Parameter	Symbol	Value	Remarks
Number of IFNβ receptors	$ifnar_n$	10^4	
Number of NF-κB molecules	$nfkb_n$	10^5	
Number of TAK1 molecules	$tak1_n$	10^5	
Number of IKK molecules	ikk_n	2×10^5	
Number of TBK1 molecules	$tbk1_n$	10^5	
Number of TRAF3 molecules	$traf3_n$	10^5	
Number of MAVS molecules	$mavs_n$	5×10^5	
Ratio of cytoplasmic to nuclear volume	k_v	5	
LPS stimulation			
Number of LPS molecules	lps_n	10^6 × dose in µg/ml	10^6 corresponds to 1 µg/ml, initial condition
poly(I:C) stimulation			
Number of poly(I:C) molecules	$polyIC_n$	10^5 × dose in µg/ml	10^5 corresponds to 1 µg/ml, initial condition
Number of liposomes per 10 cells	$liposome_n$	100	on average 10 liposomes enter a cell
IFNβ stimulation			
Number of IFNβ molecules	$ifnb_{initial}$	10^6	10^6 corresponds to 1000 U/ml, initial condition
Simulation with IFNβ receptors inhibited (see Fig. 4a,c)			
Number of IFNβ receptors	$ifnar_n$	2×10^3	corresponds to 20% of receptors not blocked by α-IFNAR
Simulations of *Stat1*$^{-/-}$ cells (see Fig. 4a,c)			
Number of *Stat1* gene copies	–	0	

Table C. Denotational conventions.

Conventions listed below are used in Table D, which starts on the next page.

Expression	Meaning
$[X]$	Number of molecules of X per cell
S_X	Synthesis rate of protein X
T_X	Transcription rate of a gene coding for protein X
L_X	Degradation (Loss) rate of molecule X
I_X	Import rate of molecule X from the cytoplasm to the nucleus
$V_{liposome}$	Rate of the intake of liposomes (containing Virus-mimicking poly(I:C)) into the cytoplasm
V_{polyIC}	Rate of the intake of poly(I:C) (a Virus-mimicking agent) from liposomes into the cytoplasm
E_X	Export rate of molecules X from the nucleus to the cytoplasm
C_{IFNb}	Cytokine (IFNβ) secretion rate by the cell
B_X	Binding rate of signaling molecule X to its receptor
B_{X_Y}	Binding rate of two molecules, X and Y
U_X	Unbinding rate of signaling molecule X from its receptor
U_{X_Y}	Unbinding rate of two molecules, X and Y
$U_{X\_Y,Z}$	Unbinding rate of two molecules, X and Y, triggered by Z
A_X	Activation rate of X, “spontaneous”
$A_{X,Y}$	Activation rate of protein X, by protein Y
A_{X_g}	Activation rate of a gene coding for protein X, “spontaneous”
$A_{X\_g,Y}$	Activation rate of a gene coding for protein X, by protein Y
D_X	Deactivation rate of X, “spontaneous”
$D_{X,Y}$	Deactivation rate of protein X, by protein Y
D_{X_g}	Deactivation rate of a gene coding for protein X, “spontaneous”
$D_{X\_g,Y}$	Deactivation rate of a gene coding for protein X, by protein Y

Table D. Reactions and coefficients.
All rates of first-order reactions are given in [1/s]; rates of second-order reactions are given in [1/s/molecule].

Reaction	Rate	Coeff(s)	Value
Import of poly(I:C) to the cytoplasm			
$Liposome_{ext} \rightarrow Liposome_{cyt}$	$V_{liposome}$	$V_{liposome}$	10^{-5}
$Liposome_{cyt} \rightarrow Liposome_{cyt} + PolyIC$	V_{polyIC}<br>(V_{polyIC} is defined by average number of poly(I:C) per cell: $polyIC_n$)	V_{polyIC}	3 × dose in µg/ml
$Liposome_{ext} \rightarrow \emptyset$	$L_{liposome\_ext} = 9 \cdot V_{liposome}$<br>(import of liposomes by 9 other cells)	$L_{liposome\_ext}$	9×10^{-5}
$Liposome_{cyt} \rightarrow \emptyset$	$L_{liposome\_cyt}$	$L_{liposome\_cyt}$	3×10^{-4}
$PolyIC \rightarrow \emptyset$	L_{polyIC}	L_{polyIC}	5×10^{-4}
Activation of receptors			
$LPS + TLR4 \rightarrow TLR4{:}\,LPS$	B_{LPS}	B_{LPS}	10^{-9}
$LPS + TLR4 \leftarrow TLR4{:}\,LPS$	U_{LPS}	U_{LPS}	10^{-4}
$TLR4{:}\,LPS \rightarrow \emptyset$	L_{TLR4_LPS}	L_{TLR4_LPS}	2×10^{-4}
$RIG + MAVS \rightarrow MAVS{:}\,RIG$	B_{RIG_MAVS}	B_{RIG_MAVS}	10^{-7}
$RIG + MAVS \leftarrow MAVS{:}\,RIG$	U_{RIG_MAVS}	U_{RIG_MAVS}	10^{-3}
$PolyIC + MAVS{:}\,RIG \rightarrow MAVS{:}\,RIG{:}\,PolyIC$	B_{polyIC}	B_{polyIC}	10^{-8}
$PolyIC + MAVS{:}\,RIG \leftarrow MAVS{:}\,RIG{:}\,PolyIC$	U_{polyIC}	U_{polyIC}	10^{-3}
$IFNb_{cyt} \rightarrow IFNb_{ext}$	C_{IFNb}	C_{IFNb}	10^{-4}
$IFNAR + IFNb_{cyt} \rightarrow IFNAR{:}\,IFNb$	B_{IFNb_cyt}	B_{IFNb_cyt}	10^{-7}
$IFNAR + IFNb_{ext} \rightarrow IFNAR{:}\,IFNb$	B_{IFNb_ext}	B_{IFNb_ext}	10^{-9}
$IFNAR + IFNb_{ext} \leftarrow IFNAR{:}\,IFNb$	U_{IFNb}	U_{IFNb}	10^{-3}
$IFNAR{:}\,IFNb + SOCS1 \rightarrow IFNAR{:}\,IFNb{:}\,SOCS1$	B_{SOCS1}	B_{SOCS1}	10^{-7}
$IFNAR{:}\,IFNb{:}\,SOCS1 \rightarrow IFNAR{:}\,IFNb + SOCS1$	U_{SOCS1}	U_{SOCS1}	3×10^{-4}
$IFNAR{:}\,SOCS1 \rightarrow IFNAR + SOCS1$			

Signal transduction			
$TLR4{:}LPS + TBK1 \rightarrow TLR4{:}LPS + pTBK1$	$A_{TBK1,TLR4}$	$A_{TBK1,TLR4}$	3×10^{-8}
$TLR4{:}LPS + TAK1_{inactive} \rightarrow TLR4{:}LPS + TAK1_{active}$	$A_{TAK1,TLR4}$	$A_{TAK1,TLR4}$	3×10^{-8}
$TAK1_{inactive} \rightarrow TAK1_{active}$	A_{TAK1}	A_{TAK1}	10^{-5}
$TRAF3 + MAVS{:}RIG{:}PolyIC \rightarrow MAVS{:}RIG{:}PolyIC{:}TRAF3$	B_{MAVS_TRAF3}	B_{MAVS_TRAF3}	10^{-7}
$TRAF3 + MAVS{:}RIG{:}PolyIC \leftarrow MAVS{:}RIG{:}PolyIC{:}TRAF3$	U_{MAVS_TRAF3}	U_{MAVS_TRAF3}	10^{-3}
$TBK1 + MAVS{:}RIG{:}PolyIC{:}TRAF3 \rightarrow MAVS{:}RIG{:}PolyIC{:}TRAF3{:}pTBK1$	B_{MAVS_TBK1}	B_{MAVS_TBK1}	3×10^{-8}
$pTBK1 + MAVS{:}RIG{:}PolyIC{:}TRAF3 \rightarrow MAVS{:}RIG{:}PolyIC{:}TRAF3{:}pTBK1$			
$pTBK1 + MAVS{:}RIG{:}PolyIC{:}TRAF3 \leftarrow MAVS{:}RIG{:}PolyIC{:}TRAF3{:}pTBK1$	U_{MAVS_TBK1}	U_{MAVS_TBK1}	10^{-3}
$pTBK1 \rightarrow TBK1$	D_{TBK1}	D_{TBK1}	10^{-4}
$MAVS{:}RIG{:}PolyIC{:}TRAF3 \rightarrow MAVS{:}RIG + PolyIC + TRAF3$	U_{PolyIC}	U_{PolyIC}	10^{-3}
$MAVS{:}RIG{:}PolyIC{:}TRAF3{:}pTBK1 \rightarrow MAVS{:}RIG + PolyIC + TRAF3 + pTBK1$			
$MAVS{:}RIG{:}PolyIC{:}TRAF3{:}pTBK1 + A20 \rightarrow pTBK1 + MAVS{:}RIG{:}PolyIC{:}TRAF3 + A20$	$U_{MAVS\_TBK1,A20}$	$U_{MAVS\_TBK1,A20}$	10^{-8}
$MAVS{:}RIG{:}PolyIC + TAK1_{inactive} \rightarrow MAVS{:}RIG{:}PolyIC + TAK1_{active}$	$A_{TAK1,MAVS}$	$A_{TAK1,MAVS}$	3×10^{-8}
$MAVS{:}RIG{:}PolyIC{:}TRAF3 + TAK1_{inactive} \rightarrow MAVS{:}RIG{:}PolyIC{:}TRAF3 + TAK1_{active}$			
$MAVS{:}RIG{:}PolyIC{:}TRAF3{:}pTBK1 + TAK1_{inactive} \rightarrow MAVS{:}RIG{:}PolyIC{:}TRAF3{:}pTBK1 + TAK1_{active}$			
$TAK1_{active} \rightarrow TAK1_{inactive}$	D_{TAK1}	D_{TAK1}	10^{-2}
$IKK_n \xrightarrow{TAK1_{\text{active}}} IKK_a$	$A_{IKK} \cdot [TAK1_{active}]^2$	A_{IKK}	6×10^{-10}
$IKK_a \xrightarrow{A20} IKK_i$	$(D_{IKK2} + [A20]) \cdot \frac{D_{IKK1}}{D_{IKK2}}$	D_{IKK1}<br>D_{IKK2}	2×10^{-3}<br>10^{4}
$IKK_i \rightarrow IKK_{ii}$	D_{IKK3}	D_{IKK3}	10^{-3}
$IKK_{ii} \rightarrow IKK_n$			
$PolyIC +\ PKR_{inactive} \rightarrow PolyIC +\ PKR_{active}$	A_{PKR}	A_{PKR}	10^{-8}
$PolyIC +\ OAS1A_{inactive} \rightarrow PolyIC +\ OAS1A_{active}$	A_{OAS1A}	A_{OAS1A}	3×10^{-8}

NF-κB module			
$IkBa_{cyt} + NFkB_{cyt} \rightarrow IkBa{:}NFkB_{cyt}$	$B_{NFkB\_IkBa\_cyt}$	$B_{NFkB\_IkBa\_cyt}$	5×10^{-7}
$IkBa_{nuc} + NFkB_{nuc} \rightarrow IkBa{:}NFkB_{nuc}$	$B_{NFkB\_IkBa\_nuc} = k_v \cdot B_{NFkB\_IkBa\_cyt}$	$B_{NFkB\_IkBa\_nuc}$	2.5×10^{-6}
$IKK_a + IkBa_{cyt} \rightarrow IKK_a + pIkBa_{cyt}$	$D_{IkBa,IKK}$	$D_{IkBa,IKK}$	10^{-7}
$IKK_a + IkBa{:}NFkB_{cyt} \rightarrow IKK_a + pIkBa{:}NFkB_{cyt}$	$D_{NFkB\_IkBa,IKK}$	$D_{NFkB\_IkBa,IKK}$	5×10^{-7}
$pIkBa_{cyt} \rightarrow \emptyset$	L_{pIkBa}	L_{pIkBa}	10^{-2}
$pIkBa{:}NFkB_{cyt} \rightarrow NFkB_{cyt}$			
$IkBa_{cyt} \rightarrow \emptyset$	L_{IkBa}	L_{IkBa}	10^{-4}
$IkBa{:}NFkB_{cyt} \rightarrow NFkB_{cyt}$	$L_{NFkB,IkBa}$	$L_{NFkB,IkBa}$	2×10^{-5}
$A20 \rightarrow \emptyset$	L_{A20}	L_{A20}	5×10^{-4}
$NFkB_{cyt} \rightarrow NFkB_{nuc}$	I_{NFkB}	I_{NFkB}	10^{-2}
$IkBa{:}NFkB_{nuc} \rightarrow IkBa{:}NFkB_{cyt}$	$E_{NFkB,IkBa}$	$E_{NFkB,IkBa}$	5×10^{-2}
$IkBa_{cyt} \rightarrow IkBa_{nuc}$	I_{IkBa}	I_{IkBa}	2×10^{-3}
$IkBa_{cyt} \leftarrow IkBa_{nuc}$	E_{IkBa}	E_{IkBa}	5×10^{-3}
$NFkB_{nuc} + IkBa_{gene\_off} \rightarrow IkBa_{gene\_on,NFkB}$	$A_{IkBa\_g,NFkB}$	$A_{IkBa\_g,NFkB}$	4×10^{-7}
$IkBa_{nuc} + IkBa_{gene\_on,NFkB} \rightarrow IkBa_{gene\_off} + IkBa{:}NFkB_{nuc}$	$D_{IkBa\_g,IkBa}$	$D_{IkBa\_g,IkBa}$	10^{-6}
$NFkB_{nuc} + A20_{gene\_off} \rightarrow A20_{gene\_on,NFkB}$	$A_{A20\_g,NFkB}$	$A_{A20\_g,NFkB}$	10^{-7}
$IkBa_{nuc} + A20_{gene\_on,NFkB} \rightarrow A20_{gene\_off} + IkBa{:}NFkB_{nuc}$	$D_{A20\_g,IkBa}$	$D_{A20\_g,IkBa}$	10^{-6}
$IkBa_{gene\_on,NFkB} \rightarrow IkBa_{gene\_on,NFkB} + IkBa_{mRNA}$	T_{IkBa}	T_{IkBa}	10^{-1}
$A20_{gene\_on,NFkB} \rightarrow A20_{gene\_on,NFkB} + A20_{mRNA}$	T_{A20}	T_{A20}	10^{-1}
$IkBa_{mRNA} \xrightarrow{OAS1A_{active}} \emptyset$	$L_{IkBa\_mRNA} \cdot \frac{M_{OAS1A} + [OAS1A_{active}]}{M_{OAS1A}}$	L_{IkBa_mRNA}<br>M_{OAS1A}	7.5×10^{-4}<br>10^{4}
$A20_{mRNA} \xrightarrow{OAS1A_{active}} \emptyset$	$L_{A20\_mRNA} \cdot \frac{M_{OAS1A} + [OAS1A_{active}]}{M_{OAS1A}}$	L_{A20_mRNA}<br>M_{OAS1A}	7.5×10^{-4}<br>10^{4}

$IkBa_{mRNA} \rightarrow IkBa_{mRNA} + IkBa_{cyt}$	$S_{IkBa} \cdot \frac{M_{PKR}}{M_{PKR} + [PKR_{active}]}$	S_{IkBa}<br>M_{PKR}	5×10^{-1}<br>3×10^{4}
$A20_{mRNA} \rightarrow A20_{mRNA} + A20$	$S_{A20} \cdot \frac{M_{PKR}}{M_{PKR} + [PKR_{active}]}$	S_{A20}<br>M_{PKR}	5×10^{-1}<br>3×10^{4}
IRF3 pathway			
$MAVS{:}RIG{:}PolyIC{:}TRAF3{:}pTBK1 + IRF3_{cyt} \rightarrow MAVS{:}RIG{:}PolyIC{:}TRAF3{:}pTBK1 + pIRF3_{cyt}$	$A_{IRF3,TBK1}$	$A_{IRF3,TBK1}$	3×10^{-7}
$IRF3_{cyt} \rightarrow pIRF3_{cyt}$	A_{IRF3}	A_{IRF3}	1.3×10^{-5}
$IRF3_{cyt} \leftarrow pIRF3_{cyt}$	D_{IRF3}	D_{IRF3}	10^{-3}
$IRF3_{nuc} \leftarrow pIRF3_{nuc}$			
$pIRF3_{cyt} + pIRF3_{cyt} \rightarrow pIRF3{:}pIRF3_{cyt}$	$B_{IRF3\_IRF3\_cyt}$	$B_{IRF3\_IRF3\_cyt}$	10^{-6}
$pIRF3_{nuc} + pIRF3_{nuc} \rightarrow pIRF3{:}pIRF3_{nuc}$	$B_{IRF3\_IRF3\_nuc} = k_v \cdot B_{IRF3\_IRF3\_cyt}$	$B_{IRF3\_IRF3\_nuc}$	5×10^{-6}
$pIRF3_{cyt} + pIRF3_{cyt} \leftarrow pIRF3{:}pIRF3_{cyt}$	U_{IRF3_IRF3}	U_{IRF3_IRF3}	10^{-3}
$pIRF3_{nuc} + pIRF3_{nuc} \leftarrow pIRF3{:}pIRF3_{nuc}$			
$pIRF3{:}pIRF3_{cyt} \rightarrow pIRF3{:}pIRF3_{nuc}$	I_{IRF3_IRF3}	I_{IRF3_IRF3}	10^{-2}
$IRF3_{nuc} \rightarrow IRF3_{cyt}$	E_{IRF3}	E_{IRF3}	10^{-3}
$NFkB_{nuc} + IFNb_{gene\_off} \rightarrow IFNb_{gene\_off,NFkB}$	$A_{IFNb\_g,NFkB}$	$A_{IFNb\_g,NFkB}$	4×10^{-7}
$IkBa_{nuc} + IFNb_{gene\_off,NFkB} \rightarrow IFNb_{gene\_off} + IkBa{:}NFkB_{nuc}$	$D_{IFNb\_g\_NFkB,IkBa}$	$D_{IFNb\_g\_NFkB,IkBa}$	10^{-6}
$pIRF3{:}pIRF3_{nuc} + IFNb_{gene\_off,NFkB} \rightarrow IFNb_{gene\_on,NFkB,IRF3}$	$A_{IFNb\_g\_NFkB,IRF3}$	$A_{IFNb\_g\_NFkB,IRF3}$	3×10^{-8}
$IFNb_{gene\_on,NFkB,IRF3} \rightarrow IFNb_{gene\_off} + NFkB_{nuc} + pIRF3{:}pIRF3_{nuc}$	$D_{IFNb\_g\_NFkB\_IRF3}$	$D_{IFNb\_g\_NFkB\_IRF3}$	10^{-3}
$IFNb_{gene\_on,NFkB,IRF3} \rightarrow IFNb_{gene\_on,NFkB,IRF3} + IFNb_{mRNA}$	T_{IFNb}	T_{IFNb}	1.5×10^{-2}
$IFNb_{mRNA} \rightarrow \emptyset$	L_{IFNb_mRNA}	L_{IFNb_mRNA}	3×10^{-4}
$IFNb_{mRNA} \rightarrow IFNb_{mRNA} + IFNb_{cyt}$	S_{IFNb}	S_{IFNb}	10^{-1}

STAT pathway			
$STAT1_{cyt} \xrightarrow{IFNAR:IFNb} pSTAT1_{cyt}$	$A_{STAT} \cdot \frac{M_{IFNb} \cdot [IFNAR:IFNb]}{M_{IFNb} + [STAT1_{cyt}] + [STAT2_{cyt}]}$	A_{STAT}	3×10^{-7}
$STAT2_{cyt} \xrightarrow{IFNAR:IFNb} pSTAT2_{cyt}$		M_{IFNb}	3×10^{4}
$pSTAT1_{cyt} \rightarrow STAT1_{cyt}$	D_{STAT1}	D_{STAT1}	10^{-3}
$pSTAT2_{cyt} \rightarrow STAT2_{cyt}$	D_{STAT2}	D_{STAT2}	10^{-3}
$pSTAT1_{cyt} + pSTAT2_{cyt} \rightarrow pSTAT1{:}\,pSTAT2_{cyt}$	$B_{STAT1\_STAT2\_cyt}$	$B_{STAT1\_STAT2\_cyt}$	10^{-5}
$pSTAT1_{nuc} + pSTAT2_{nuc} \rightarrow pSTAT1{:}\,pSTAT2_{nuc}$	$B_{STAT1\_STAT2\_nuc} = k_v \cdot B_{STAT1\_STAT2\_cyt}$	$B_{STAT1\_STAT2\_nuc}$	5×10^{-5}
$pSTAT1_{cyt} + pSTAT2_{cyt} \leftarrow pSTAT1{:}\,pSTAT2_{cyt}$	U_{STAT1_STAT2}	U_{STAT1_STAT2}	3×10^{-3}
$pSTAT1_{nuc} + pSTAT2_{nuc} \leftarrow pSTAT1{:}\,pSTAT2_{nuc}$			
$pSTAT2_{cyt} + pSTAT2_{cyt} \rightarrow pSTAT2{:}\,pSTAT2_{cyt}$	$B_{STAT2\_STAT2\_cyt}$	$B_{STAT2\_STAT2\_cyt}$	10^{-6}
$pSTAT2_{nuc} + pSTAT2_{nuc} \rightarrow pSTAT2{:}\,pSTAT2_{nuc}$	$B_{STAT2\_STAT2\_nuc} = k_v \cdot B_{STAT2\_STAT2\_cyt}$	$B_{STAT2\_STAT2\_nuc}$	5×10^{-6}
$pSTAT2_{cyt} + pSTAT2_{cyt} \leftarrow pSTAT2{:}\,pSTAT2_{cyt}$	U_{STAT2_STAT2}	U_{STAT2_STAT2}	3×10^{-3}
$pSTAT2_{nuc} + pSTAT2_{nuc} \leftarrow pSTAT2{:}\,pSTAT2_{nuc}$			
$pSTAT1{:}\,pSTAT2_{cyt} \rightarrow pSTAT1{:}\,pSTAT2_{nuc}$	I_{STAT}	I_{STAT}	10^{-2}
$pSTAT2{:}\,pSTAT2_{cyt} \rightarrow pSTAT2{:}\,pSTAT2_{nuc}$			
$STAT1_{nuc} \rightarrow STAT1_{cyt}$	E_{STAT1}	E_{STAT1}	10^{-3}
$STAT2_{nuc} \rightarrow STAT2_{cyt}$	E_{STAT2}	E_{STAT2}	10^{-3}
Gene regulation			
$pSTAT1{:}\,pSTAT2_{nuc} + PKR_{gene\_off} \rightarrow PKR_{gene\_on,STAT1\_2}$	$A_{PKR\_g,STAT1\_2}$	$A_{PKR\_g,STAT1\_2}$	10^{-5}
$pSTAT2{:}\,pSTAT2_{nuc} + PKR_{gene\_off} \rightarrow PKR_{gene\_on,STAT2\_2}$	$A_{PKR\_g,STAT2\_2}$	$A_{PKR\_g,STAT2\_2}$	10^{-5}
$pSTAT1{:}\,pSTAT2_{nuc} + PKR_{gene\_off} \leftarrow PKR_{gene_{on},STAT1\_2}$	D_{PKR_g}	D_{PKR_g}	10^{-2}
$pSTAT2{:}\,pSTAT2_{nuc} + PKR_{gene\_off} \leftarrow PKR_{gene\_on,STAT2\_2}$			
$pSTAT1{:}\,pSTAT2_{nuc} + OAS1A_{gene\_off} \rightarrow OAS1A_{gene\_on,STAT1\_2}$	$A_{OAS1A\_g,STAT1\_2}$	$A_{OAS1A\_g,STAT1\_2}$	10^{-6}
$pSTAT2{:}\,pSTAT2_{nuc} + OAS1A_{gene\_off} \rightarrow OAS1A_{gene\_on,STAT2\_2}$	$A_{OAS1A\_g,STAT2\_2}$	$A_{OAS1A\_g,STAT2\_2}$	10^{-6}

$pSTAT1{:}\, pSTAT2_{nuc} + OAS1A_{gene\_off} \leftarrow OAS1A_{gene\_on,STAT1\_2}$	D_{OAS1A_g}	D_{OAS1A_g}	10^{-2}
$pSTAT2{:}\, pSTAT2_{nuc} + OAS1A_{gene\_off} \leftarrow OAS1A_{gene\_on,STAT2\_2}$			
$pSTAT1{:}\, pSTAT2_{nuc} + IRF7_{gene\_off} \rightarrow IRF7_{gene\_on,STAT1\_2}$	$A_{IRF7\_g,STAT1\_2}$	$A_{IRF7\_g,STAT1\_2}$	3×10^{-7}
$pSTAT2{:}\, pSTAT2_{nuc} + IRF7_{gene\_off} \rightarrow IRF7_{gene\_on,STAT2\_2}$	$A_{IRF7\_g,STAT2\_2}$	$A_{IRF7\_g,STAT2\_2}$	3×10^{-7}
$pSTAT1{:}\, pSTAT2_{nuc} + IRF7_{gene\_off} \leftarrow IRF7_{gene\_on,STAT1\_2}$	D_{IRF7_g}	D_{IRF7_g}	10^{-2}
$pSTAT2{:}\, pSTAT2_{nuc} + IRF7_{gene\_off} \leftarrow IRF7_{gene\_on,STAT2\_2}$			
$STAT1_{gene\_off} \rightarrow STAT1_{gene\_on}$	A_{STAT1_g}	A_{STAT1_g}	10^{-4}
$pSTAT1{:}\, pSTAT2_{nuc} + STAT1_{gene\_off} \rightarrow STAT1_{gene\_on,STAT1\_2}$	$A_{STAT1\_g,STAT1\_2}$	$A_{STAT1\_g,STAT1\_2}$	3×10^{-7}
$pSTAT2{:}\, pSTAT2_{nuc} + STAT1_{gene\_off} \rightarrow STAT1_{gene\_on,STAT2\_2}$	$A_{STAT1\_g,STAT2\_2}$	$A_{STAT1\_g,STAT2\_2}$	3×10^{-7}
$STAT1_{gene\_off} \leftarrow STAT1_{gene\_on}$	D_{STAT1_g}	D_{STAT1_g}	10^{-2}
$pSTAT1{:}\, pSTAT2_{nuc} + STAT1_{gene\_off} \leftarrow STAT1_{gene\_on,STAT1\_2}$			
$pSTAT2{:}\, pSTAT2_{nuc} + STAT1_{gene\_off} \leftarrow STAT1_{gene\_on,STAT2\_2}$			
$STAT2_{gene\_off} \rightarrow STAT2_{gene\_on}$	A_{STAT2_g}	A_{STAT2_g}	10^{-4}
$pSTAT1{:}\, pSTAT2_{nuc} + STAT2_{gene\_off} \rightarrow STAT2_{gene\_on,STAT1\_2}$	$A_{STAT2\_g,STAT1\_2}$	$A_{STAT2\_g,STAT1\_2}$	3×10^{-7}
$pSTAT2{:}\, pSTAT2_{nuc} + STAT2_{gene\_off} \rightarrow STAT2_{gene\_on,STAT2\_2}$	$A_{STAT2\_g,STAT2\_2}$	$A_{STAT2\_g,STAT2\_2}$	3×10^{-7}
$STAT2_{gene\_off} \leftarrow STAT2_{gene\_on}$	D_{STAT2_g}	D_{STAT2_g}	10^{-2}
$pSTAT1{:}\, pSTAT2_{nuc} + STAT2_{gene\_off} \leftarrow STAT2_{gene\_on,STAT1\_2}$			
$pSTAT2{:}\, pSTAT2_{nuc} + STAT2_{gene\_off} \leftarrow STAT2_{gene\_on,STAT2\_2}$			
$pSTAT1{:}\, pSTAT2_{nuc} + RIG_{gene\_off} \rightarrow RIG_{gene\_on,STAT1\_2}$	$A_{RIG\_g,STAT1\_2}$	$A_{RIG\_g,STAT1\_2}$	10^{-6}
$pSTAT2{:}\, pSTAT2_{nuc} + RIG_{gene\_off} \rightarrow RIG_{gene\_on,STAT2\_2}$	$A_{RIG\_g,STAT2\_2}$	$A_{RIG\_g,STAT2\_2}$	10^{-6}
$pSTAT1{:}\, pSTAT2_{nuc} + RIG_{gene\_off} \leftarrow RIG_{gene\_on,STAT1\_2}$	D_{RIG_g}	D_{RIG_g}	10^{-2}
$pSTAT2{:}\, pSTAT2_{nuc} + RIG_{gene\_off} \leftarrow RIG_{gene\_on,STAT2\_2}$			
$pSTAT1{:}\, pSTAT2_{nuc} + SOCS1_{gene\_off} \rightarrow SOCS1_{gene\_on,STAT1\_2}$	$A_{SOCS1\_g,STAT1\_2}$	$A_{SOCS1\_g,STAT1\_2}$	3×10^{-6}
$pSTAT2{:}\, pSTAT2_{nuc} + SOCS1_{gene\_off} \rightarrow SOCS1_{gene\_on,STAT2\_2}$	$A_{SOCS1\_g,STAT2\_2}$	$A_{SOCS1\_g,STAT2\_2}$	3×10^{-6}

$pSTAT1{:}pSTAT2_{nuc} + SOCS1_{gene\_off} \leftarrow SOCS1_{gene\_on,STAT1\_2}$	D_{SOCS1_g}	D_{SOCS1_g}	10^{-2}
$pSTAT2{:}pSTAT2_{nuc} + SOCS1_{gene\_off} \leftarrow SOCS1_{gene\_on,STAT2\_2}$			
$NFkB_{nuc} + TNFa_{gene\_off} \rightarrow TNFa_{gene\_off,NFkB\times 1}$	$4 \cdot A_{TNFa\_g,NFkB}$	$A_{TNFa\_g,NFkB}$	3×10^{-7}
$NFkB_{nuc} + TNFa_{gene\_off,NFkB\_1} \rightarrow TNFa_{gene\_off,NFkB\times 2}$	$3 \cdot A_{TNFa\_g,NFkB}$		
$NFkB_{nuc} + TNFa_{gene\_off,NFkB\_2} \rightarrow TNFa_{gene\_off,NFkB\times 3}$	$2 \cdot A_{TNFa\_g,NFkB}$		
$NFkB_{nuc} + TNFa_{gene\_off,NFkB\_3} \rightarrow TNFa_{gene\_on,NFkB\times 4}$	$A_{TNFa\_g,NFkB}$		
$NFkB_{nuc} + TNFa_{gene\_off} \leftarrow TNFa_{gene\_off,NFkB\times 1}$	D_{TNFa_g}	D_{TNFa_g}	10^{-2}
$NFkB_{nuc} + TNFa_{gene\_off,NFkB\times 1} \leftarrow TNFa_{gene\_off,NFkB\times 2}$	$2 \cdot D_{TNFa\_g}$		
$NFkB_{nuc} + TNFa_{gene\_off,NFkB\times 2} \leftarrow TNFa_{gene\_off,NFkB\times 3}$	$3 \cdot D_{TNFa\_g}$		
$NFkB_{nuc} + TNFa_{gene\_off,NFkB\times 3} \leftarrow TNFa_{gene\_on,NFkB\times 4}$	$4 \cdot D_{TNFa\_g}$		
$NFkB_{nuc} + IL6_{gene\_off} \rightarrow IL6_{gene\_off,NFkB\times 1}$	$4 \cdot A_{IL6\_g,NFkB}$	$A_{IL6\_g,NFkB}$	3×10^{-7}
$NFkB_{nuc} + IL6_{gene\_off,NFkB\times 1} \rightarrow IL6_{gene\_off,NFkB\times 2}$	$3 \cdot A_{IL6\_g,NFkB}$		
$NFkB_{nuc} + IL6_{gene\_off,NFkB\times 2} \rightarrow IL6_{gene\_off,NFkB\times 3}$	$2 \cdot A_{IL6\_g,NFkB}$		
$NFkB_{nuc} + IL6_{gene\_off,NFkB\times 3} \rightarrow IL6_{gene\_on,NFkB\times 4}$	$A_{IL6\_g,NFkB}$		
$NFkB_{nuc} + IL6_{gene\_off} \leftarrow IL6_{gene\_off,NFkB\times 1}$	D_{IL6_g}	D_{IL6_g}	10^{-2}
$NFkB_{nuc} + IL6_{gene\_off,NFkB\times 1} \leftarrow IL6_{gene\_off,NFkB\times 2}$	$2 \cdot D_{IL6\_g}$		
$NFkB_{nuc} + IL6_{gene\_off,NFkB\times 2} \leftarrow IL6_{gene\_off,NFkB\times 3}$	$3 \cdot D_{IL6\_g}$		
$NFkB_{nuc} + IL6_{gene\_off,NFkB\times 3} \leftarrow IL6_{gene\_on,NFkB\times 4}$	$4 \cdot D_{IL6\_g}$		
$pIRF3{:}pIRF3_{nuc} + RANTES_{gene\_off} \rightarrow RANTES_{gene\_on,IRF3}$	$A_{RANTES\_g,IRF3}$	$A_{RANTES\_g,IRF3}$	2×10^{-7}
$pIRF3{:}pIRF3_{nuc} + RANTES_{gene\_off} \leftarrow RANTES_{gene\_on,IRF3}$	D_{RANTES_g}	D_{RANTES_g}	10^{-2}

$NFkB_{nuc} + CCL2_{gene\_off} \rightarrow CCL2_{gene\_off,NFkB\times1}$	$2 \cdot A_{CCL2\_g,NFkB}$	$A_{CCL2\_g,NFkB}$	5×10^{-7}
$NFkB_{nuc} + CCL2_{gene_{on},Stat1\_2} \rightarrow CCL2_{gene\_on,Stat1\_2,NFkB\times1}$			
$NFkB_{nuc} + CCL2_{gene_{on},Stat2\_2} \rightarrow CCL2_{gene\_on,Stat2\_2,NFkB\times1}$			
$NFkB_{nuc} + CCL2_{gene\_off,NFkB\times1} \rightarrow CCL2_{gene\_on,NFkB\times2}$	$A_{CCL2\_g,NFkB}$		
$NFkB_{nuc} + CCL2_{gene\_on,Stat1\_2,NFkB\times1} \rightarrow CCL2_{gene\_on,Stat1\_2,NFkB\times2}$			
$NFkB_{nuc} + CCL2_{gene\_on,Stat2\_2,NFkB\times1} \rightarrow CCL2_{gene\_on,Stat2\_2,NFkB\times2}$			
$NFkB_{nuc} + CCL2_{gene\_off} \leftarrow CCL2_{gene\_off,NFkB\times1}$	D_{CCL2_g}	D_{CCL2_g}	10^{-2}
$NFkB_{nuc} + CCL2_{gene_{on},Stat1\_2} \leftarrow CCL2_{gene\_on,Stat1\_2,NFkB\times1}$			
$NFkB_{nuc} + CCL2_{gene_{on},Stat2\_2} \leftarrow CCL2_{gene\_on,Stat2\_2,NFkB\times1}$			
$NFkB_{nuc} + CCL2_{gene\_off,NFkB\times1} \leftarrow CCL2_{gene\_on,NFkB\times2}$	$2 \cdot D_{CCL2\_g}$		
$NFkB_{nuc} + CCL2_{gene\_on,Stat1\_2,NFkB\times1} \leftarrow CCL2_{gene\_on,Stat1\_2,NFkB\times2}$			
$NFkB_{nuc} + CCL2_{gene\_on,Stat2\_2,NFkB\times1} \leftarrow CCL2_{gene\_on,Stat2\_2,NFkB\times2}$			
$pSTAT1{:}\,pSTAT2_{nuc} + CCL2_{gene\_off} \rightarrow CCL2_{gene\_on,STAT1\_2}$	$A_{CCL2\_g,STAT1\_2}$	$A_{CCL2\_g,STAT1\_2}$	10^{-7}
$pSTAT1{:}\,pSTAT2_{nuc} + CCL2_{gene\_off,NFkB\times1} \rightarrow CCL2_{gene\_on,STAT1\_2,NFkB\times1}$			
$pSTAT1{:}\,pSTAT2_{nuc} + CCL2_{gene\_on,NFkB\times2} \rightarrow CCL2_{gene\_on,STAT1\_2,NFkB\times2}$			
$pSTAT2{:}\,pSTAT2_{nuc} + CCL2_{gene\_off} \rightarrow CCL2_{gene\_on,STAT2\_2}$	$A_{CCL2\_g,STAT2\_2}$	$A_{CCL2\_g,STAT2\_2}$	10^{-7}
$pSTAT2{:}\,pSTAT2_{nuc} + CCL2_{gene\_off,NFkB\times1} \rightarrow CCL2_{gene\_on,STAT2\_2,NFkB\times1}$			
$pSTAT2{:}\,pSTAT2_{nuc} + CCL2_{gene\_on,NFkB\times2} \rightarrow CCL2_{gene\_on,STAT2\_2,NFkB\times2}$			
$pSTAT1{:}\,pSTAT2_{nuc} + CCL2_{gene\_off} \leftarrow CCL2_{gene\_on,STAT1\_2}$	D_{CCL2_g}	D_{CCL2_g}	10^{-2}
$pSTAT1{:}\,pSTAT2_{nuc} + CCL2_{gene\_off,NFkB\times1} \leftarrow CCL2_{gene\_on,STAT1\_2,NFkB\times1}$			
$pSTAT1{:}\,pSTAT2_{nuc} + CCL2_{gene\_on,NFkB\times2} \leftarrow CCL2_{gene\_on,STAT1\_2,NFkB\times2}$			
$pSTAT2{:}\,pSTAT2_{nuc} + CCL2_{gene\_off} \leftarrow CCL2_{gene\_on,STAT2\_2}$			
$pSTAT2{:}\,pSTAT2_{nuc} + CCL2_{gene\_off,NFkB\times1} \leftarrow CCL2_{gene\_on,STAT2\_2,NFkB\times1}$			
$pSTAT2{:}\,pSTAT2_{nuc} + CCL2_{gene\_on,NFkB\times2} \leftarrow CCL2_{gene\_on,STAT2\_2,NFkB\times2}$			

$pSTAT1{:}pSTAT2_{nuc} + ISG56_{gene\_off} \rightarrow ISG56_{gene\_on,STAT1\_2}$	$A_{ISG56\_g,STAT1\_2}$	$A_{ISG56\_g,STAT1\_2}$	3×10^{-7}
$pSTAT2{:}pSTAT2_{nuc} + ISG56_{gene\_off} \rightarrow ISG56_{gene\_on,STAT2\_2}$	$A_{ISG56\_g,STAT2\_2}$	$A_{ISG56\_g,STAT2\_2}$	3×10^{-7}
$pSTAT1{:}pSTAT2_{nuc} + ISG56_{gene\_off} \leftarrow ISG56_{gene\_on,STAT1\_2}$	D_{ISG56_g}	D_{ISG56_g}	10^{-2}
$pSTAT2{:}pSTAT2_{nuc} + ISG56_{gene\_off} \leftarrow ISG56_{gene\_on,STAT2\_2}$			
$ISG54_{gene\_off} \rightarrow ISG54_{gene\_on}$	A_{ISG54_g}	A_{ISG54_g}	10^{-4}
$pSTAT1{:}pSTAT2_{nuc} + ISG54_{gene\_off} \rightarrow ISG54_{gene\_on,STAT1\_2}$	$A_{ISG54\_g,STAT1\_2}$	$A_{ISG54\_g,STAT1\_2}$	3×10^{-7}
$pSTAT2{:}pSTAT2_{nuc} + ISG54_{gene\_off} \rightarrow ISG54_{gene\_on,STAT2\_2}$	$A_{ISG54\_g,STAT2\_2}$	$A_{ISG54\_g,STAT2\_2}$	3×10^{-7}
$ISG54_{gene\_off} \leftarrow ISG54_{gene\_on}$	D_{ISG54_g}	D_{ISG54_g}	10^{-2}
$pSTAT1{:}pSTAT2_{nuc} + ISG54_{gene\_off} \leftarrow ISG54_{gene\_on,STAT1\_2}$			
$pSTAT2{:}pSTAT2_{nuc} + ISG54_{gene\_off} \leftarrow ISG54_{gene\_on,STAT2\_2}$			
$IRF1_{gene\_off} \rightarrow IRF1_{gene\_on}$	A_{IRF1_g}	A_{IRF1_g}	3×10^{-4}
$pSTAT1{:}pSTAT2_{nuc} + IRF1_{gene\_off} \rightarrow IRF1_{gene\_on,STAT1\_2}$	$A_{IRF1\_g,STAT1\_2}$	$A_{IRF1\_g,STAT1\_2}$	3×10^{-7}
$pSTAT2{:}pSTAT2_{nuc} + IRF1_{gene\_off} \rightarrow IRF1_{gene\_on,STAT2\_2}$	$A_{IRF1\_g,STAT2\_2}$	$A_{IRF1\_g,STAT2\_2}$	3×10^{-7}
$IRF1_{gene\_off} \leftarrow IRF1_{gene\_on}$	D_{IRF1_g}	D_{IRF1_g}	10^{-2}
$pSTAT1{:}pSTAT2_{nuc} + IRF1_{gene\_off} \leftarrow IRF1_{gene\_on,STAT1\_2}$			
$pSTAT2{:}pSTAT2_{nuc} + IRF1_{gene\_off} \leftarrow IRF1_{gene\_on,STAT2\_2}$			
$pSTAT1{:}pSTAT2_{nuc} + IRF5_{gene\_off} \rightarrow IRF5_{gene\_on,STAT1\_2}$	$A_{IRF5\_g,STAT1\_2}$	$A_{IRF5\_g,STAT1\_2}$	4×10^{-7}
$pSTAT2{:}pSTAT2_{nuc} + IRF5_{gene\_off} \rightarrow IRF5_{gene\_on,STAT2\_2}$	$A_{IRF5\_g,STAT2\_2}$	$A_{IRF5\_g,STAT2\_2}$	4×10^{-7}
$pSTAT1{:}pSTAT2_{nuc} + IRF5_{gene\_off} \leftarrow IRF5_{gene\_on,STAT1\_2}$	D_{IRF5_g}	D_{IRF5_g}	10^{-2}
$pSTAT2{:}pSTAT2_{nuc} + IRF5_{gene\_off} \leftarrow IRF5_{gene\_on,STAT2\_2}$			
$pSTAT1{:}pSTAT2_{nuc} + ISG15_{gene\_off} \rightarrow ISG15_{gene\_on,STAT1\_2}$	$A_{ISG15\_g,STAT1\_2}$	$A_{ISG15\_g,STAT1\_2}$	4×10^{-7}
$pSTAT2{:}pSTAT2_{nuc} + ISG15_{gene\_off} \rightarrow ISG15_{gene\_on,STAT2\_2}$	$A_{ISG15\_g,STAT2\_2}$	$A_{ISG15\_g,STAT2\_2}$	4×10^{-7}
$pSTAT1{:}pSTAT2_{nuc} + ISG15_{gene\_off} \leftarrow ISG15_{gene\_on,STAT1\_2}$	D_{ISG15_g}	D_{ISG15_g}	10^{-2}
$pSTAT2{:}pSTAT2_{nuc} + ISG15_{gene\_off} \leftarrow ISG15_{gene\_on,STAT2\_2}$			

mRNA transcription			
$PKR_{gene\_on,STAT1\_2} \rightarrow PKR_{gene\_on,STAT1\_2} + PKR_{mRNA}$	T_{PKR}	T_{PKR}	1.5×10^{-2}
$PKR_{gene\_on,STAT2\_2} \rightarrow PKR_{gene\_on,STAT2\_2} + PKR_{mRNA}$			
$OAS1A_{gene\_on,STAT1\_2} \rightarrow OAS1A_{gene\_on,STAT1\_2} + OAS1A_{mRNA}$	T_{OAS1A}	T_{OAS1A}	5×10^{-3}
$OAS1A_{gene\_on,STAT2\_2} \rightarrow OAS1A_{gene\_on,STAT2\_2} + OAS1A_{mRNA}$			
$IRF7_{gene\_on,STAT1\_2} \rightarrow IRF7_{gene\_on,STAT1\_2} + IRF7_{mRNA}$	T_{IRF7}	T_{IRF7}	3×10^{-2}
$IRF7_{gene\_on,STAT2\_2} \rightarrow IRF7_{gene\_on,STAT2\_2} + IRF7_{mRNA}$			
$STAT1_{gene\_on} \rightarrow STAT1_{gene\_on} + STAT1_{mRNA}$	T_{STAT1}	T_{STAT1}	4×10^{-2}
$STAT1_{gene\_on,STAT1\_2} \rightarrow STAT1_{gene\_on,STAT1\_2} + STAT1_{mRNA}$			
$STAT1_{gene\_on,STAT2\_2} \rightarrow STAT1_{gene\_on,STAT2\_2} + STAT1_{mRNA}$			
$STAT2_{gene\_on} \rightarrow STAT2_{gene\_on} + STAT2_{mRNA}$	T_{STAT2}	T_{STAT2}	4×10^{-2}
$STAT2_{gene\_on,STAT1\_2} \rightarrow STAT2_{gene\_on,STAT1\_2} + STAT2_{mRNA}$			
$STAT2_{gene\_on,STAT2\_2} \rightarrow STAT2_{gene\_on,STAT2\_2} + STAT2_{mRNA}$			
$RIG_{gene\_on,STAT1\_2} \rightarrow RIG_{gene\_on,STAT1\_2} + RIG_{mRNA}$	T_{RIG}	T_{RIG}	10^{-2}
$RIG_{gene\_on,STAT2\_2} \rightarrow RIG_{gene\_on,STAT2\_2} + RIG_{mRNA}$			
$SOCS1_{gene\_on,STAT1\_2} \rightarrow SOCS1_{gene\_on,STAT1\_2} + SOCS1_{mRNA}$	T_{SOCS1}	T_{SOCS1}	3×10^{-3}
$SOCS1_{gene\_on,STAT2\_2} \rightarrow SOCS1_{gene\_on,STAT2\_2} + SOCS1_{mRNA}$			
$TNFa_{gene\_on,NF\kappa B\times 4} \rightarrow TNFa_{gene\_on,NF\kappa B\times 4} + TNFa_{mRNA}$	T_{TNFa}	T_{TNFa}	3×10^{-2}
$IL6_{gene\_on,NF\kappa B\times 4} \rightarrow IL6_{gene\_on,NF\kappa B\times 4} + IL6_{mRNA}$	T_{IL6}	T_{IL6}	2×10^{-2}
$RANTES_{gene\_on,IRF3} \rightarrow RANTES_{gene\_on,IRF3} + RANTES_{mRNA}$	T_{RANTES}	T_{RANTES}	4×10^{-3}

$CCL2_{gene\_on,NF\kappa B\times 2} \rightarrow CCL2_{gene\_on,NF\kappa B\times 2} + CCL2_{mRNA}$			
$CCL2_{gene\_on,STAT1\_2} \rightarrow CCL2_{gene\_on,STAT1\_2} + CCL2_{mRNA}$			
$CCL2_{gene\_on,STAT2\_2} \rightarrow CCL2_{gene\_on,STAT2\_2} + CCL2_{mRNA}$			
$CCL2_{gene\_on,STAT1\_2,NF\kappa B\times 1} \rightarrow CCL2_{gene\_on,STAT1\_2,NF\kappa B\times 1} + CCL2_{mRNA}$	T_{CCL2}	T_{CCL2}	2×10^{-2}
$CCL2_{gene\_on,STAT2\_2,NF\kappa B\times 1} \rightarrow CCL2_{gene\_on,STAT2\_2,NF\kappa B\times 1} + CCL2_{mRNA}$			
$CCL2_{gene\_on,STAT1\_2,NF\kappa B\times 2} \rightarrow CCL2_{gene\_on,STAT1\_2,NF\kappa B\times 2} + CCL2_{mRNA}$			
$CCL2_{gene\_on,STAT2\_2,NF\kappa B\times 2} \rightarrow CCL2_{gene\_on,STAT2\_2,NF\kappa B\times 2} + CCL2_{mRNA}$			
$ISG54_{gene\_on} \rightarrow ISG54_{gene\_on} + ISG54_{mRNA}$			
$ISG54_{gene\_on,STAT1\_2} \rightarrow ISG54_{gene\_on,STAT1\_2} + ISG54_{mRNA}$	T_{ISG54}	T_{ISG54}	3×10^{-2}
$ISG54_{gene\_on,STAT2\_2} \rightarrow ISG54_{gene\_on,STAT2\_2} + ISG54_{mRNA}$			
$ISG56_{gene\_on,STAT1\_2} \rightarrow ISG56_{gene\_on,STAT1\_2} + ISG56_{mRNA}$	T_{ISG56}	T_{ISG56}	3×10^{-2}
$ISG56_{gene\_on,STAT2\_2} \rightarrow ISG56_{gene\_on,STAT2\_2} + ISG56_{mRNA}$			
$IRF1_{gene\_on} \rightarrow IRF1_{gene\_on} + IRF1_{mRNA}$			
$IRF1_{gene\_on,STAT1\_2} \rightarrow IRF1_{gene\_on,STAT1\_2} + IRF1_{mRNA}$	T_{IRF1}	T_{IRF1}	5×10^{-2}
$IRF1_{gene\_on,STAT2\_2} \rightarrow IRF1_{gene\_on,STAT2\_2} + IRF1_{mRNA}$			
$IRF5_{gene\_on,STAT1\_2} \rightarrow IRF5_{gene\_on,STAT1\_2} + IRF5_{mRNA}$	T_{IRF5}	T_{IRF5}	2×10^{-3}
$IRF5_{gene\_on,STAT2\_2} \rightarrow IRF5_{gene\_on,STAT2\_2} + IRF5_{mRNA}$			
$ISG15_{gene\_on,STAT1\_2} \rightarrow ISG15_{gene\_on,STAT1\_2} + ISG15_{mRNA}$	T_{ISG15}	T_{ISG15}	4×10^{-2}
$ISG15_{gene\_on,STAT2\_2} \rightarrow ISG15_{gene\_on,STAT2\_2} + ISG15_{mRNA}$			
mRNA degradation			
$PKR_{mRNA} \rightarrow \emptyset$	L_{PKR_mRNA}	L_{PKR_mRNA}	10^{-4}
$OAS1A_{mRNA} \rightarrow \emptyset$	L_{OAS1A_mRNA}	L_{OAS1A_mRNA}	10^{-4}
$IRF7_{mRNA} \rightarrow \emptyset$	L_{IRF7_mRNA}	L_{IRF7_mRNA}	10^{-4}
$RIG_{mRNA} \rightarrow \emptyset$	L_{RIG_mRNA}	L_{RIG_mRNA}	10^{-4}
$STAT1_{mRNA} \rightarrow \emptyset$	L_{STAT1_mRNA}	L_{STAT1_mRNA}	10^{-4}

$STAT2_{mRNA} \rightarrow \emptyset$	L_{STAT2_mRNA}	L_{STAT2_mRNA}	10^{-4}
$SOCS1_{mRNA} \rightarrow \emptyset$	L_{SOCS1_mRNA}	L_{SOCS1_mRNA}	10^{-4}
$TNFa_{mRNA} \rightarrow \emptyset$	L_{TNFa_mRNA}	L_{TNFa_mRNA}	10^{-3}
$IL6_{mRNA} \rightarrow \emptyset$	L_{IL6_mRNA}	L_{IL6_mRNA}	5×10^{-4}
$RANTES_{mRNA} \rightarrow \emptyset$	L_{RANTES_mRNA}	L_{RANTES_mRNA}	10^{-4}
$CCL2_{mRNA} \rightarrow \emptyset$	L_{CCL2_mRNA}	L_{CCL2_mRNA}	10^{-4}
$ISG54_{mRNA} \rightarrow \emptyset$	L_{ISG54_mRNA}	L_{ISG54_mRNA}	10^{-4}
$ISG56_{mRNA} \rightarrow \emptyset$	L_{ISG56_mRNA}	L_{ISG56_mRNA}	10^{-4}
$IRF1_{mRNA} \rightarrow \emptyset$	L_{IRF1_mRNA}	L_{IRF1_mRNA}	2×10^{-4}
$IRF5_{mRNA} \rightarrow \emptyset$	L_{IRF5_mRNA}	L_{IRF5_mRNA}	10^{-4}
$ISG15_{mRNA} \rightarrow \emptyset$	L_{ISG15_mRNA}	L_{ISG15_mRNA}	10^{-4}
Protein synthesis			
$PKR_{mRNA} \rightarrow PKR_{mRNA} + PKR_{inactive}$	S_{PKR}	S_{PKR}	3×10^{-2}
$OAS1A_{mRNA} \rightarrow OAS1A_{mRNA} + OAS1A_{inactive}$	S_{OAS1A}	S_{OAS1A}	3×10^{-2}
$RIG_{mRNA} \rightarrow RIG_{mRNA} + RIG$	S_{RIG}	S_{RIG}	10^{-1}
$STAT1_{mRNA} \rightarrow STAT1_{mRNA} + STAT1_{cyt}$	S_{STAT1}	S_{STAT1}	10^{-2}
$STAT2_{mRNA} \rightarrow STAT2_{mRNA} + STAT2_{cyt}$	S_{STAT2}	S_{STAT2}	10^{-2}
$SOCS1_{mRNA} \rightarrow SOCS1_{mRNA} + SOCS1$	S_{SOCS1}	S_{SOCS1}	3×10^{-2}
$\emptyset \rightarrow TLR4$	S_{TLR4}	S_{TLR4}	2×10^{-2}
Protein degradation			
$PKR_{inactive} \rightarrow \emptyset$	L_{PKR}	L_{PKR}	10^{-5}
$PKR_{active} \rightarrow \emptyset$	L_{PKR_active}	L_{PKR_active}	3×10^{-5}
$OAS1A_{inactive} \rightarrow \emptyset$	L_{OAS1A}	L_{OAS1A}	10^{-5}
$OAS1A_{active} \rightarrow \emptyset$	L_{OAS1A_active}	L_{OAS1A_active}	3×10^{-5}

$STAT1_{cyt} \rightarrow \emptyset$			
$STAT1_{nuc} \rightarrow \emptyset$			
$STAT2_{cyt} \rightarrow \emptyset$			
$STAT2_{nuc} \rightarrow \emptyset$			
$pSTAT1{:}\,pSTAT2_{cyt} \rightarrow \emptyset$	L_{STAT}	L_{STAT}	3×10^{-6}
$pSTAT1{:}\,pSTAT2_{nuc} \rightarrow \emptyset$			
$pSTAT2{:}\,pSTAT2_{cyt} \rightarrow \emptyset$			
$pSTAT2{:}\,pSTAT2_{nuc} \rightarrow \emptyset$			
$pSTAT1_{cyt} \rightarrow \emptyset$			
$pSTAT1_{nuc} \rightarrow \emptyset$			
$pSTAT2_{cyt} \rightarrow \emptyset$	L_{pSTAT}	L_{pSTAT}	10^{-4}
$pSTAT2_{nuc} \rightarrow \emptyset$			
$RIG \rightarrow \emptyset$	$L_{RIG\_inactive}$	$L_{RIG\_inactive}$	3×10^{-5}
$MAVS{:}\,RIG \rightarrow MAVS$			
$MAVS{:}\,RIG{:}\,PolyIC \rightarrow MAVS$			
$MAVS{:}\,RIG{:}\,PolyIC{:}\,TRAF3 \rightarrow MAVS + TRAF3$	L_{RIG_active}	L_{RIG_active}	3×10^{-4}
$MAVS{:}\,RIG{:}\,PolyIC{:}\,TRAF3{:}\,pTBK1 \rightarrow MAVS + TRAF3 + pTBK1$			
$IFNb_{ext} \rightarrow \emptyset$	L_{IFNb_ext}	L_{IFNb_ext}	2×10^{-5}
$SOCS1 \rightarrow \emptyset$	L_{SOCS1}	L_{SOCS1}	10^{-5}
$TLR4 \rightarrow \emptyset$	L_{TLR4}	L_{TLR4}	10^{-5}

2 mRNA levels: RT-PCR results *vs.* model predictions

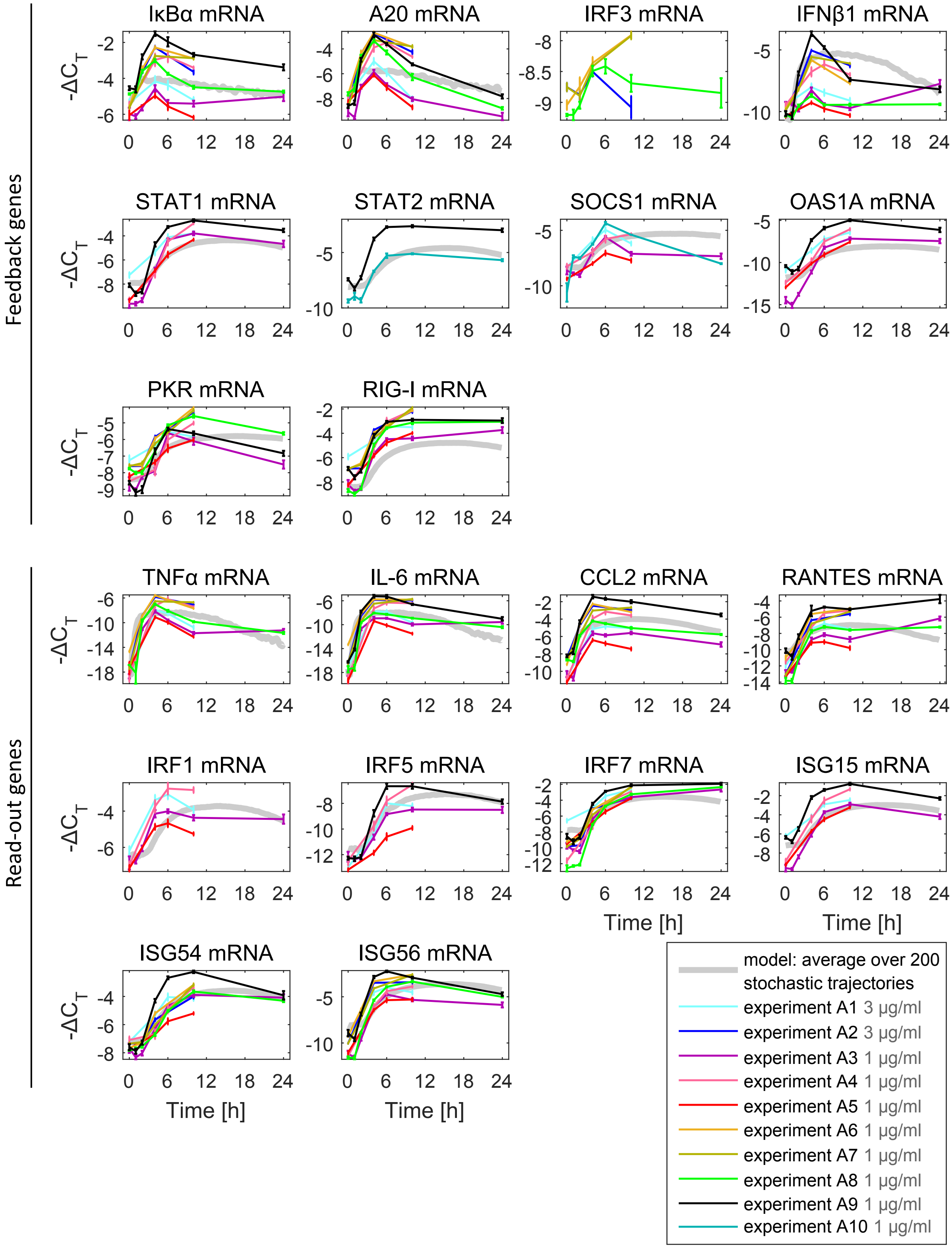

Figure A. MEF WT cells stimulated with poly(I:C) 1 μg/ml or 3 μg/ml as indicated in the key. Numerical simulations were performed for 1 μg/ml poly(I:C) dose.

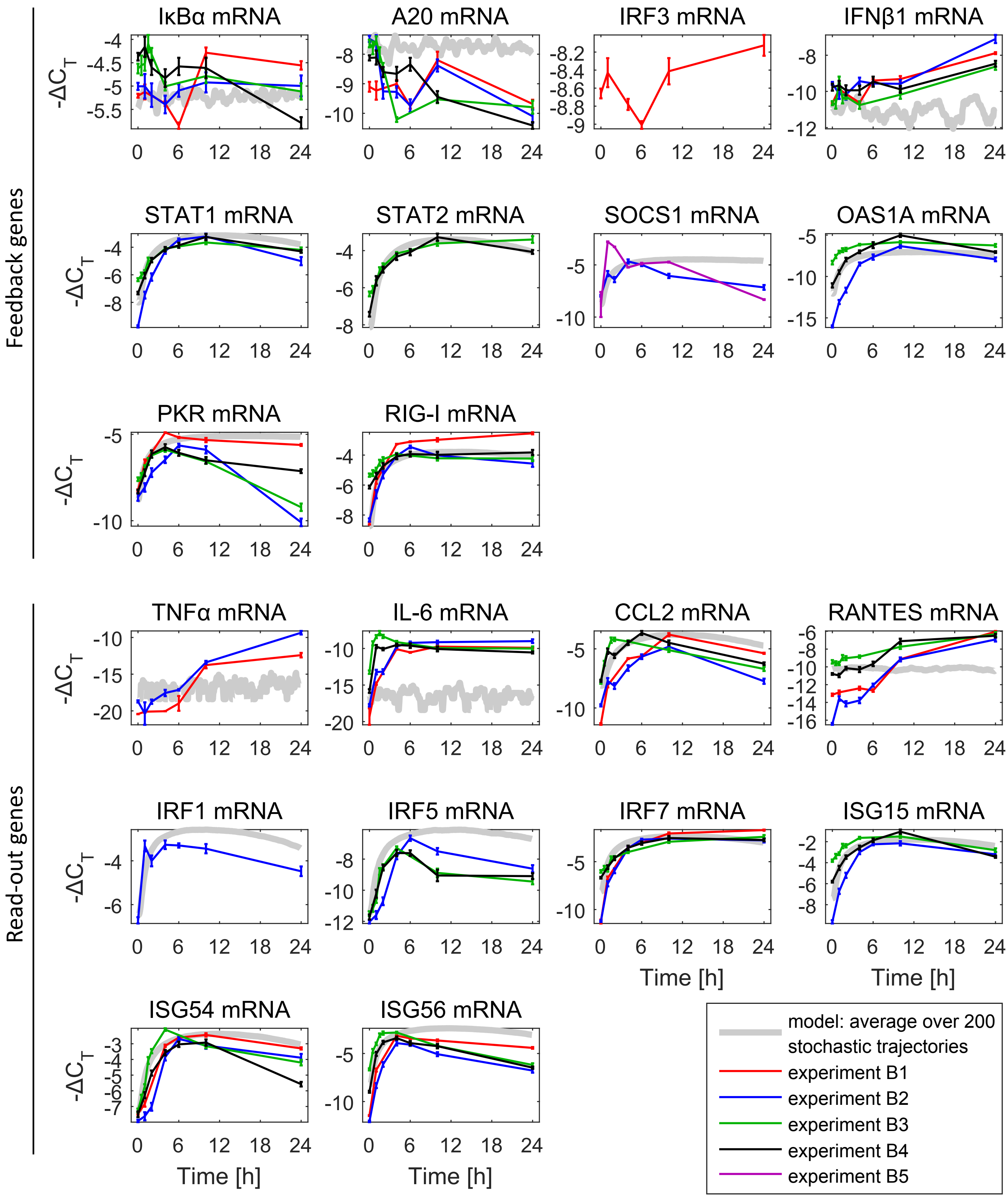

Figure B. MEF WT cells stimulated with IFNβ 1000 U/ml.

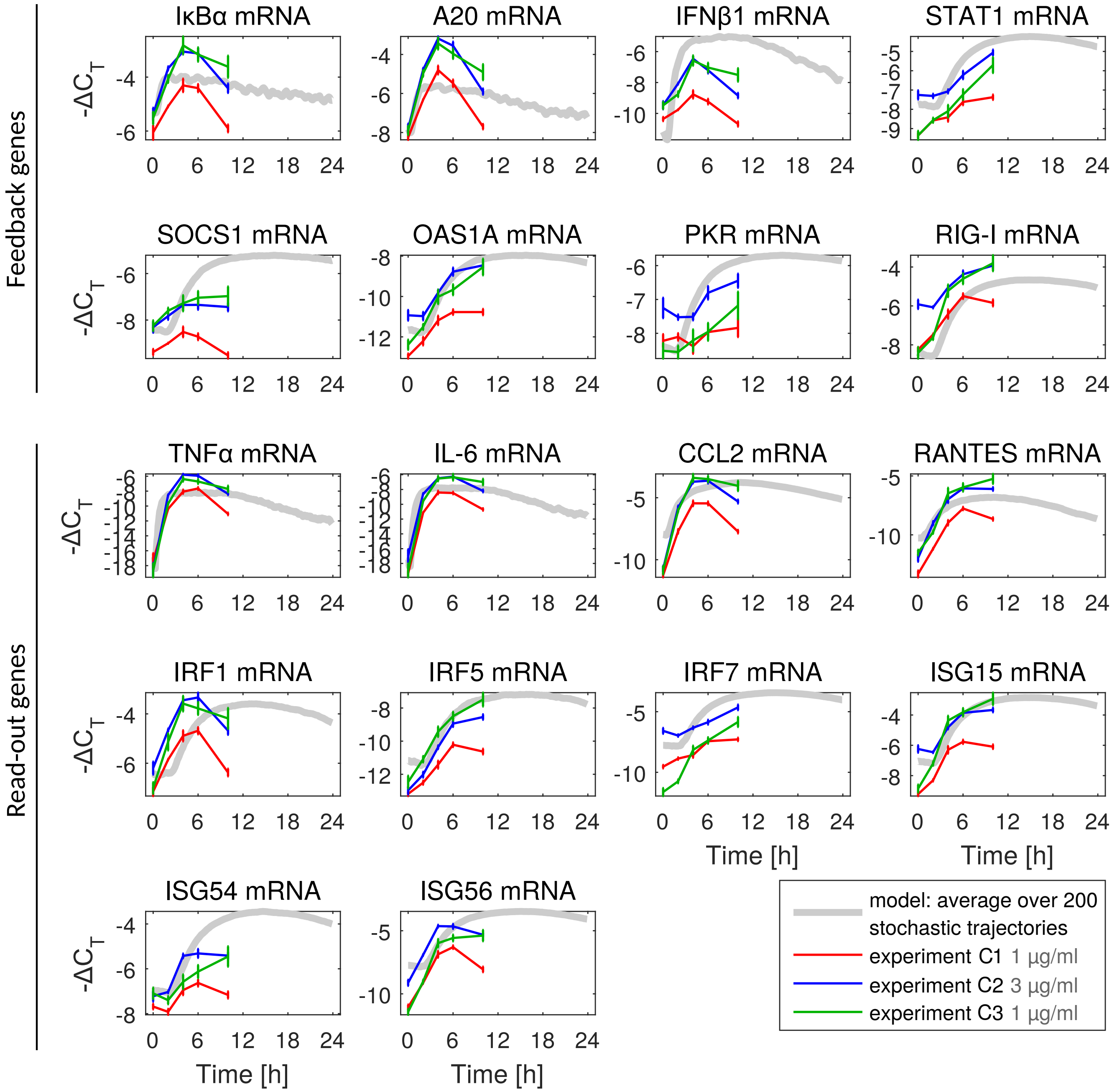

Figure C. MEF WT cells incubated with α-IFNAR 10 µg/ml and stimulated with poly(I:C) 1 µg/ml or 3 µg/ml (as indicated in the color key). Numerical simulations were performed for 1 µg/ml poly(I:C) dose.

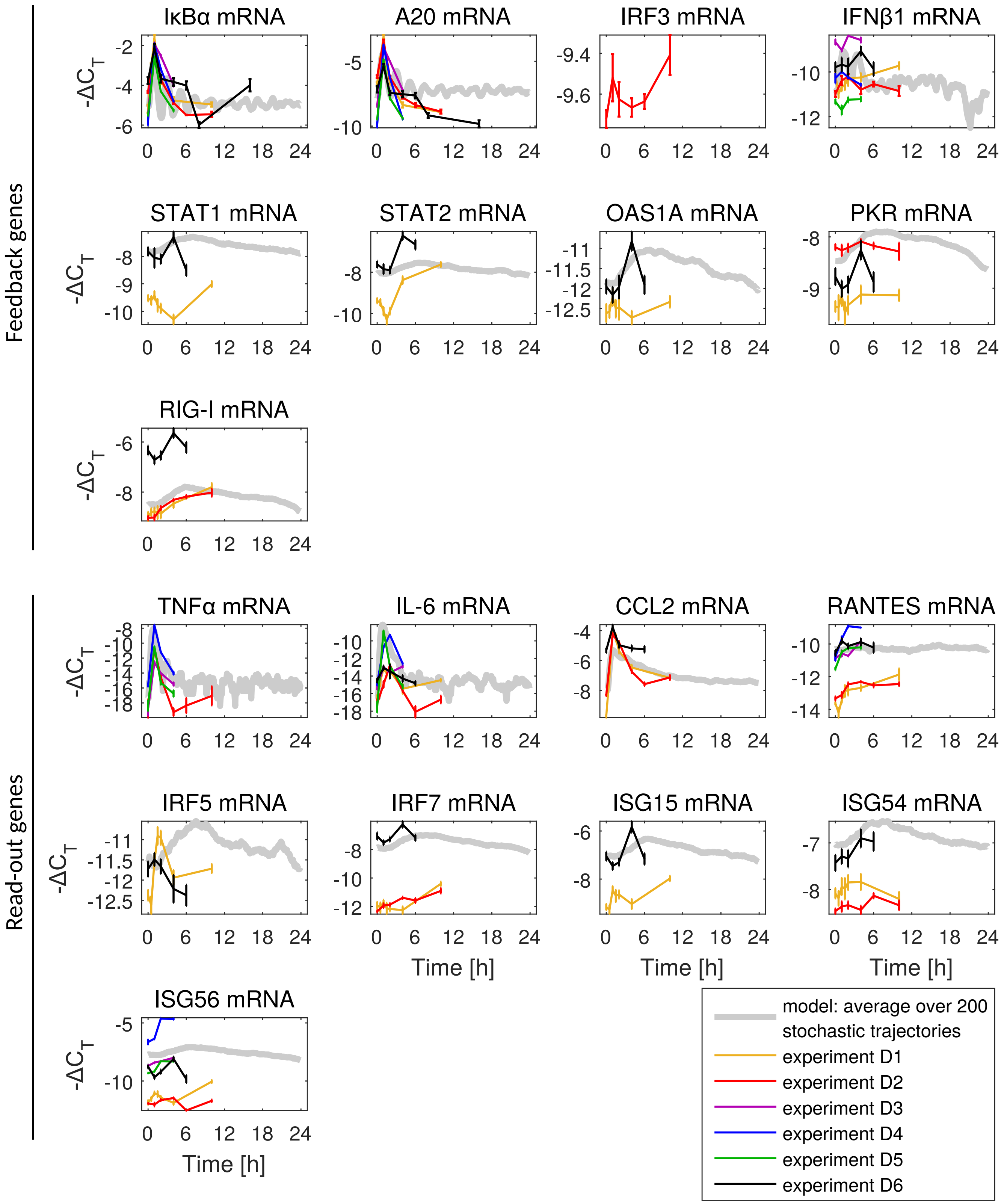

Figure D. MEF WT cells stimulated with LPS 1 μg/ml.

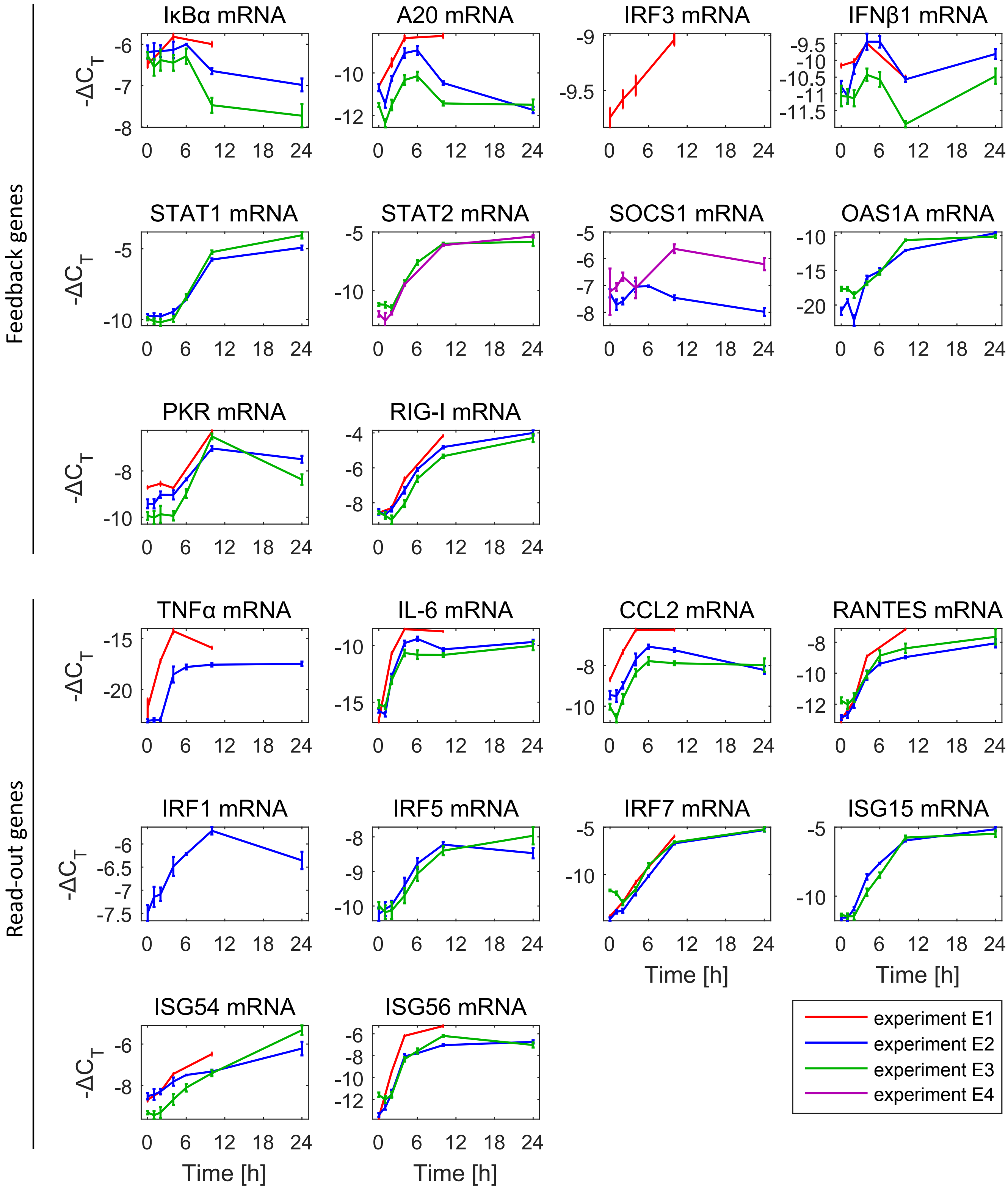

Figure E. MEF *RelA*$^{-/-}$ cells stimulated with poly(I:C) 1 μg/ml.

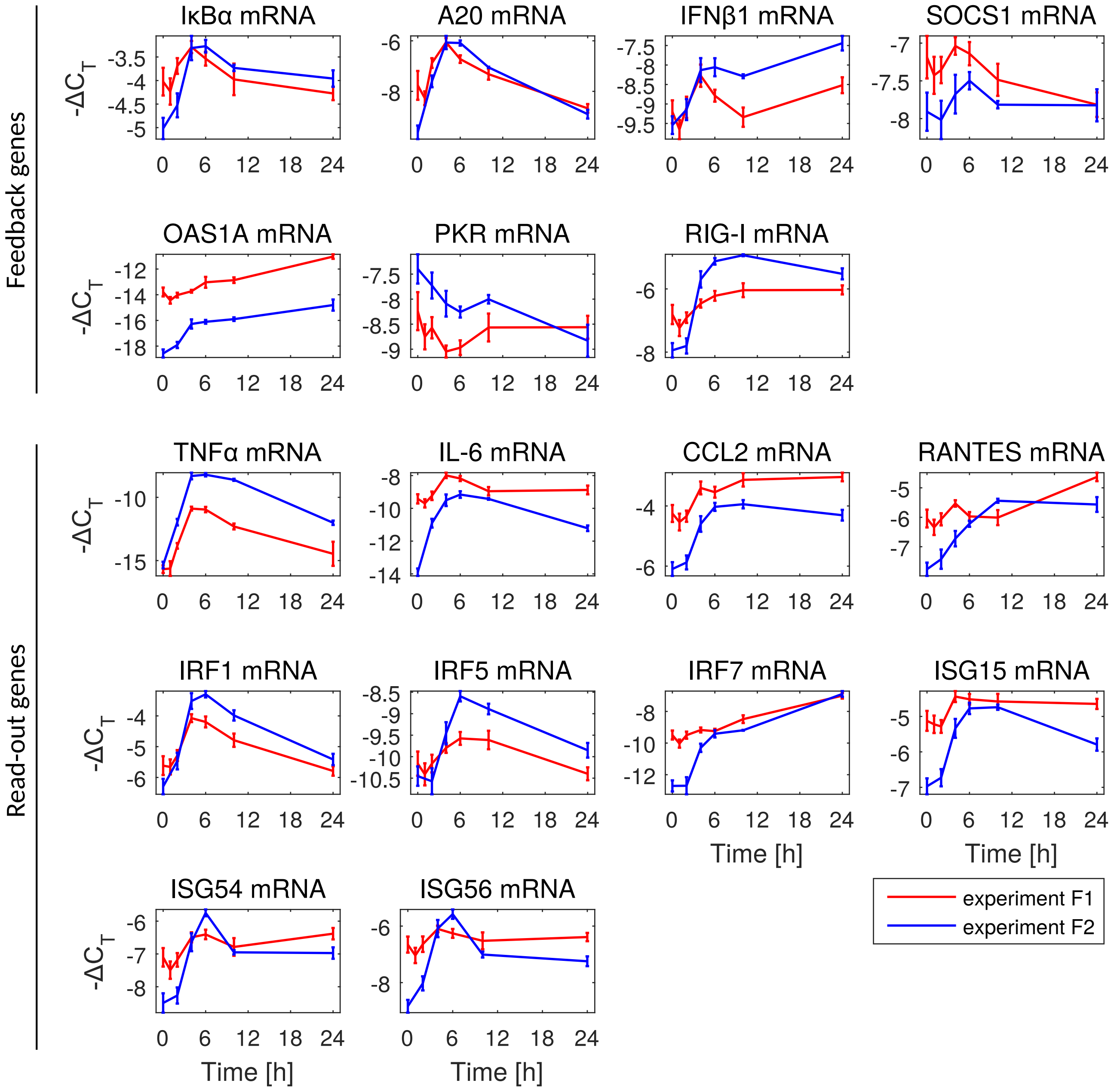

Figure F. MEF *Stat1*$^{-/-}$ cells stimulated with poly(I:C) 1 μg/ml.

Supplementary References

1. Faeder, J. R., Blinov, M. L. & Hlavacek, W. S. Rule-based modeling of biochemical systems with BioNetGen. *Methods Mol. Biol.* **500,** 113–167 (2009).
2. Nienałtowski, K., Włodarczyk, M., Lipniacki, T. & Komorowski, M. Clustering reveals limits of parameter identifiability in multi-parameter models of biochemical dynamics. *BMC Syst. Biol.* **9,** 65 (2015).
3. Tay, S. *et al.* Single-cell NF-κB dynamics reveal digital activation and analogue information processing. *Nature* **466,** 267–271 (2010).
4. Pękalski, J. *et al.* Spontaneous NF-κB activation by autocrine TNFα signaling: A computational analysis. *PLOS One* **8,** e78887 (2013).
5. Lin, R. *et al.* Negative regulation of the retinoic acid-inducible gene I-induced antiviral state by the ubiquitin-editing protein A20. *J. Biol. Chem.* **281,** 2095–2103 (2006).
6. Paz, S. *et al.* A functional C-terminal TRAF3-binding site in MAVS participates in positive and negative regulation of the IFN antiviral response. *Cell Res.* **21,** 895–910 (2011).
7. Gillespie, D. T. Exact stochastic simulation of coupled chemical reactions. *J. Phys. Chem.* **81,** 2340–61 (1977).
