# Supplementary material for: Cell fate in antiviral response arises in the crosstalk of IRF, NF-κB and JAK/STAT pathways
Source: Nat Commun. 2018 Feb 5;9:493. doi: 10.1038/s41467-017-02640-8 (PMC5799375; doi:10.1038/s41467-017-02640-8)
Supplement: Supplementary file 2 — Descriptions of Additional Supplementary Files [file 41467_2017_2640_MOESM2_ESM.pdf]

Descriptions of Additional Supplementary Files

File Name: Supplementary Dataset 1
Description: PowerPoint file with confocal microscopy images showing immunostaining of fixed MEF WT cells treated with 1 μg/ml poly(I:C) at 0, 2, 4, 6, 10, and 24 hr.

File Name: Supplementary Dataset 2
Description: PowerPoint file with confocal microscopy images showing immunostaining of fixed MEF WT cells treated with 1 μg/ml LPS at 0, 0.5, 1, 1.5, 2, 4, and 6 hr.

File Name: Supplementary Dataset 3
Description: PowerPoint file with confocal microscopy images showing immunostaining of fixed MEF WT cells treated with 1 μg/ml LPS, with or without CHX prestimulation, at 0, 0.5, 1, 1.5, 2, 4, and 6 hr.

File Name: Supplementary Dataset 4
Description: PowerPoint file with confocal microscopy images showing immunostaining of fixed MEF WT cells treated with 1 μg/ml poly(I:C), with either DMSO only or both DMSO and C16 prestimulation, at 0, 2, and 4 hr.

File Name: Supplementary Dataset 5
Description: PowerPoint file with confocal microscopy images showing immunostaining of fixed MEF WT cells treated with IFNβ 1000 U/ml at 0, 2, 4, 6, 10, and 24 hr.

File Name: Supplementary Dataset 6
Description: PowerPoint file with confocal microscopy images showing immunostaining of fixed MEF WT cells treated with 1 μg/ml poly(I:C) after 24 hr-long prestimulation with IFNβ, at 0, 2, 4, 6, 10, and 24 hr.

File Name: Supplementary Dataset 7
Description: PowerPoint file with confocal microscopy images showing immunostaining of fixed MEF Stat1–/– cells treated with 1 μg/ml poly(I:C) at 0, 2, 4, 6, 10, and 24 hr.

File Name: Supplementary Dataset 8
Description: PowerPoint file with confocal microscopy images showing immunostaining of fixed MEF Stat1–/– cells treated with 1 μg/ml poly(I:C), after 24-hr prestimulation with IFNβ 1000 U/ml, at 0, 2, 4, 6, 10, and 24 hr.

File Name: Supplementary Dataset 9
Description: PowerPoint file with confocal microscopy images showing immunostaining of fixed MEF WT cells treated with 0.1, 0.3, 1, and 3 μg/ml poly(I:C), all 4 hr after stimulation.

File Name: Supplementary Dataset 10
Description: PowerPoint file with confocal microscopy images showing immunostaining of fixed MEF RelA–/– cells treated with 1 μg/ml poly(I:C) at 0, 2, 4, 6, 10, and 24 hr.

File Name: Supplementary Dataset 11
Description: PowerPoint file with confocal microscopy images showing immunostaining of fixed WT and Tlr3–/– MEF cells purchased from OrientalBioService, treated with 1 μg/ml poly(I:C) at 0, 2, 4 hr.

File Name: Supplementary Dataset 12
Description: PDF file with agarose gel electrophoresis analysis of the poly(I:C) length.

File Name: Supplementary Dataset 13
Description: PDF file with a plot of GAPDH CT time profiles from all experiments.

File Name: Supplementary Dataset 14
Description: Excel file with fractions of apoptotic cells for all replicates – source data for Fig. 8 and Supplementary Fig. 9e.

File Name: Supplementary Dataset 15
Description: ZIP archive with mathematical model implementations in BIONETGEN language (BNGL), MATLAB, and SBML.

File Name: Supplementary Movie 1
Description: Video 1. Time-lapse confocal microscopy video showing nuclear translocation of GFP-tagged RelA (NF-κB; green) in cells with Hoechst-stained nuclei (blue) challenged by poly(I:C) (red). This video corresponds to Fig. 2c.

File Name: Supplementary Movie 2
Description: Video 2. Time-lapse confocal microscopy video showing nuclear translocation of GFP-tagged RelA (NF-κB; green) in cells with Hoechst 33342-stained nuclei (blue) challenged by poly(I:C). This video corresponds to Fig. 2c.

File Name: Supplementary Movie 3
Description: Video 3. Time-lapse confocal microscopy video showing nuclear translocation of GFP-tagged RelA (NF-κB; green) in cells with Hoechst-stained nuclei (blue) challenged by LPS.
This video corresponds to Fig. 2e.
