# Supplementary figures and images for: Cell fate in antiviral response arises in the crosstalk of IRF, NF-κB and JAK/STAT pathways
Source: Nat Commun. 2018 Feb 5;9:493. doi: 10.1038/s41467-017-02640-8 (PMC5799375; doi:10.1038/s41467-017-02640-8)

## Slide 1
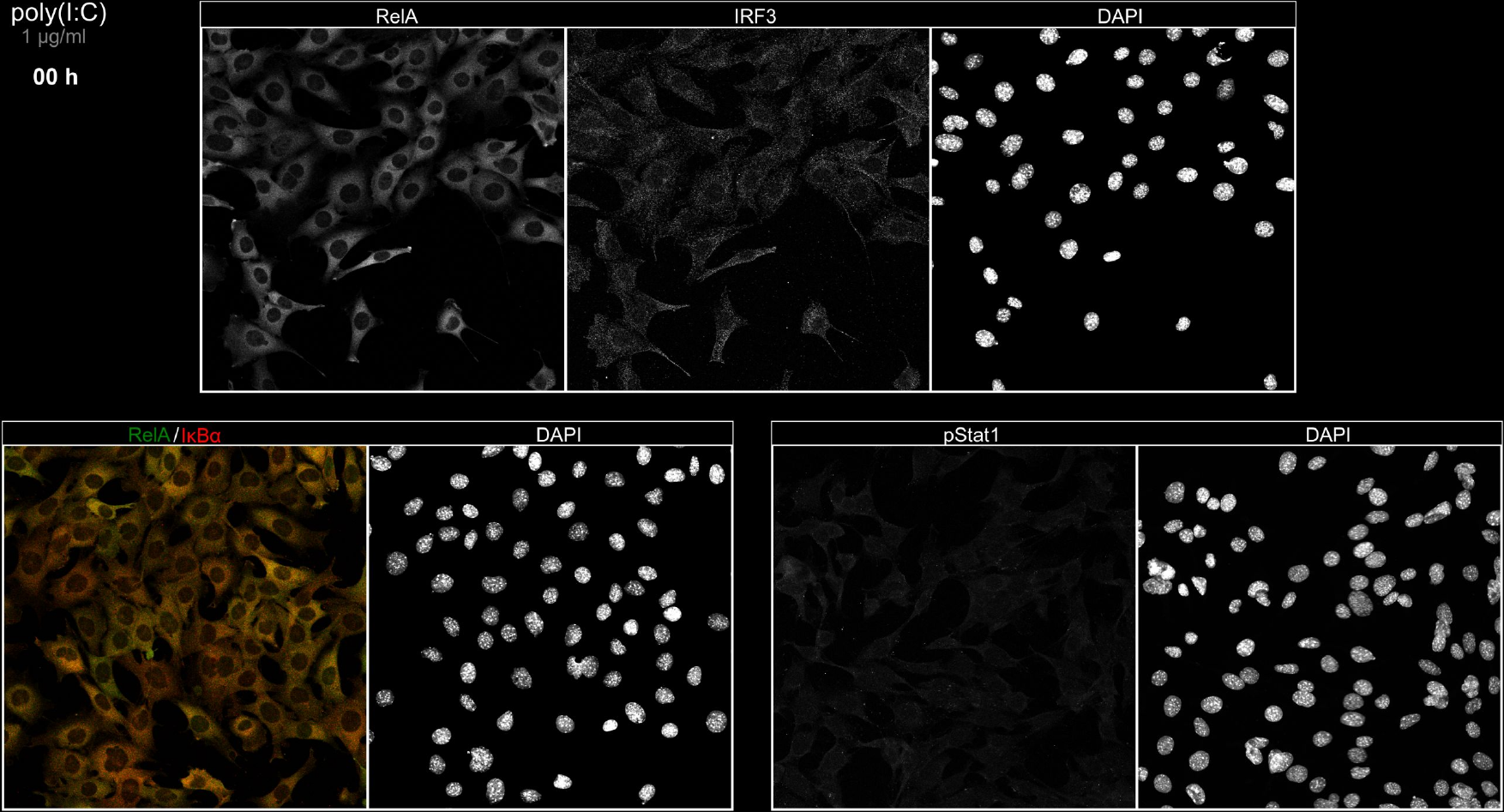

## Slide 2
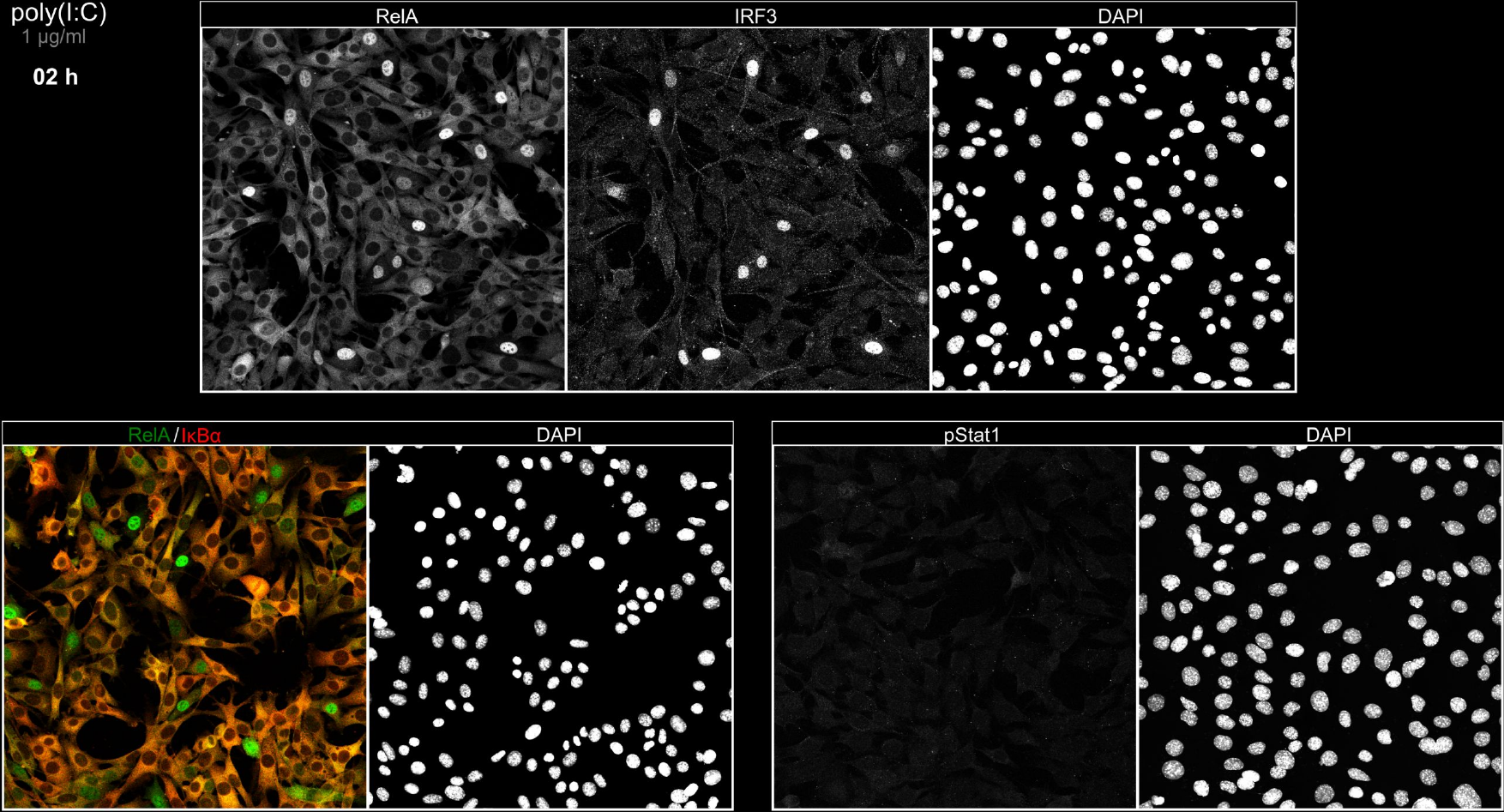

## Slide 3
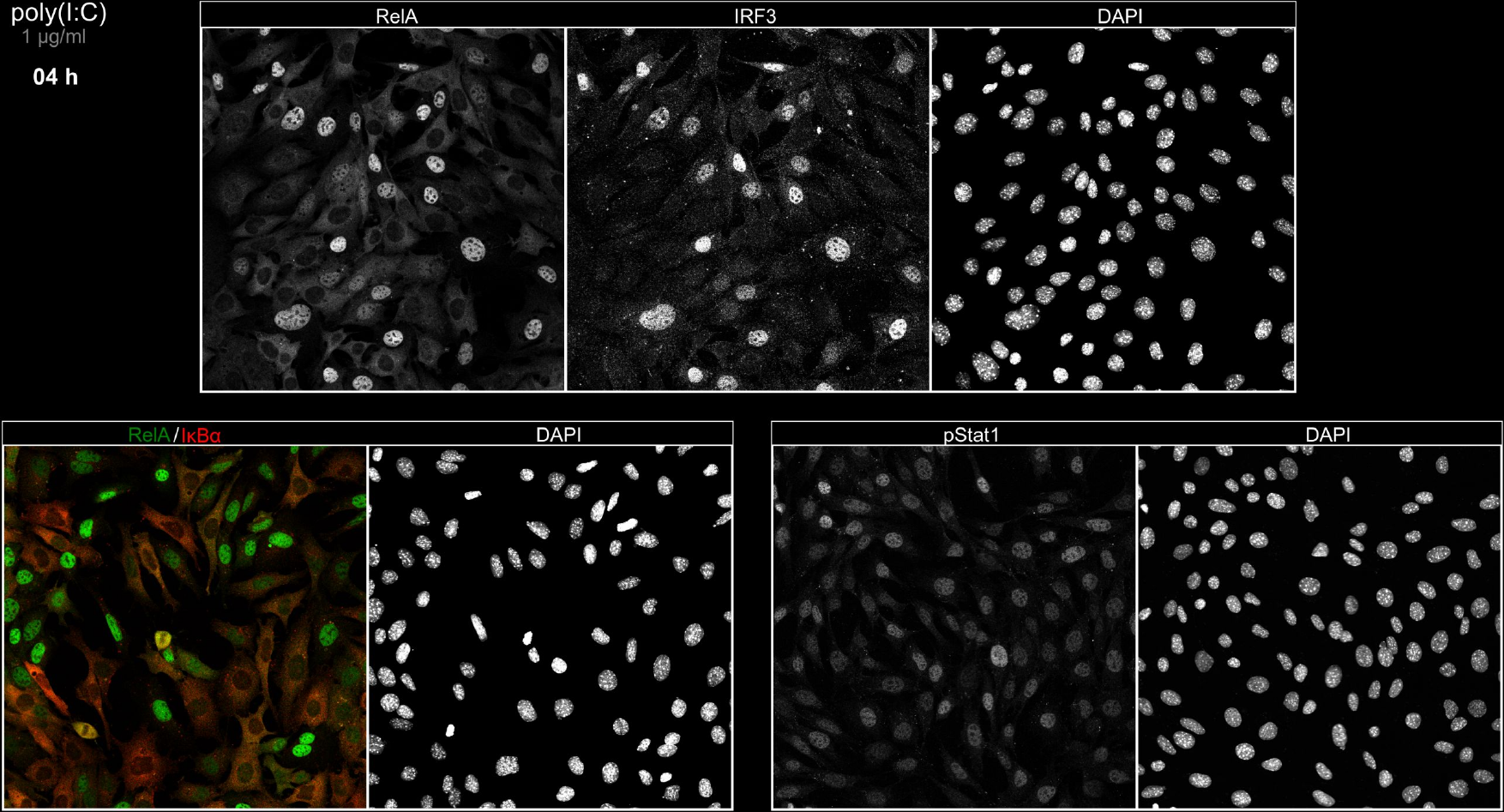

## Slide 4
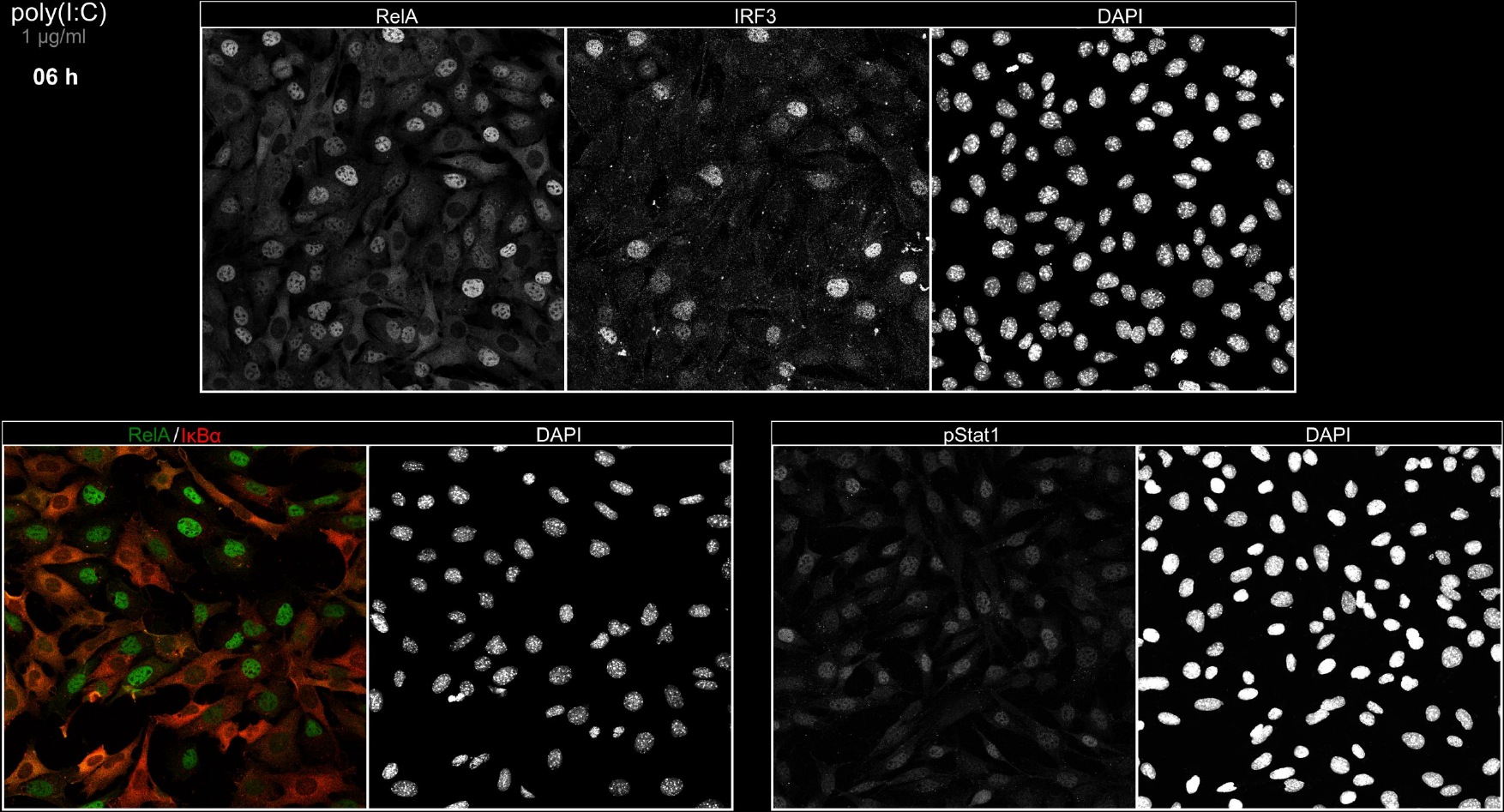

## Slide 5
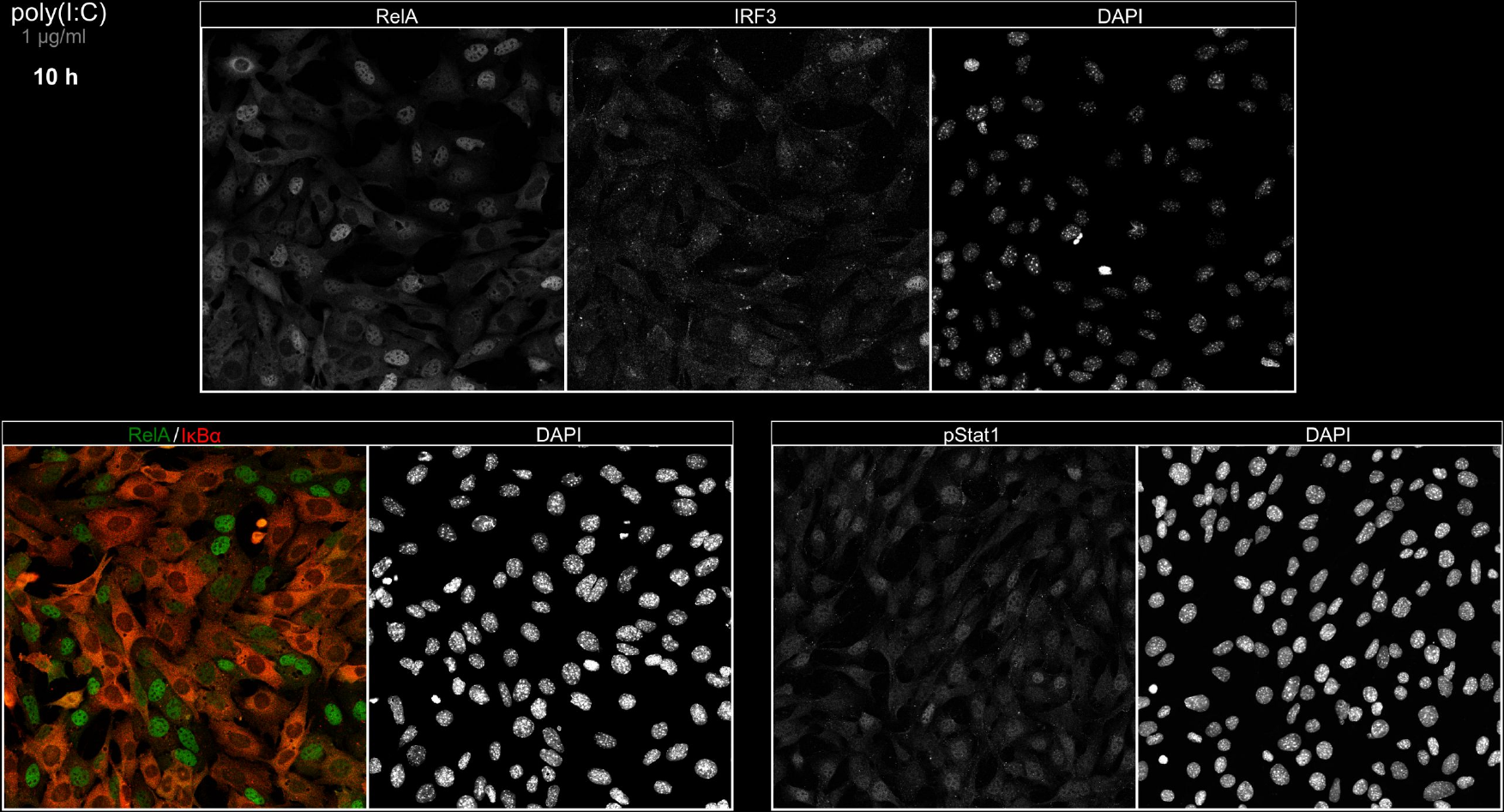

## Slide 6
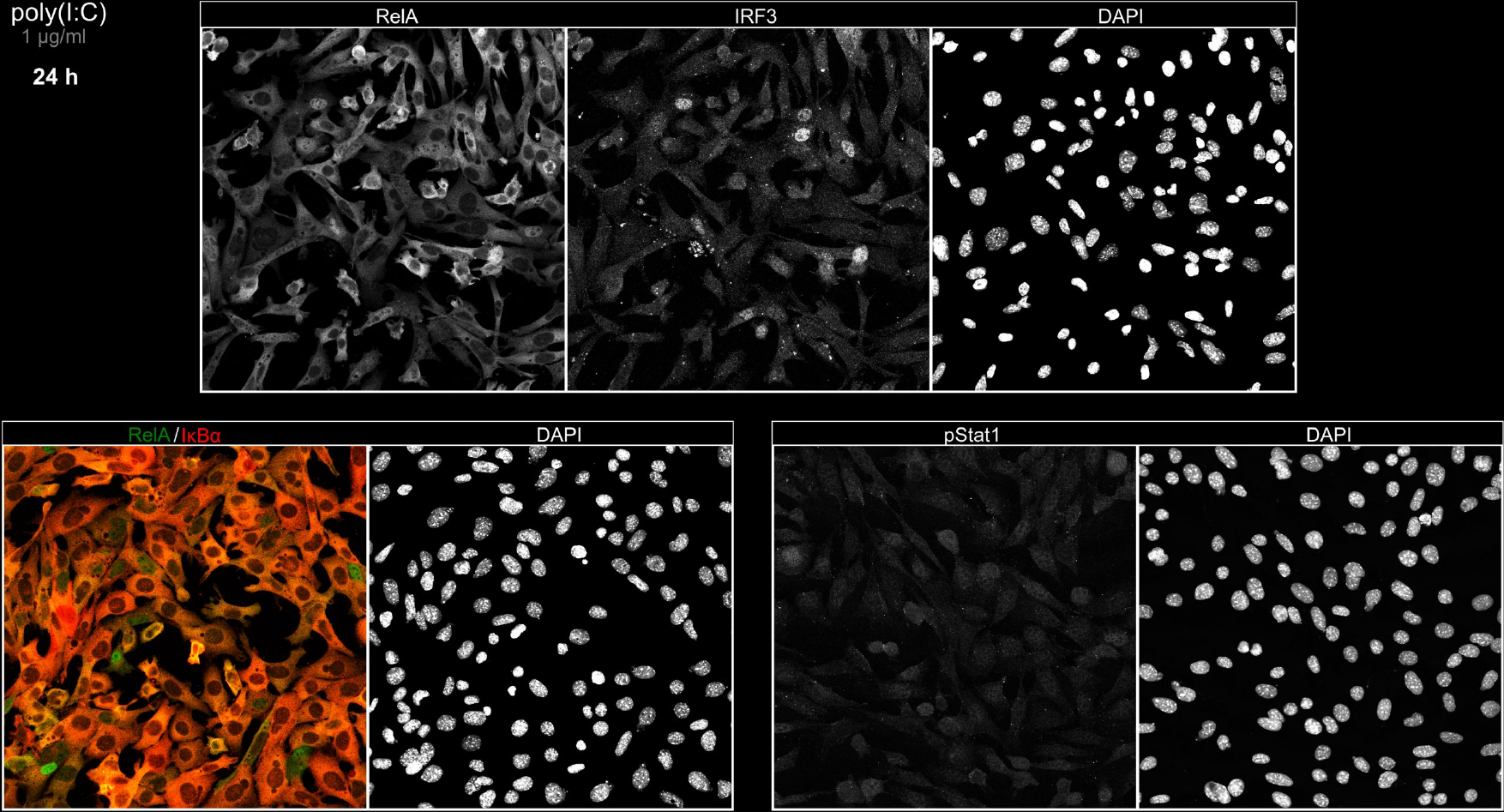

Supplement: Supplementary file 4 — Supplementary Data 1 [file 41467_2017_2640_MOESM4_ESM.ppt]

## Slide 1
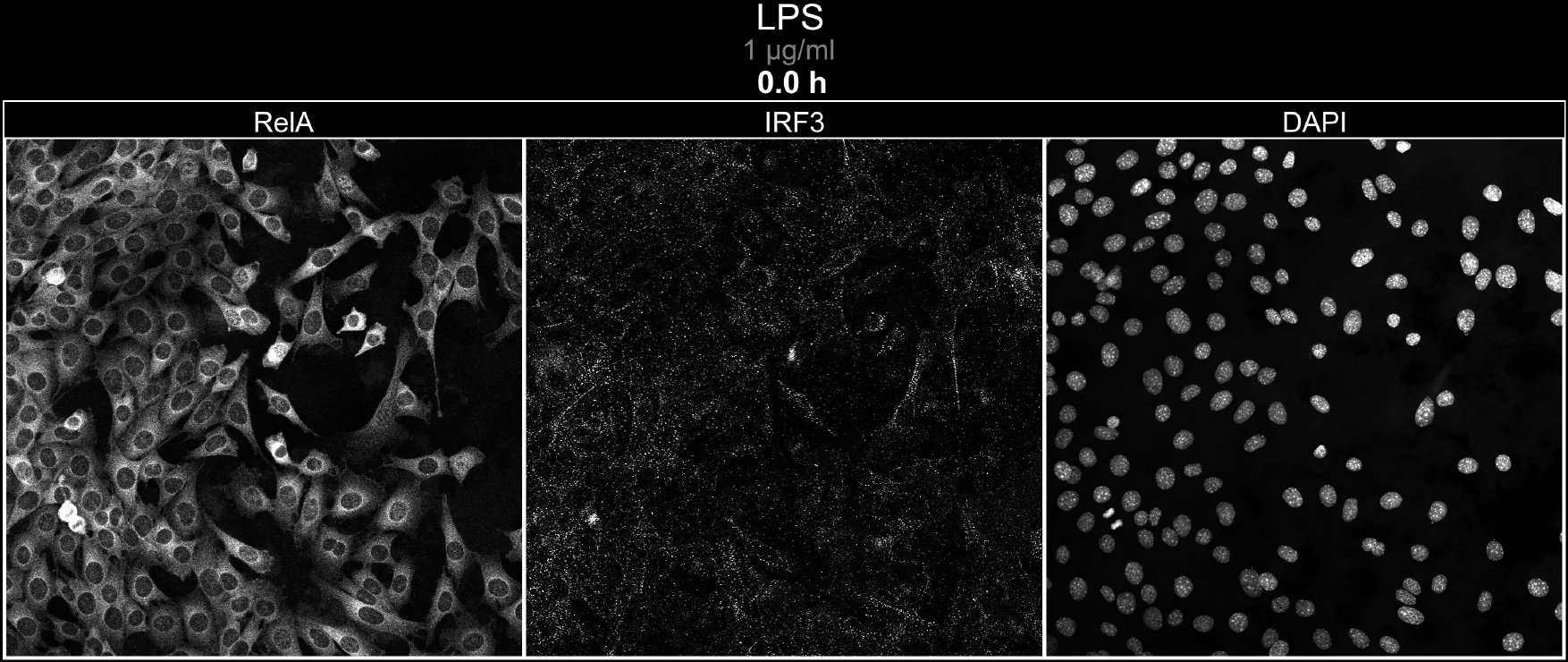

## Slide 2
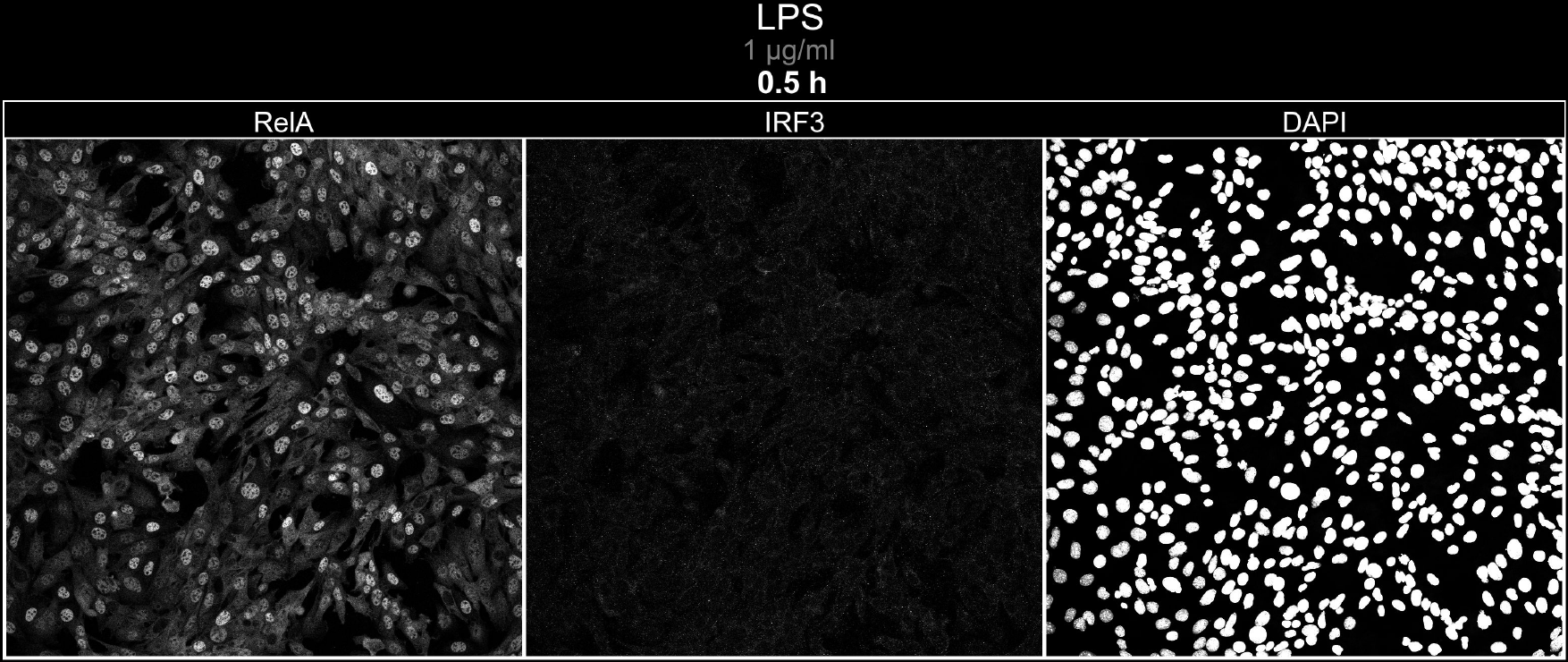

## Slide 3
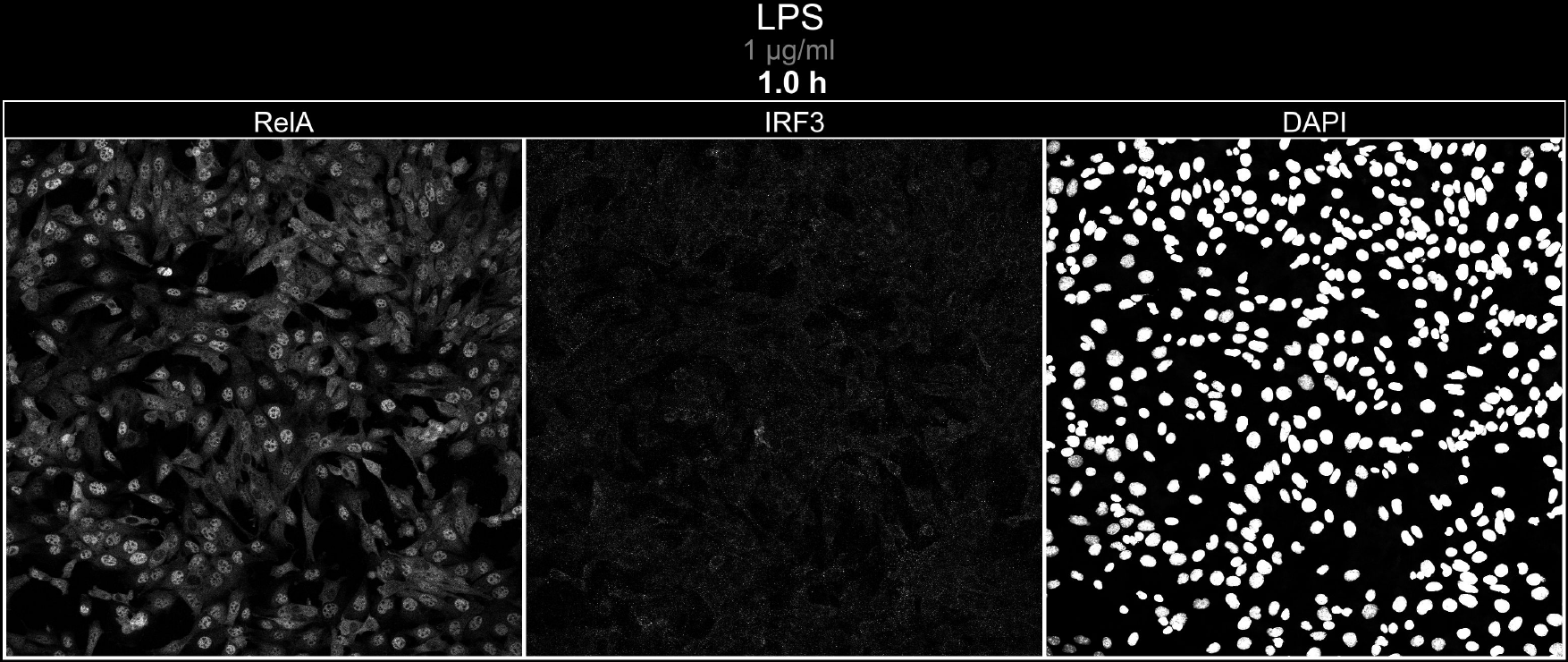

## Slide 4
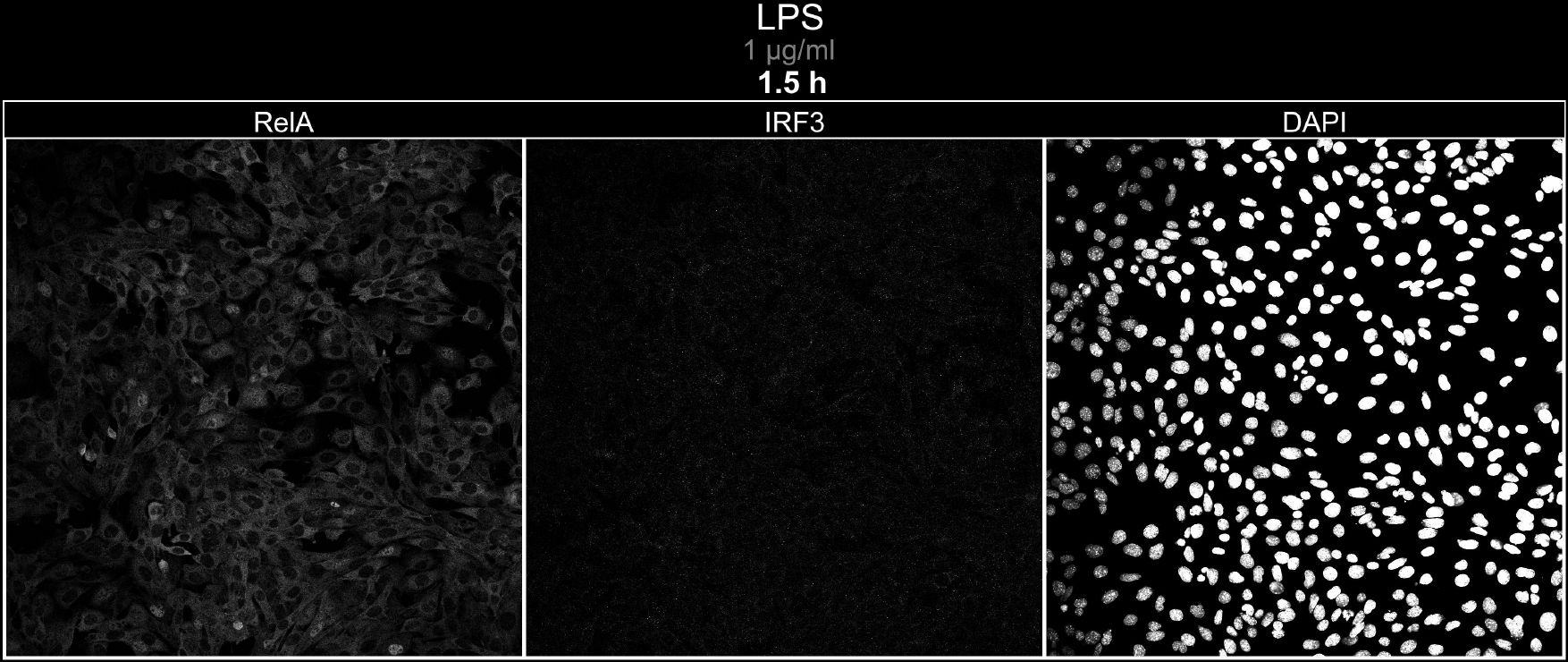

## Slide 5
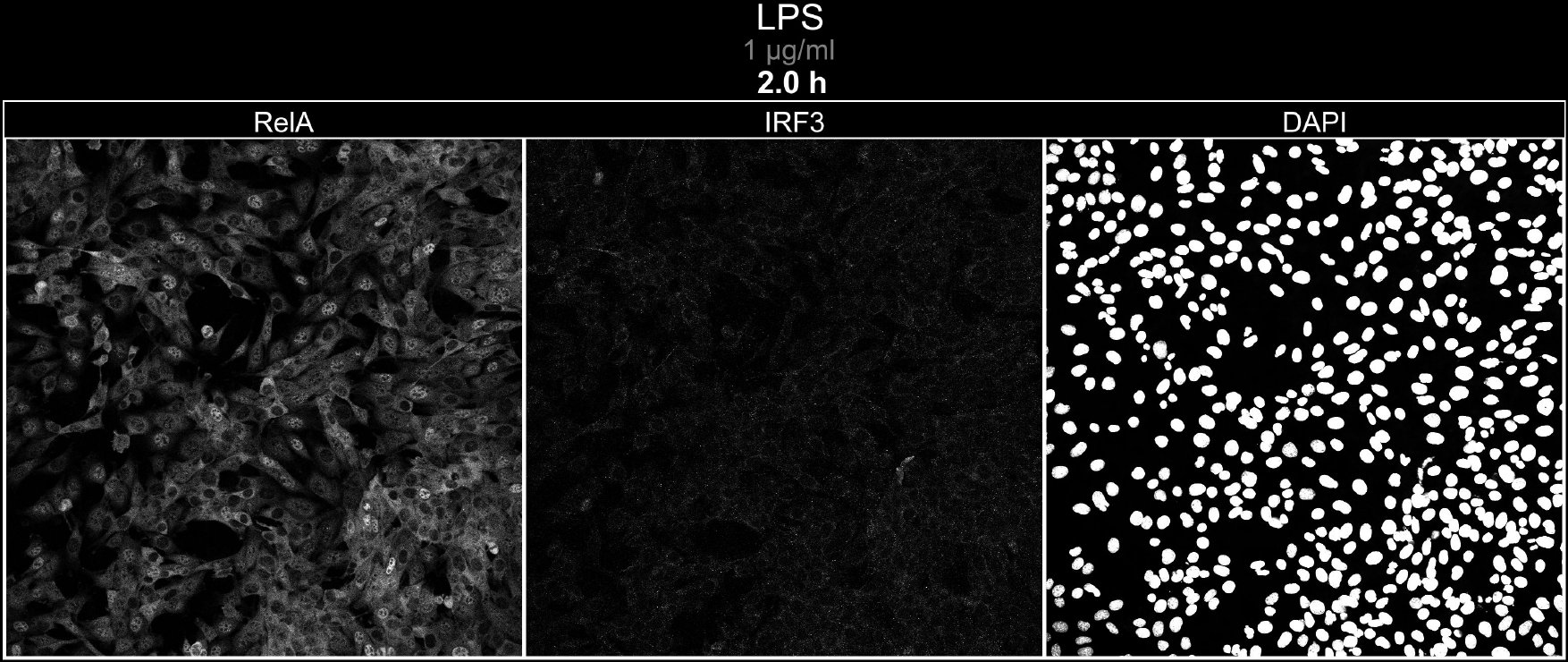

## Slide 6
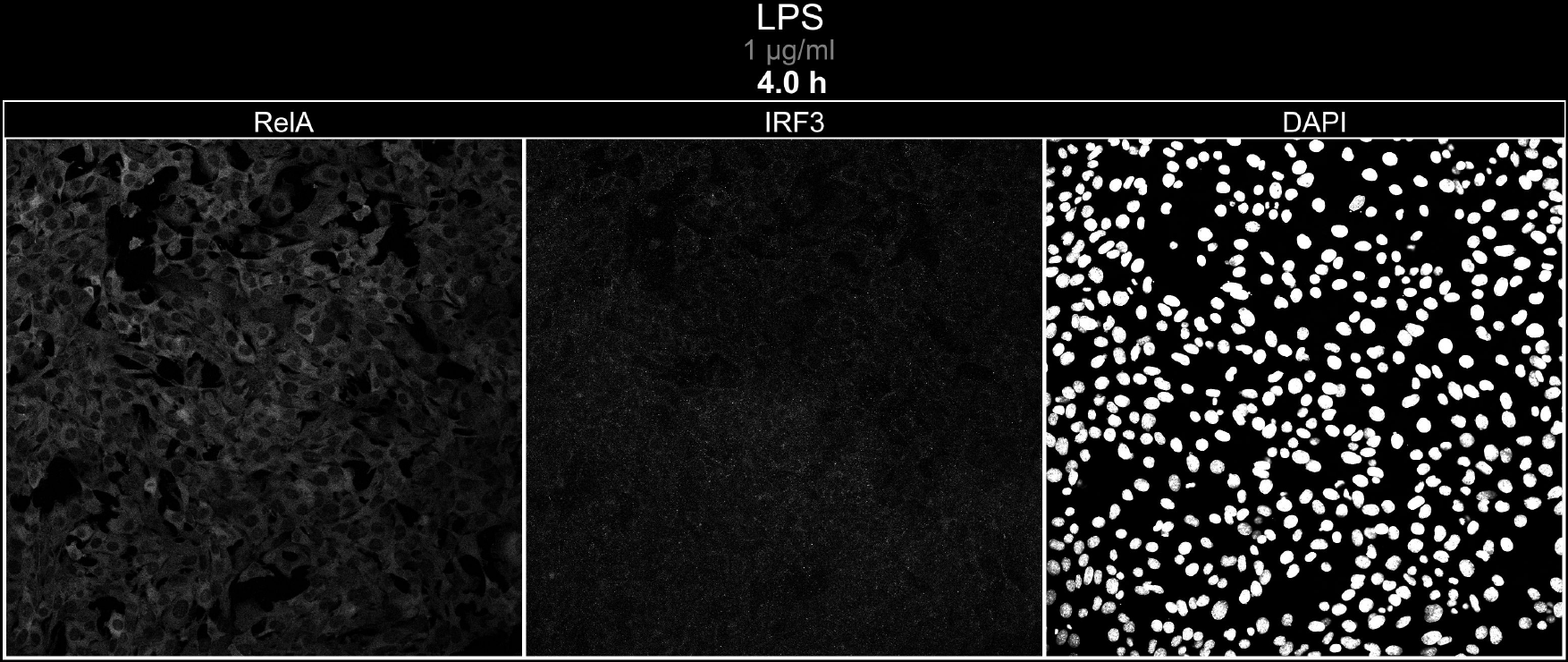

## Slide 7
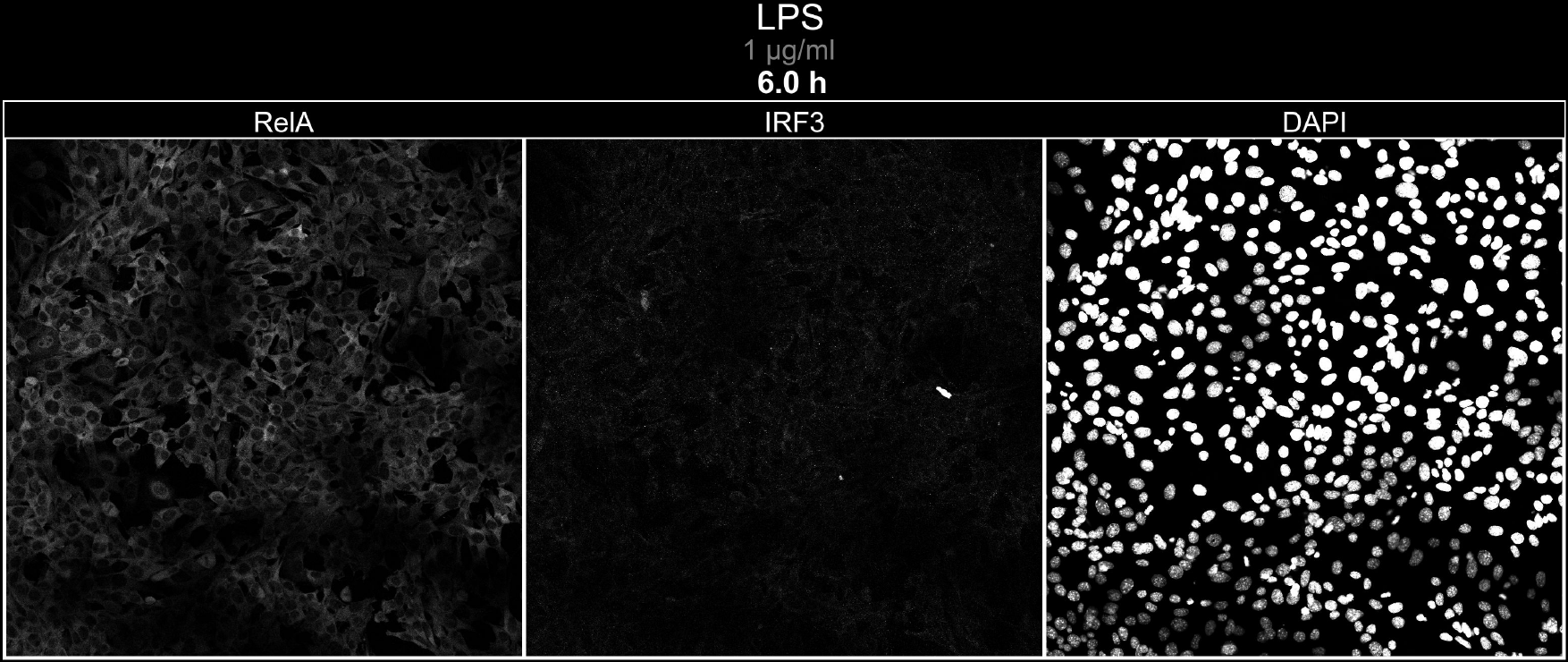

Supplement: Supplementary file 5 — Supplementary Data 2 [file 41467_2017_2640_MOESM5_ESM.ppt]

## Slide 1
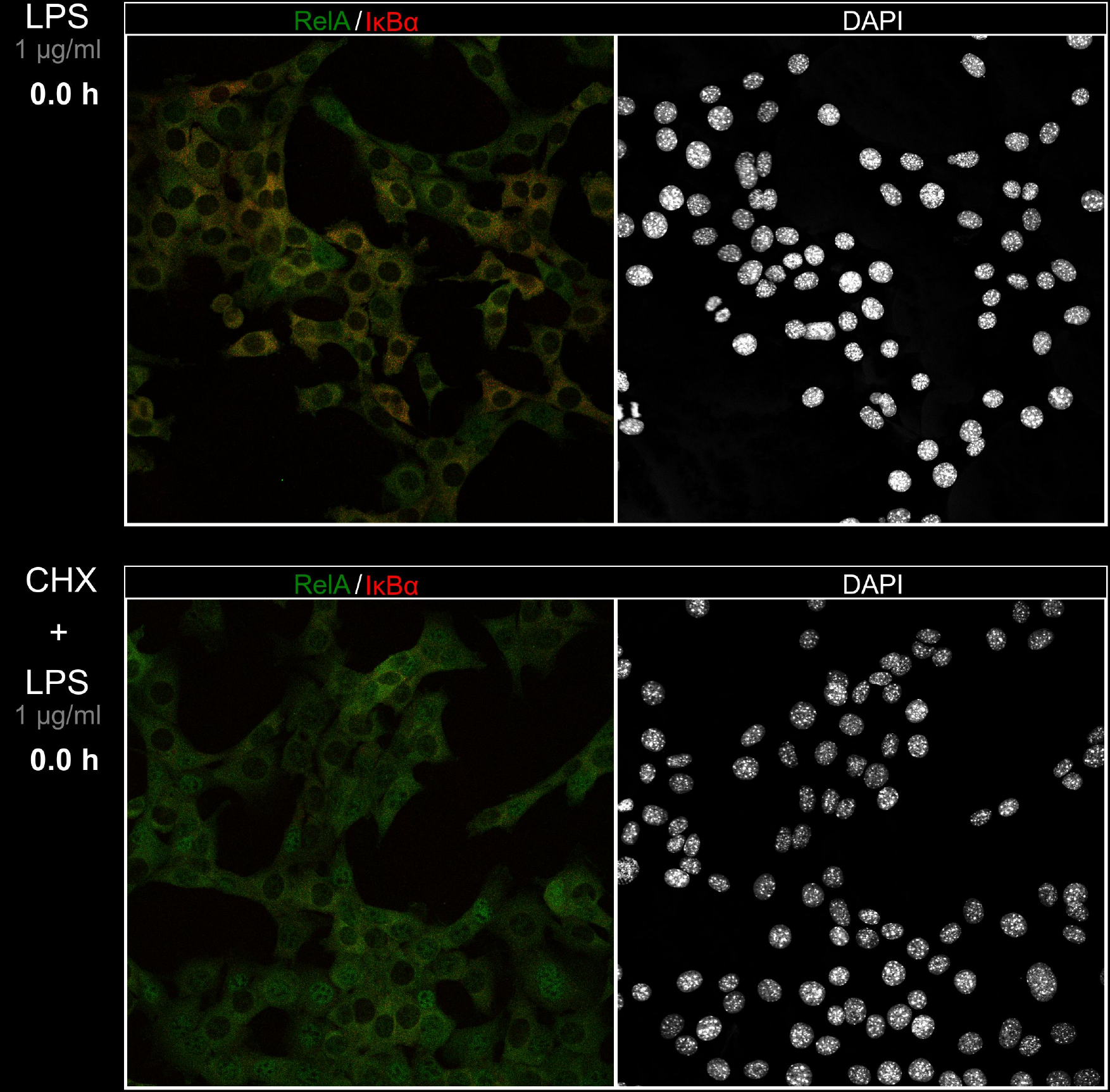

## Slide 2
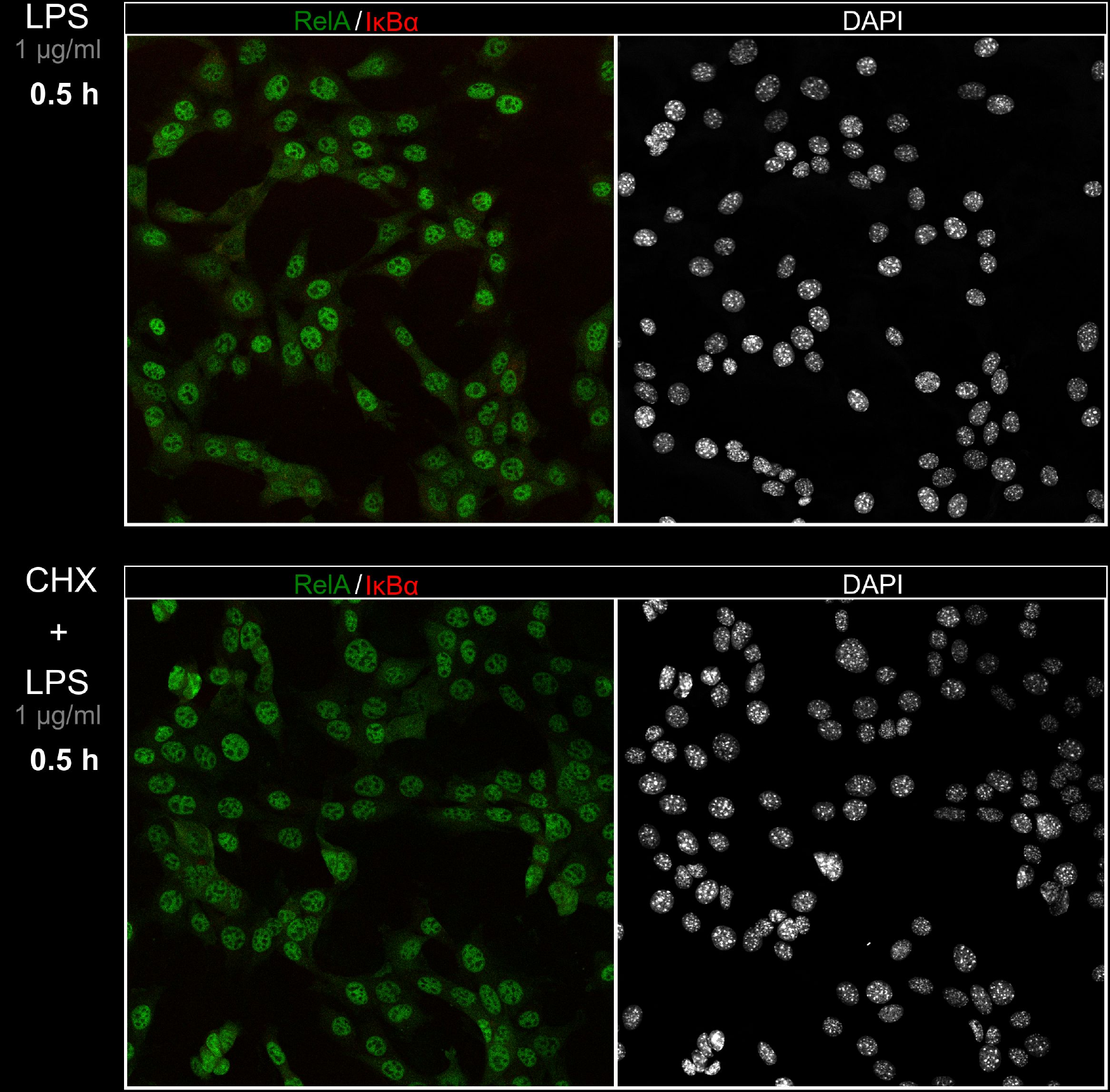

## Slide 3
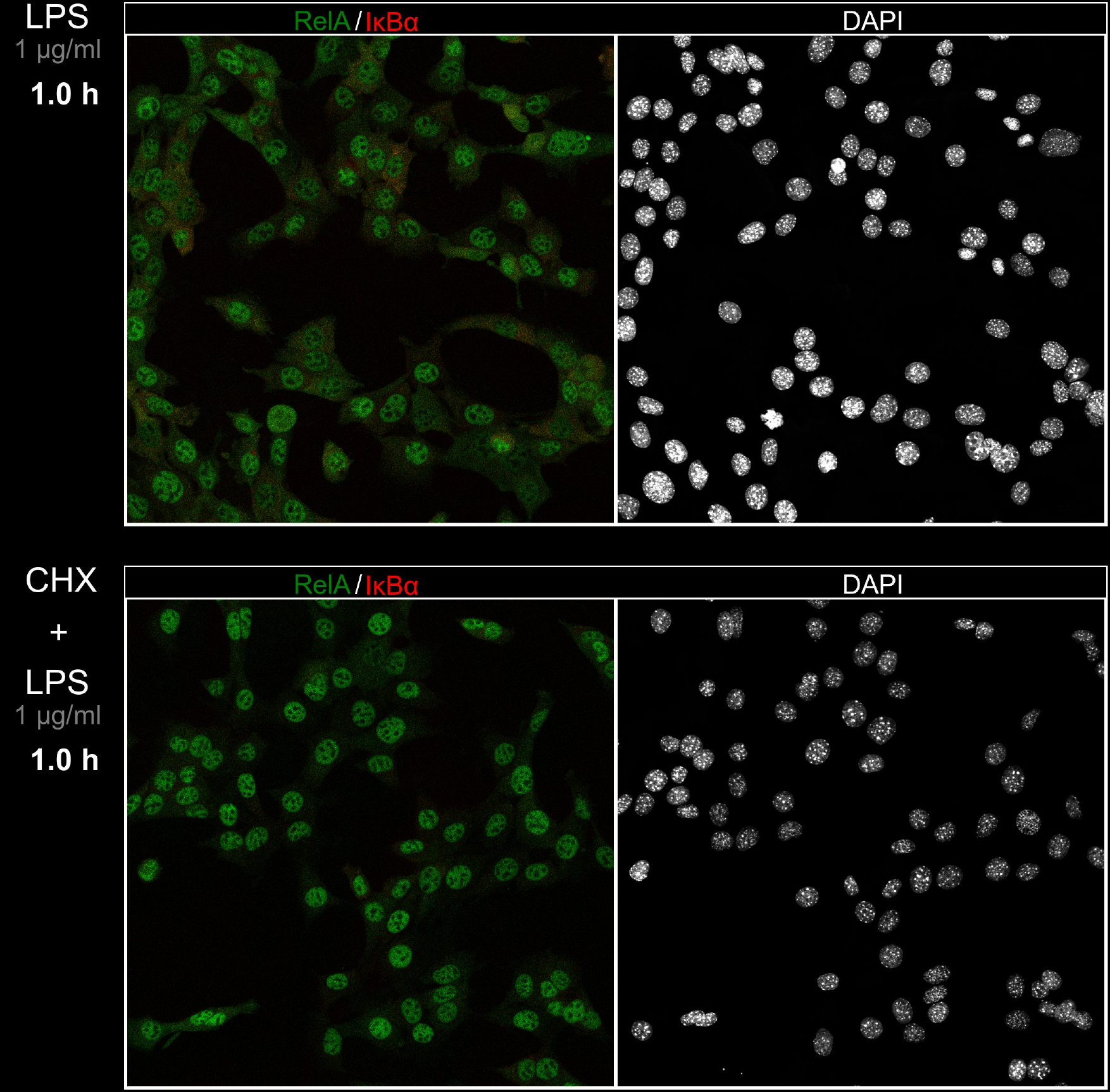

## Slide 4
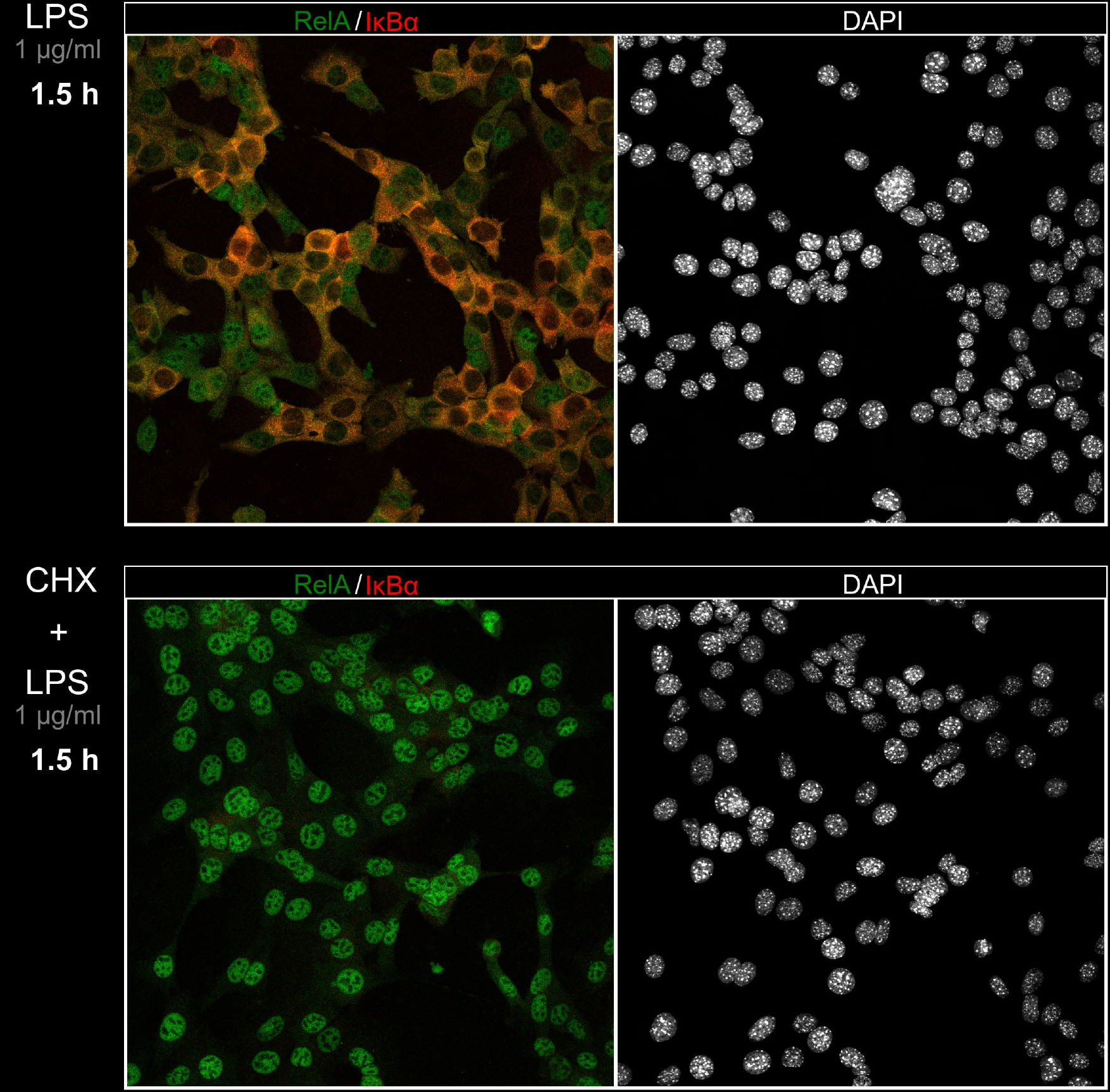

## Slide 5
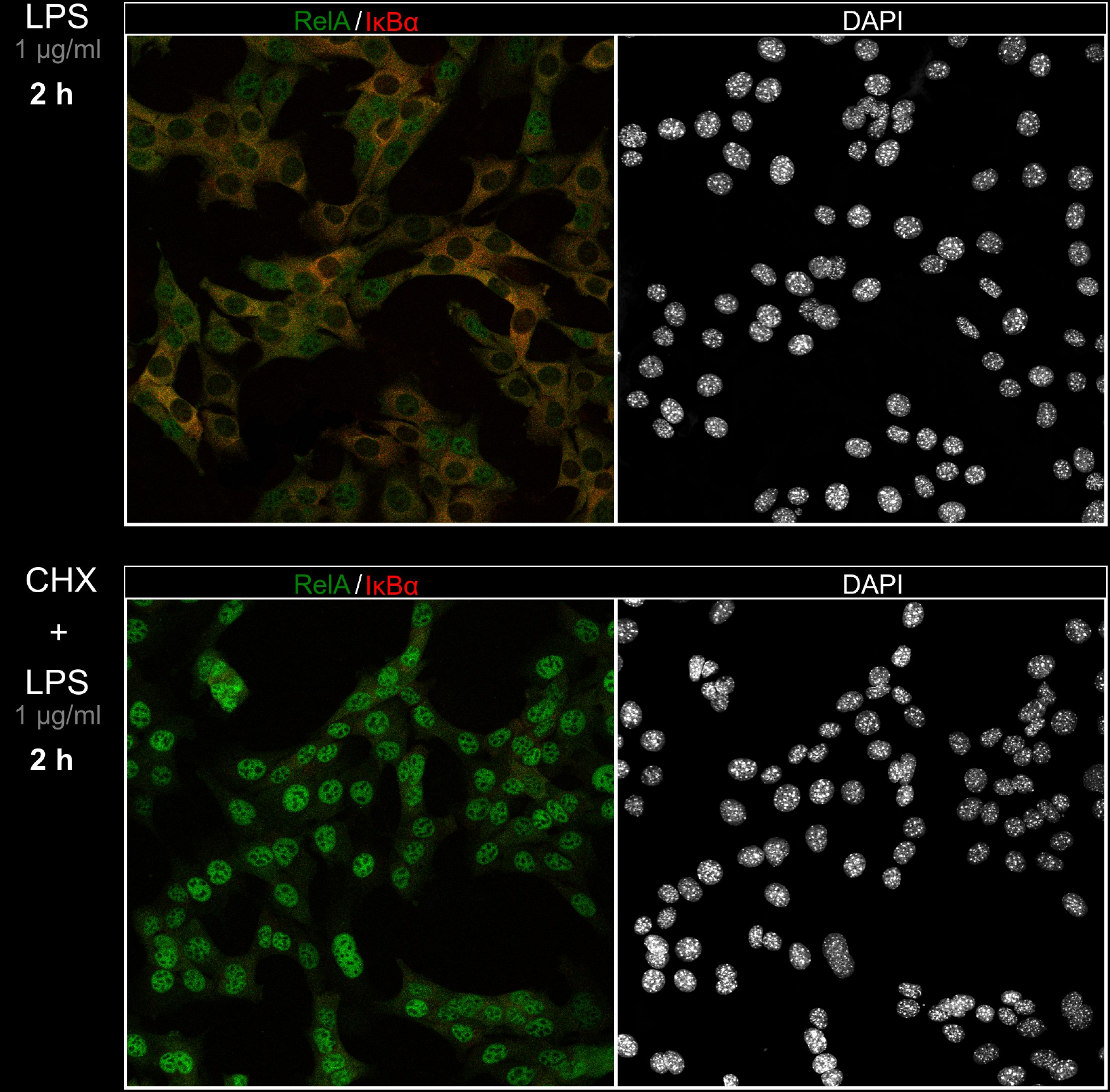

## Slide 6
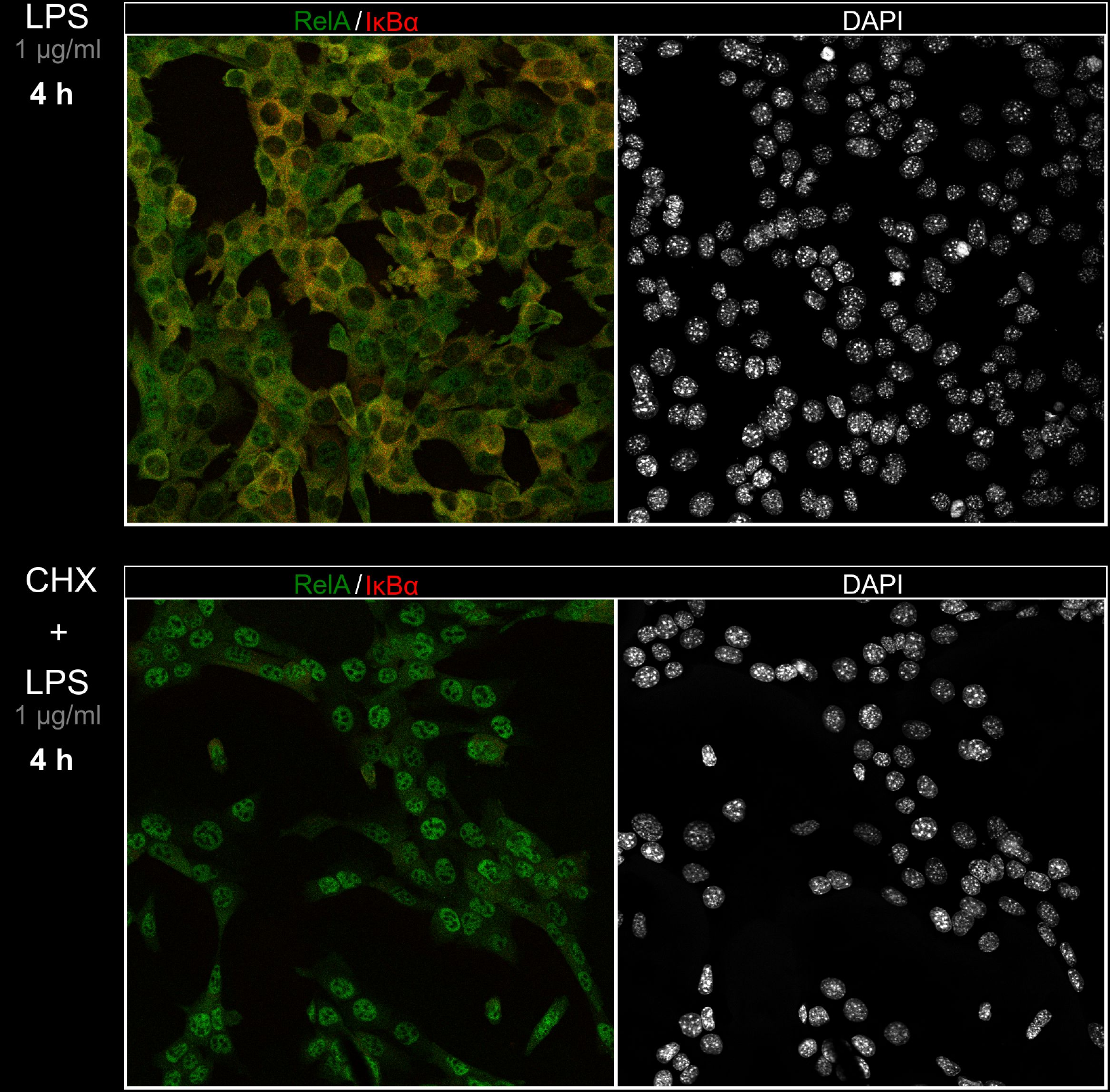

## Slide 7
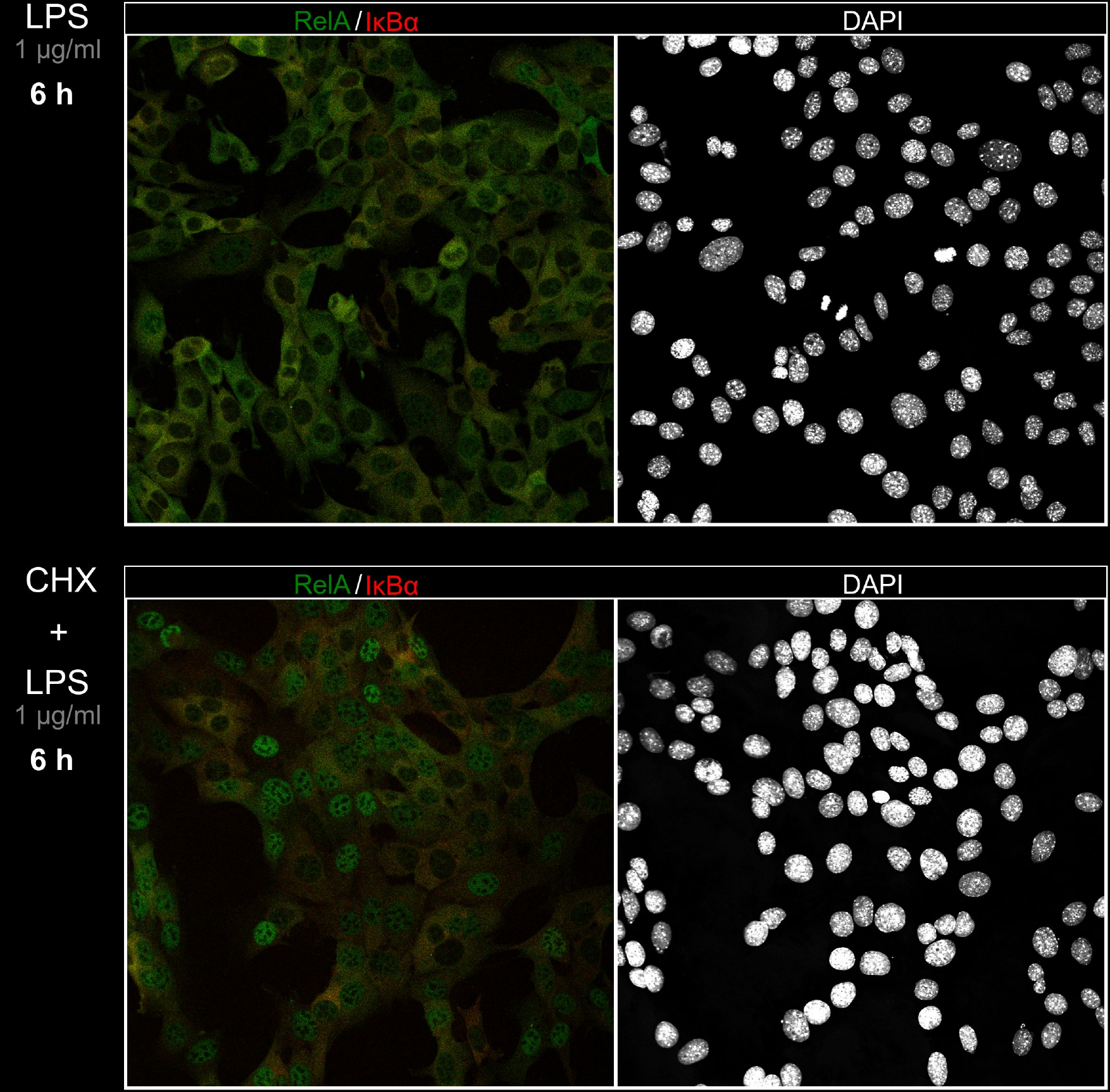

Supplement: Supplementary file 6 — Supplementary Data 3 [file 41467_2017_2640_MOESM6_ESM.ppt]

## Slide 1
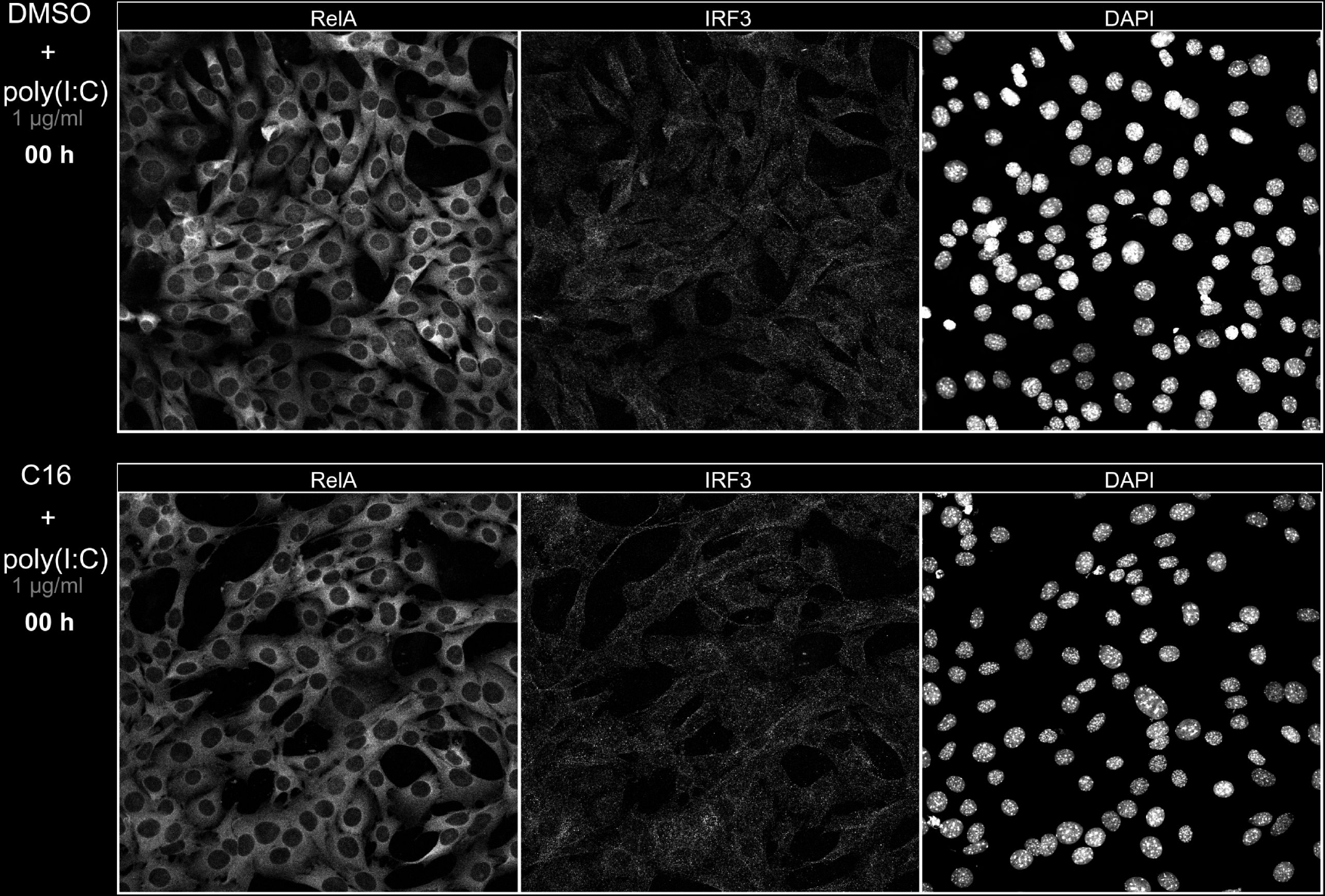

## Slide 2
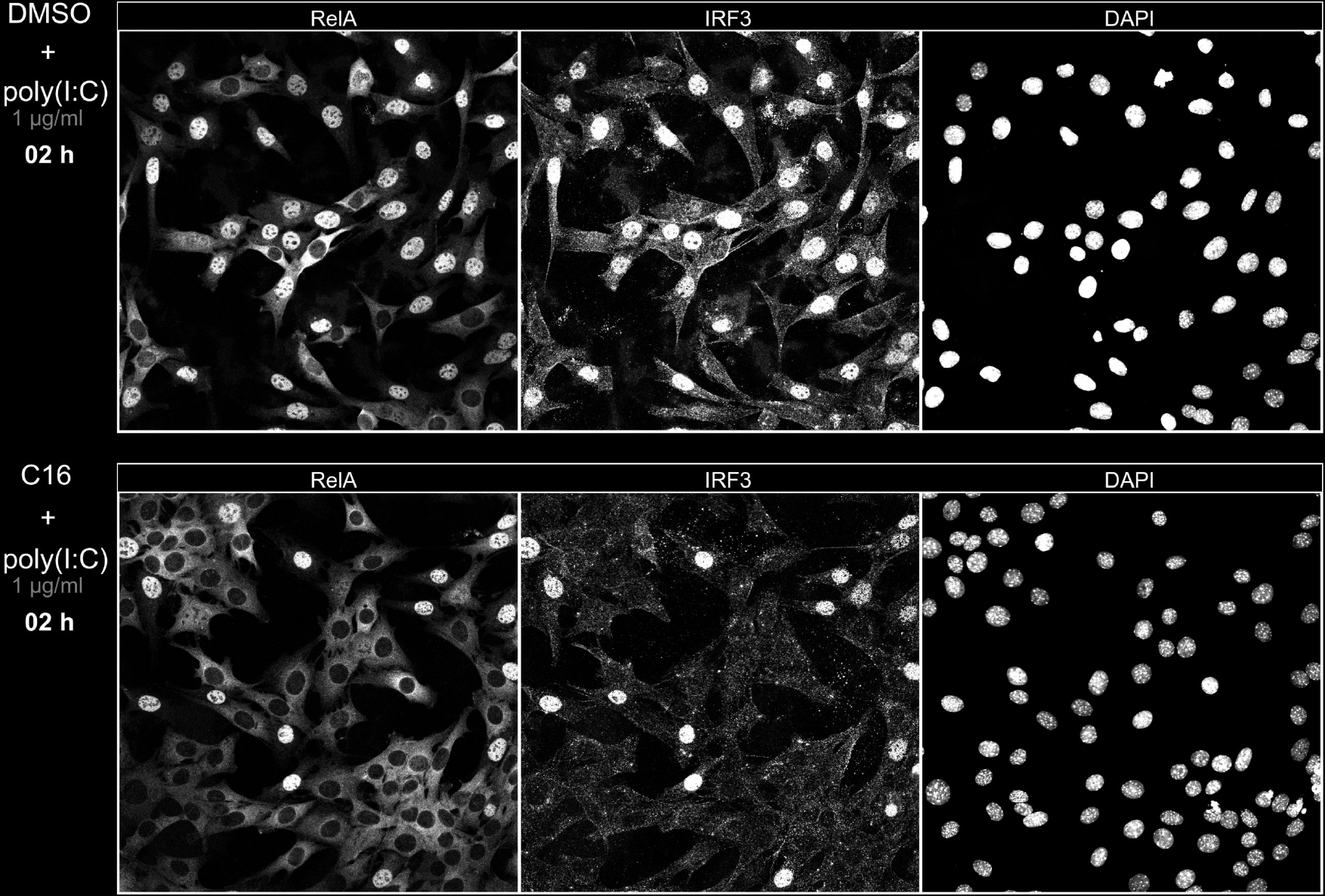

## Slide 3
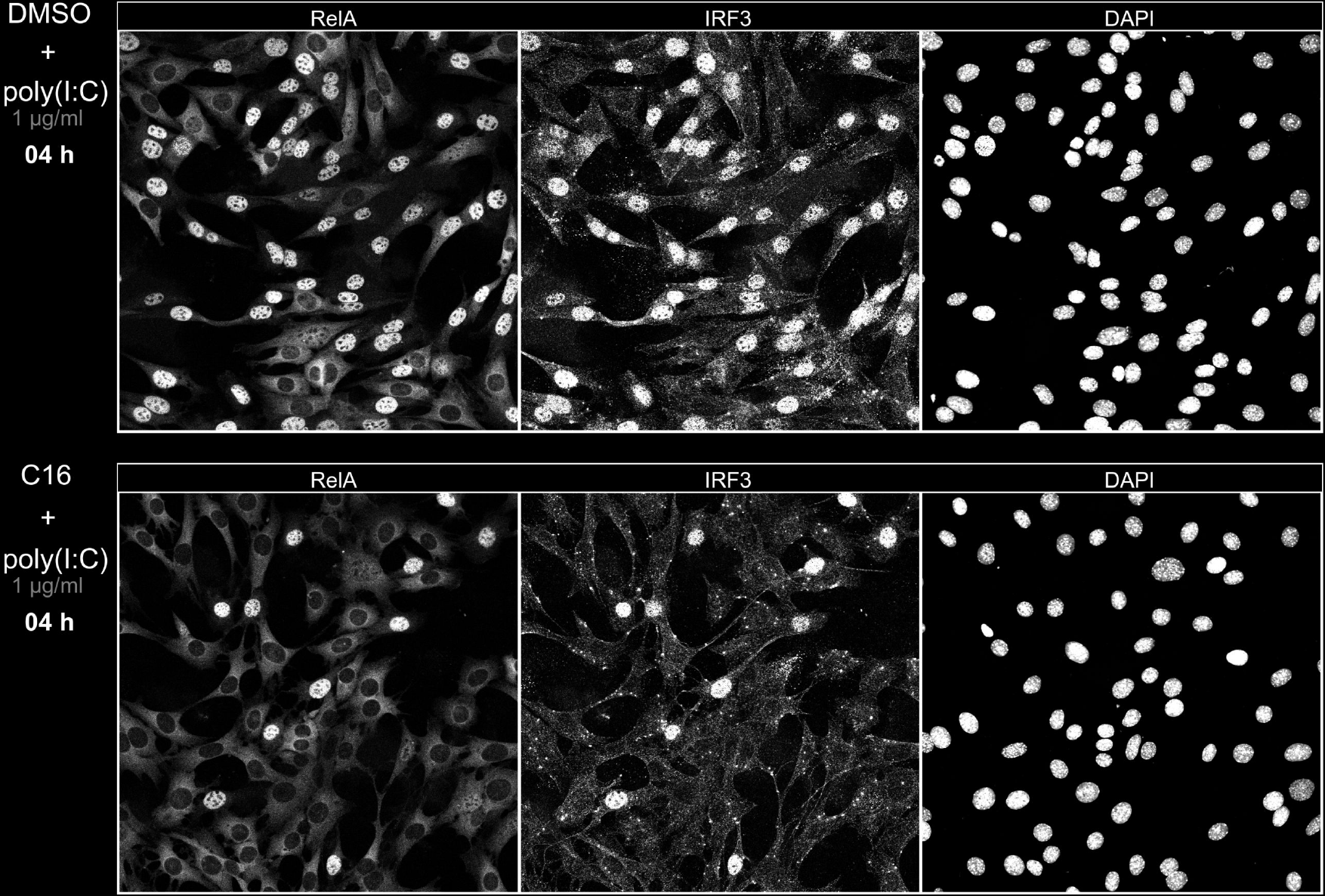

Supplement: Supplementary file 7 — Supplementary Data 4 [file 41467_2017_2640_MOESM7_ESM.ppt]

## Slide 1
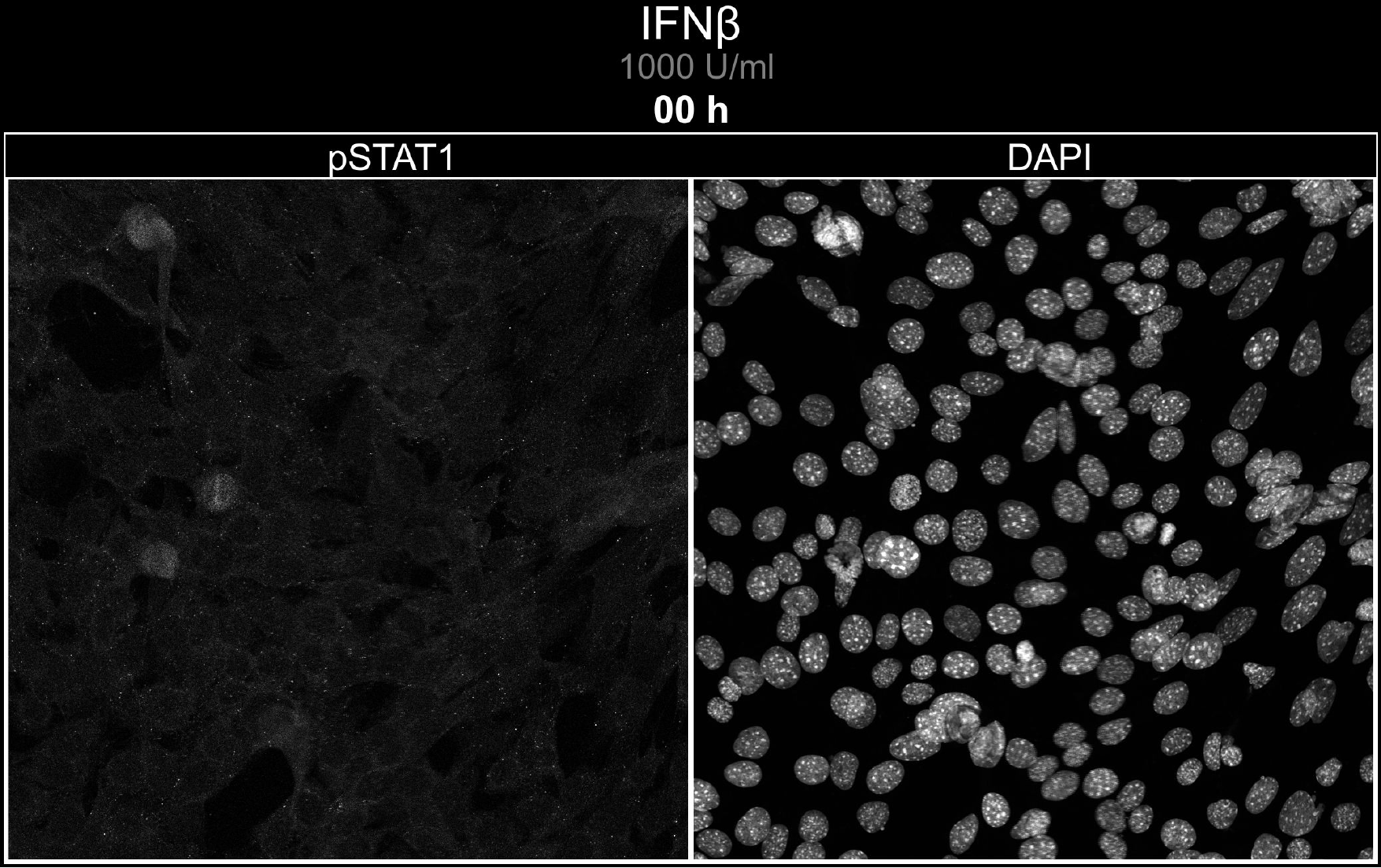

## Slide 2
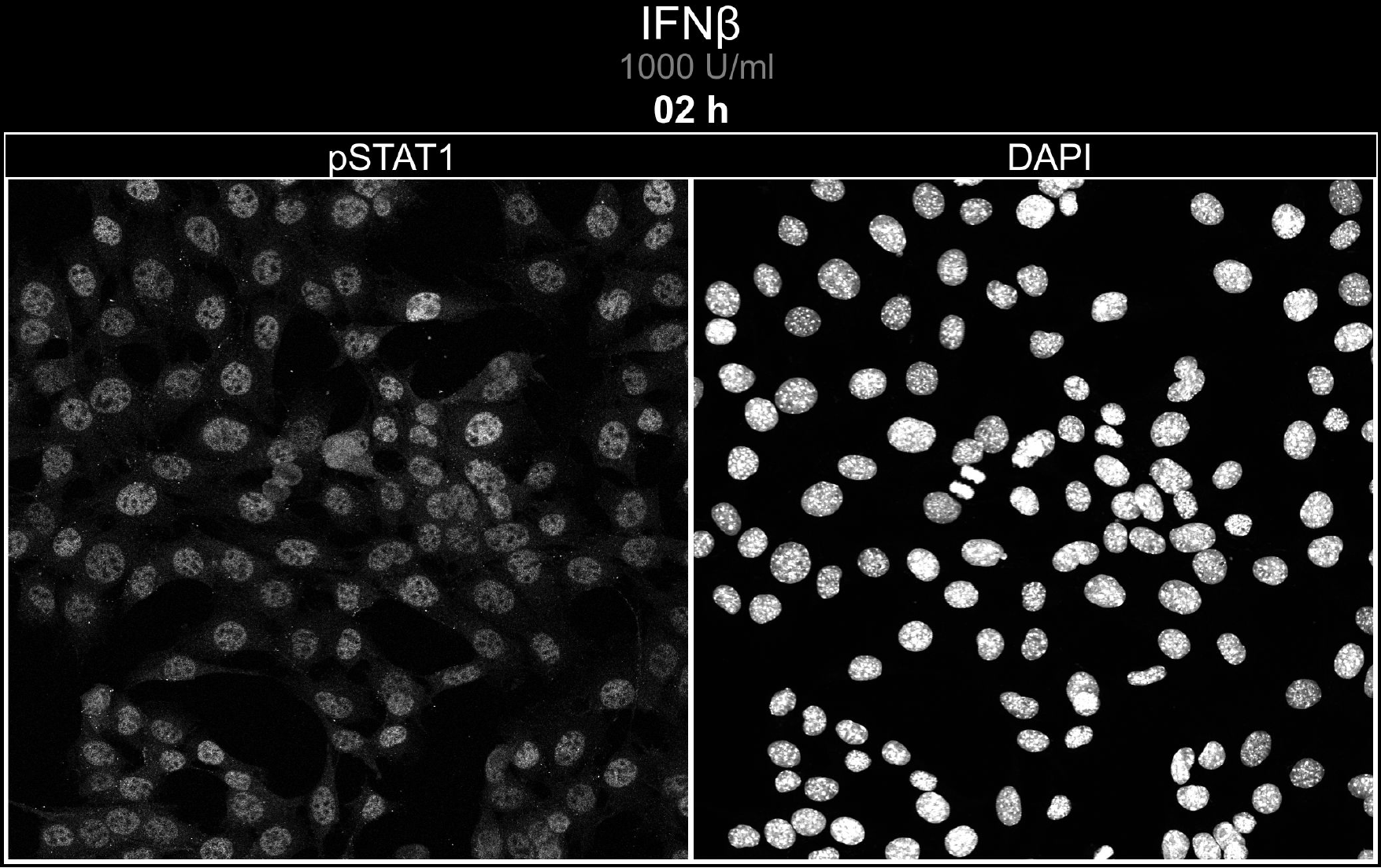

## Slide 3
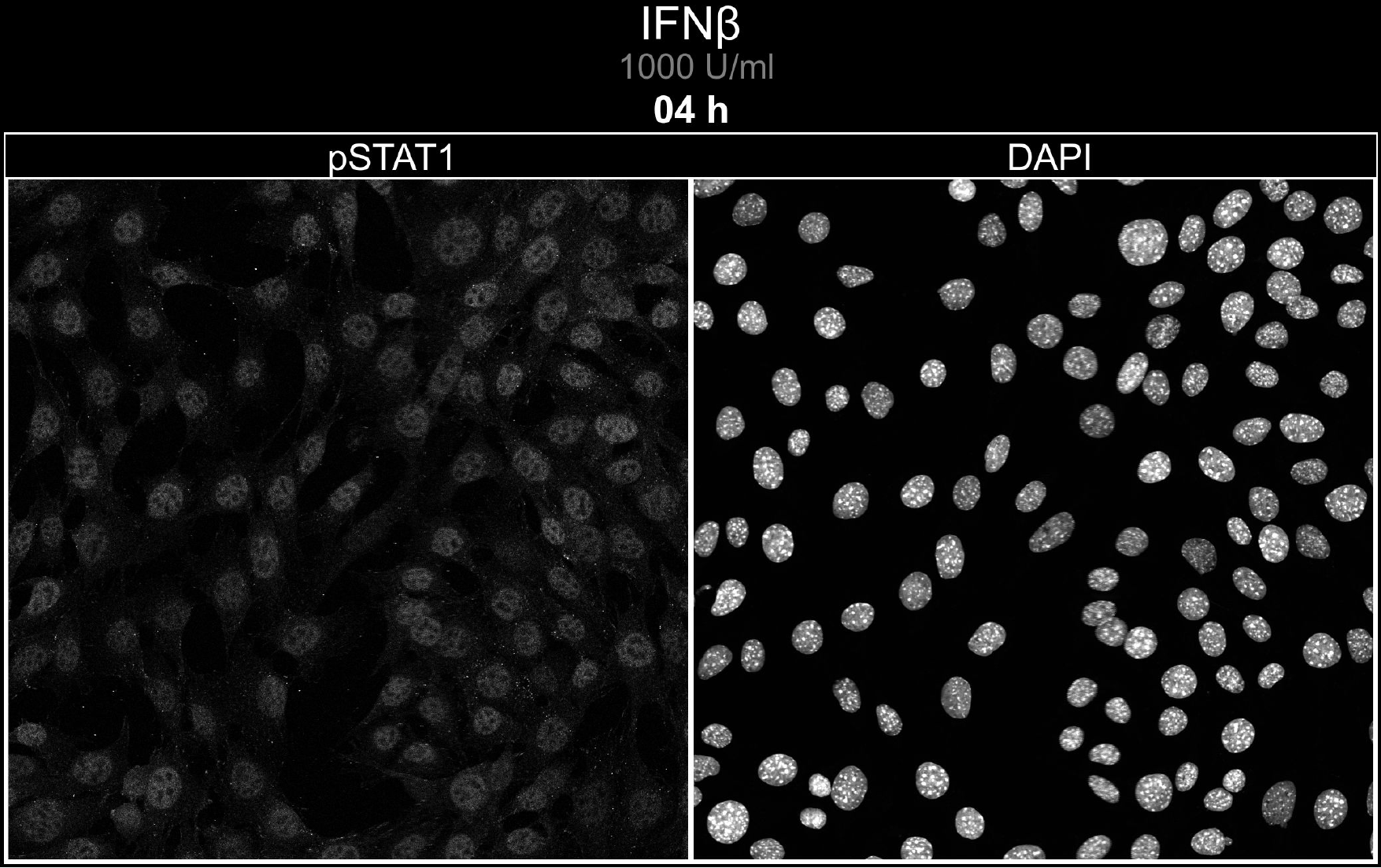

## Slide 4
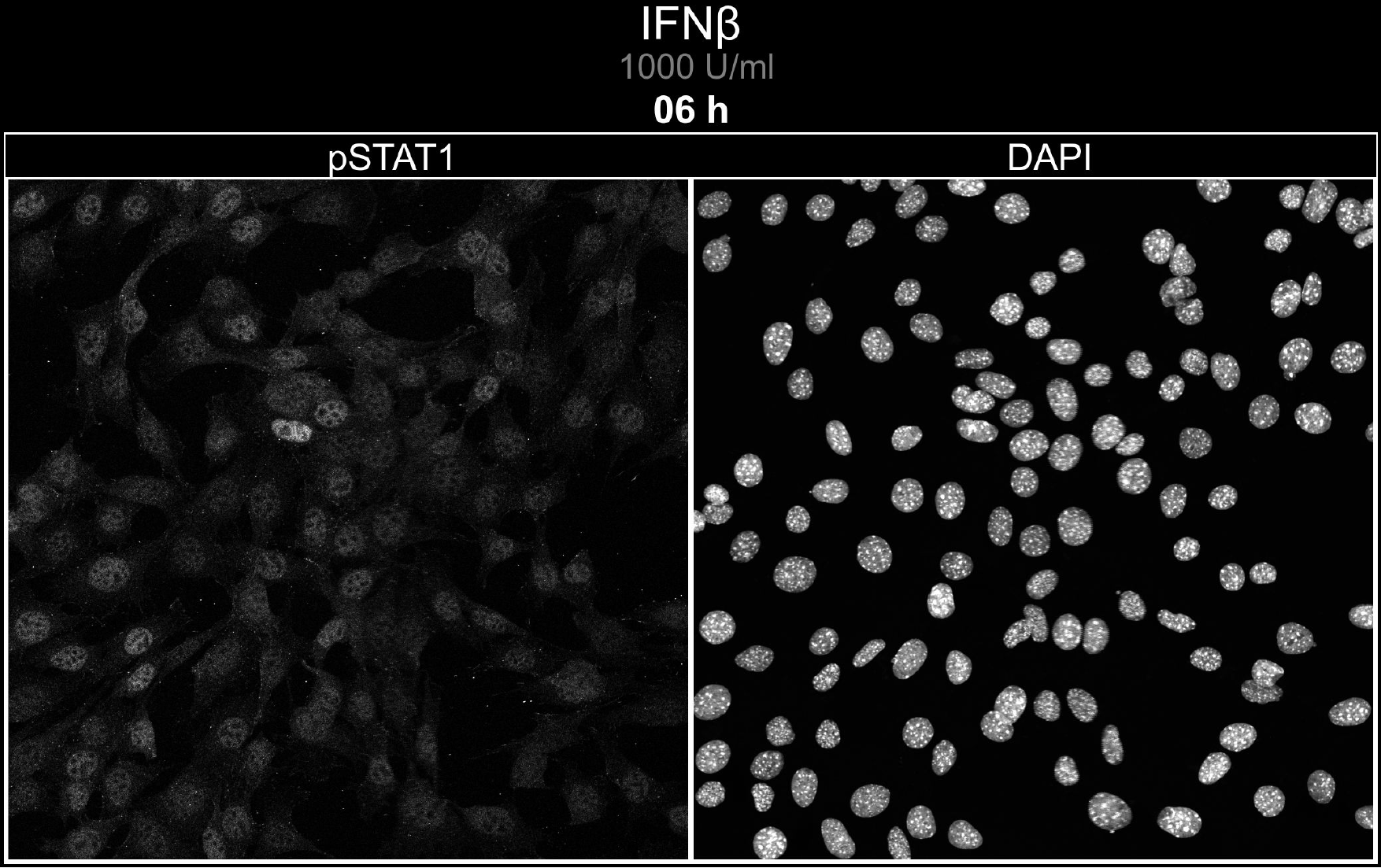

## Slide 5
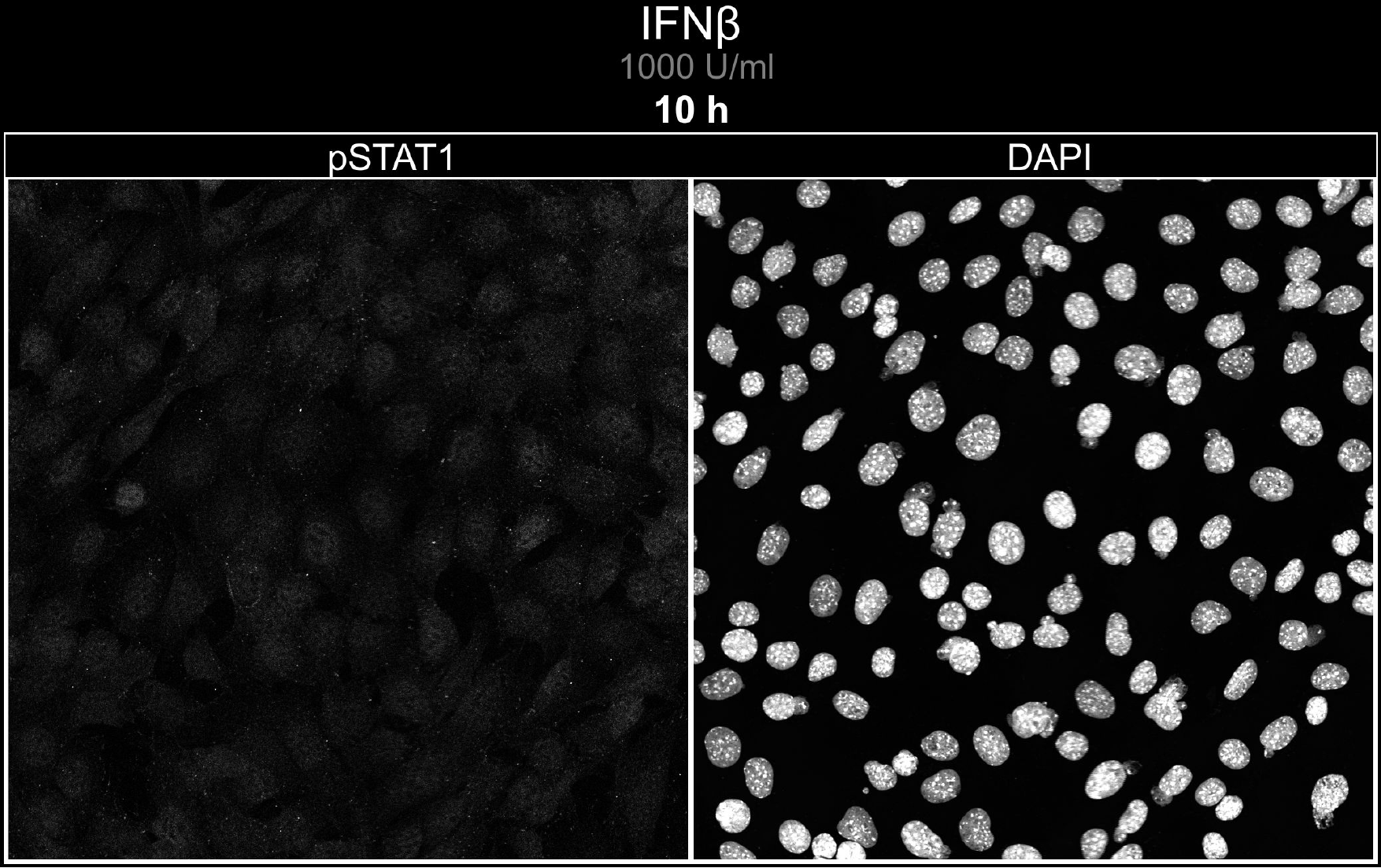

## Slide 6
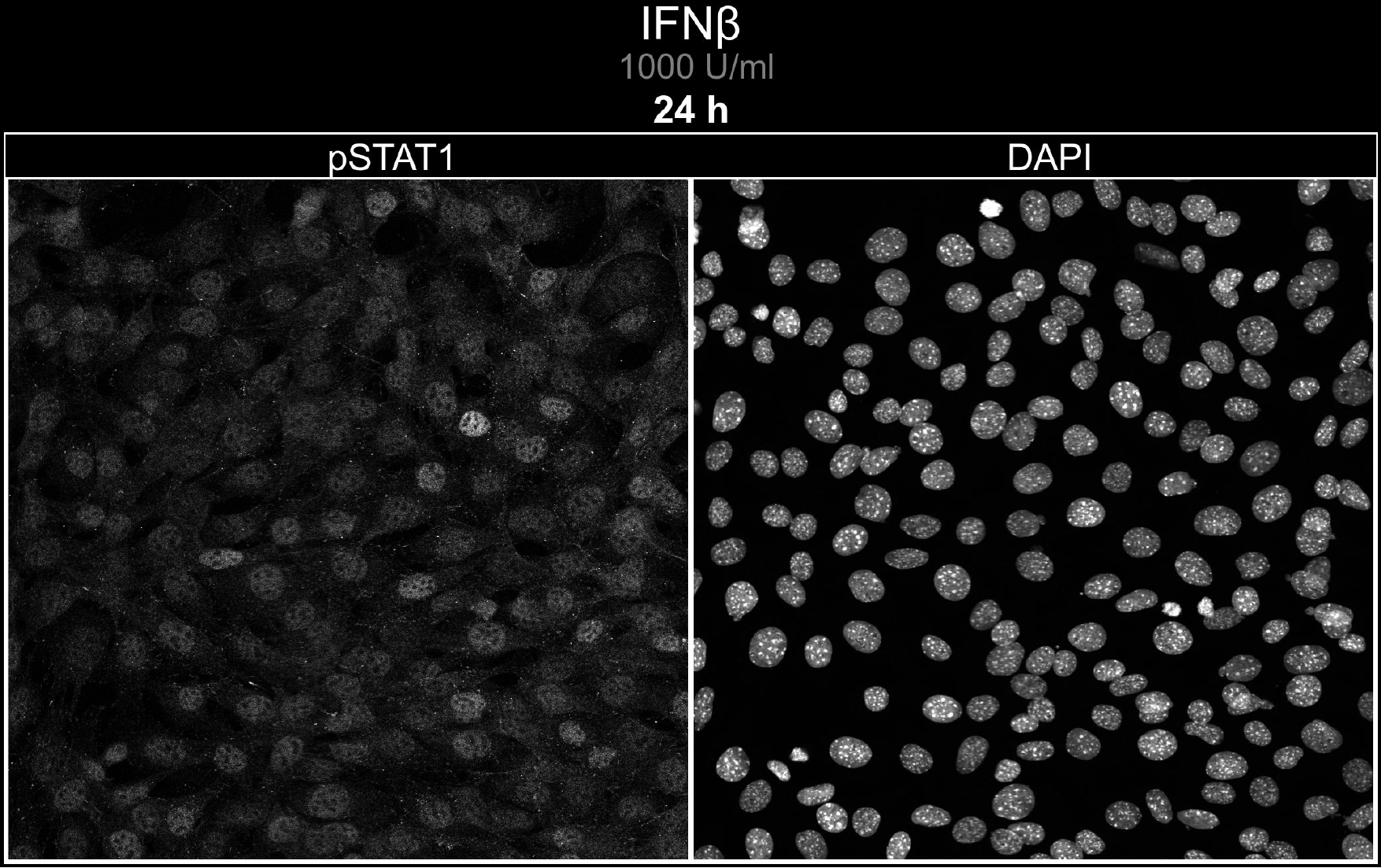

Supplement: Supplementary file 8 — Supplementary Data 5 [file 41467_2017_2640_MOESM8_ESM.ppt]

## Slide 1
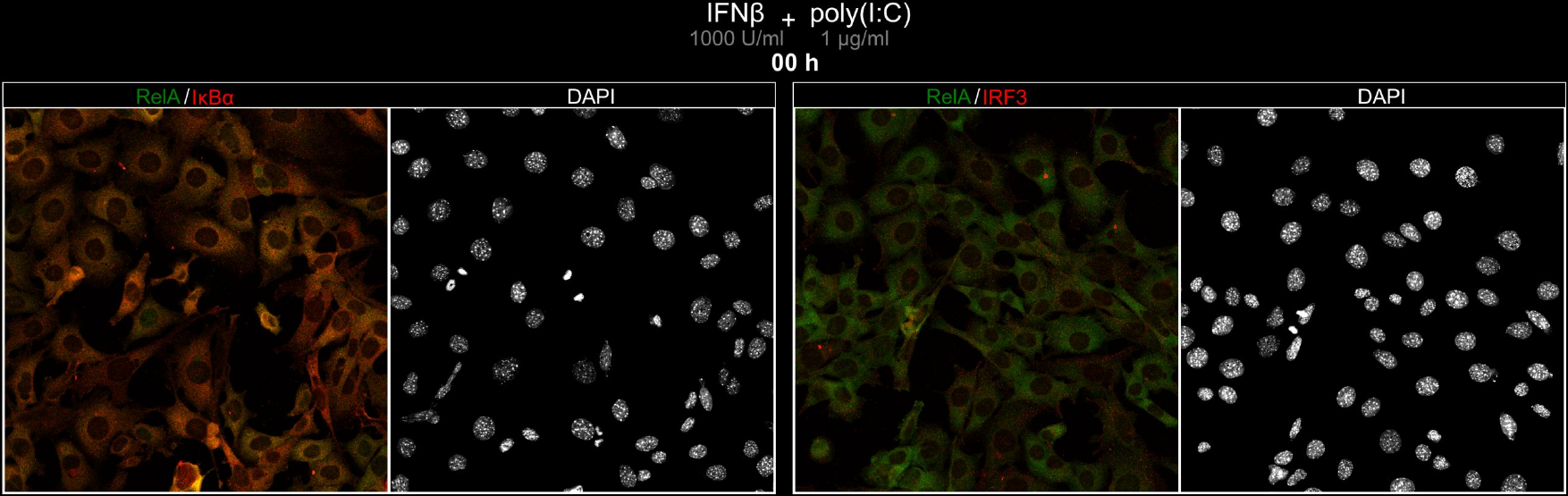

## Slide 2
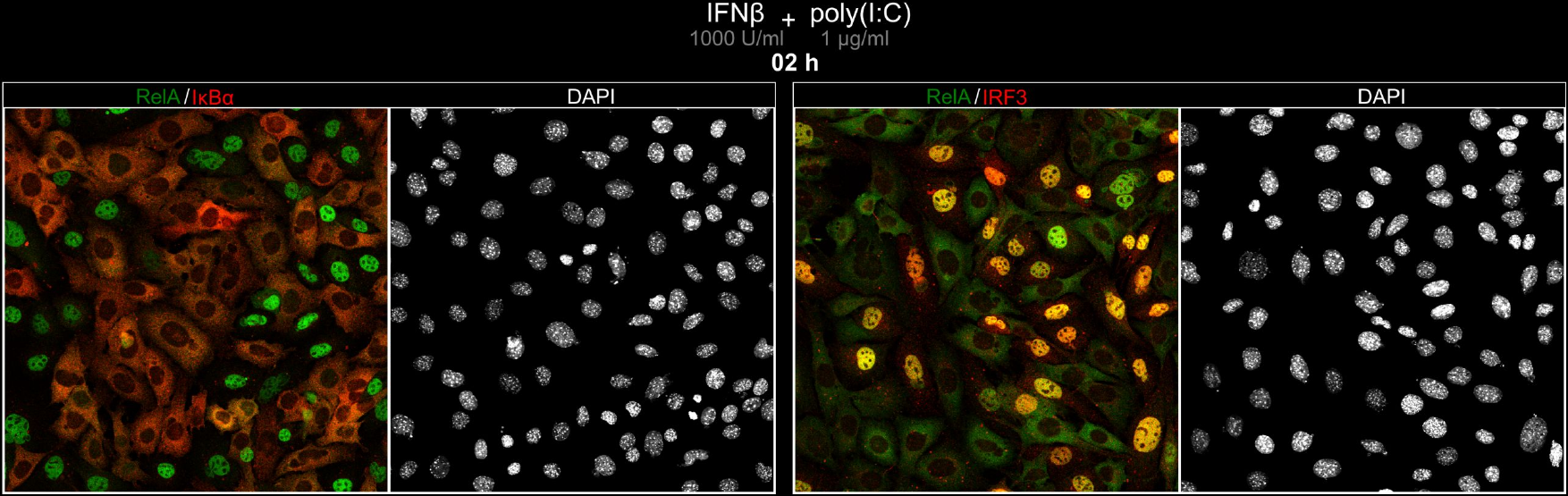

## Slide 3
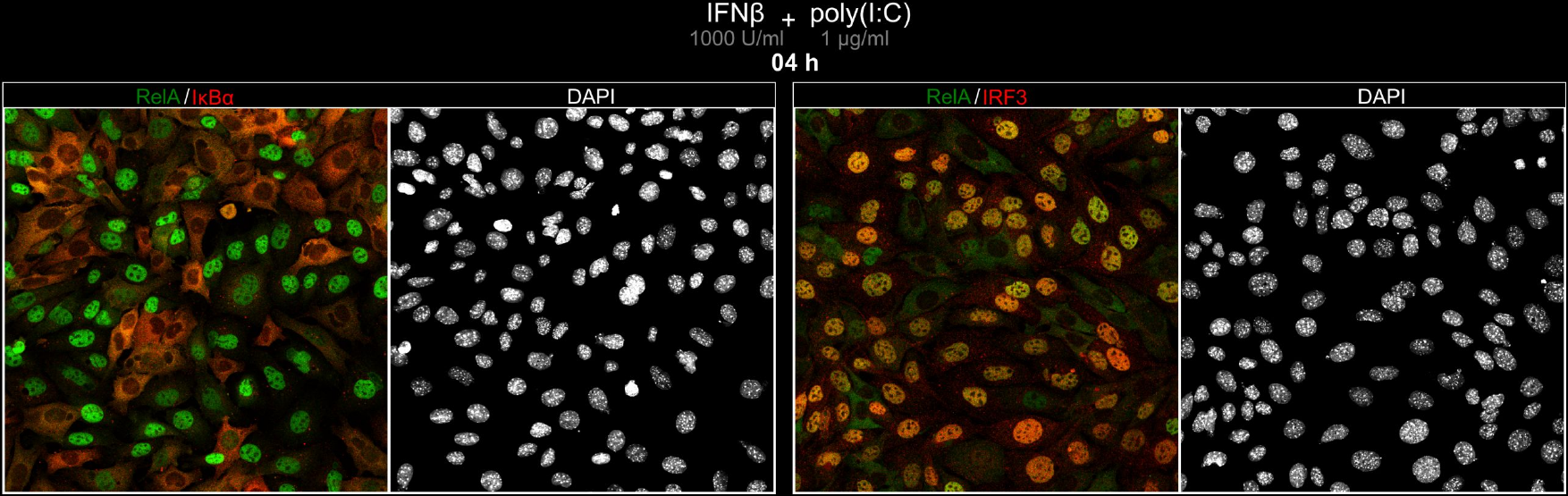

## Slide 4
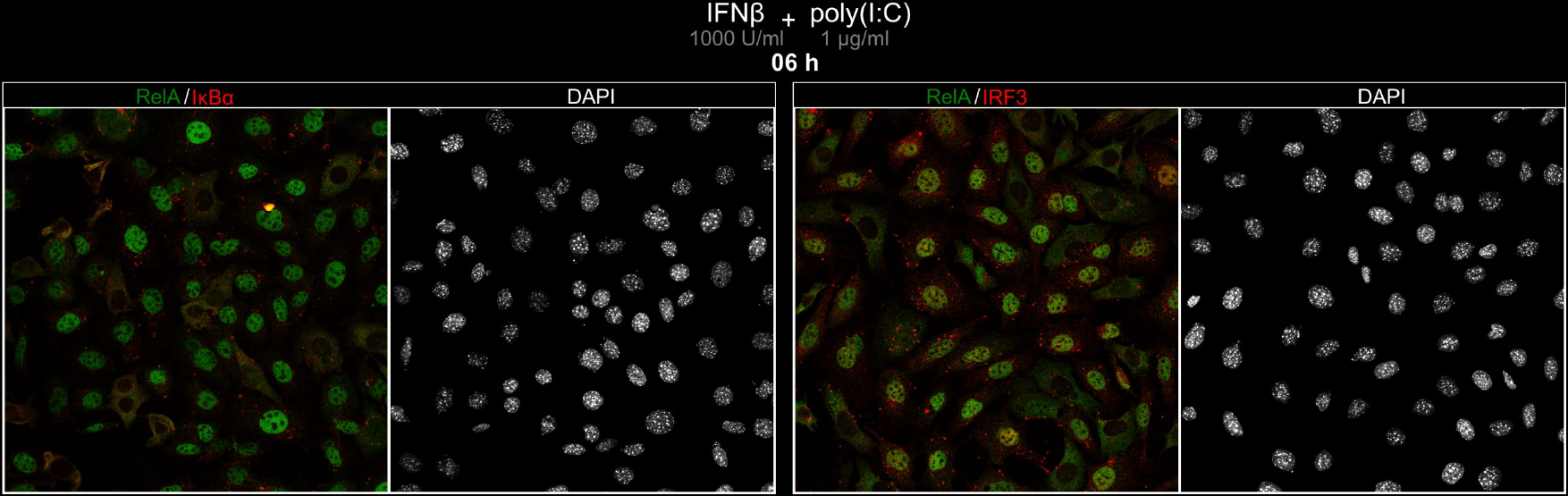

## Slide 5
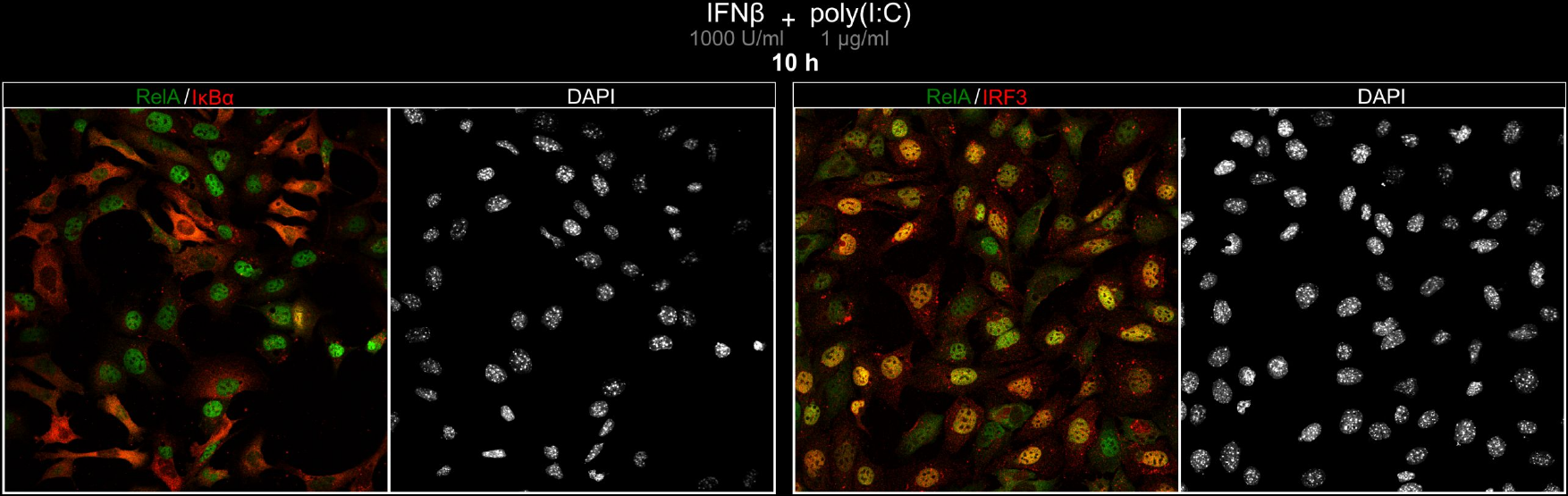

## Slide 6
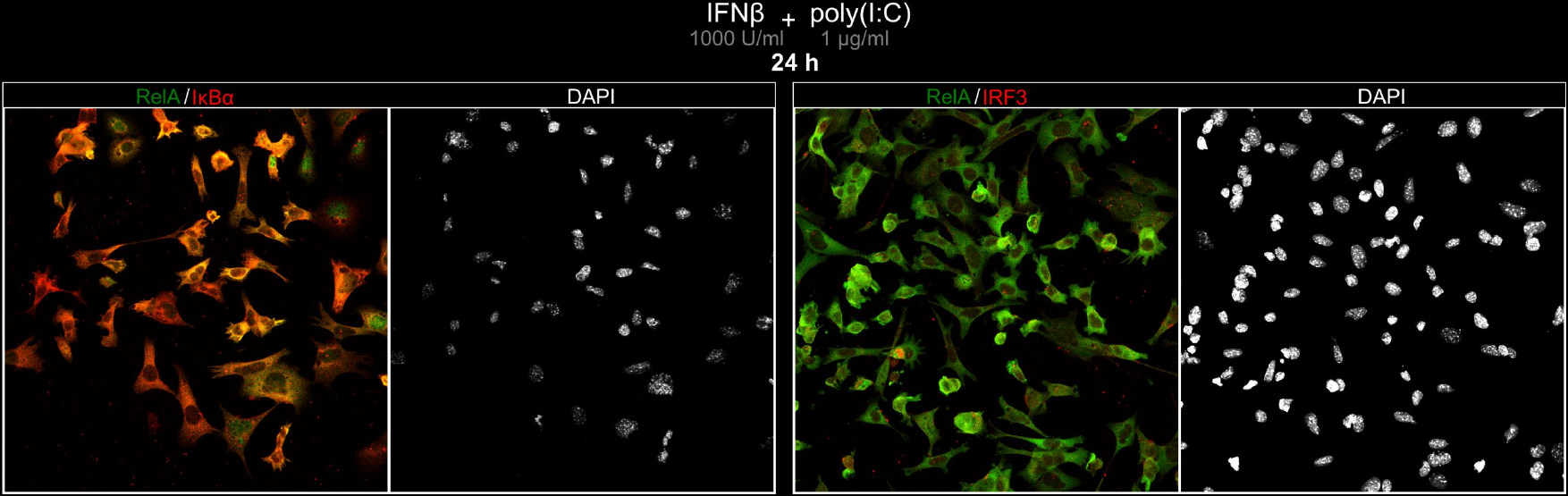

Supplement: Supplementary file 9 — Supplementary Data 6 [file 41467_2017_2640_MOESM9_ESM.ppt]

## Slide 1
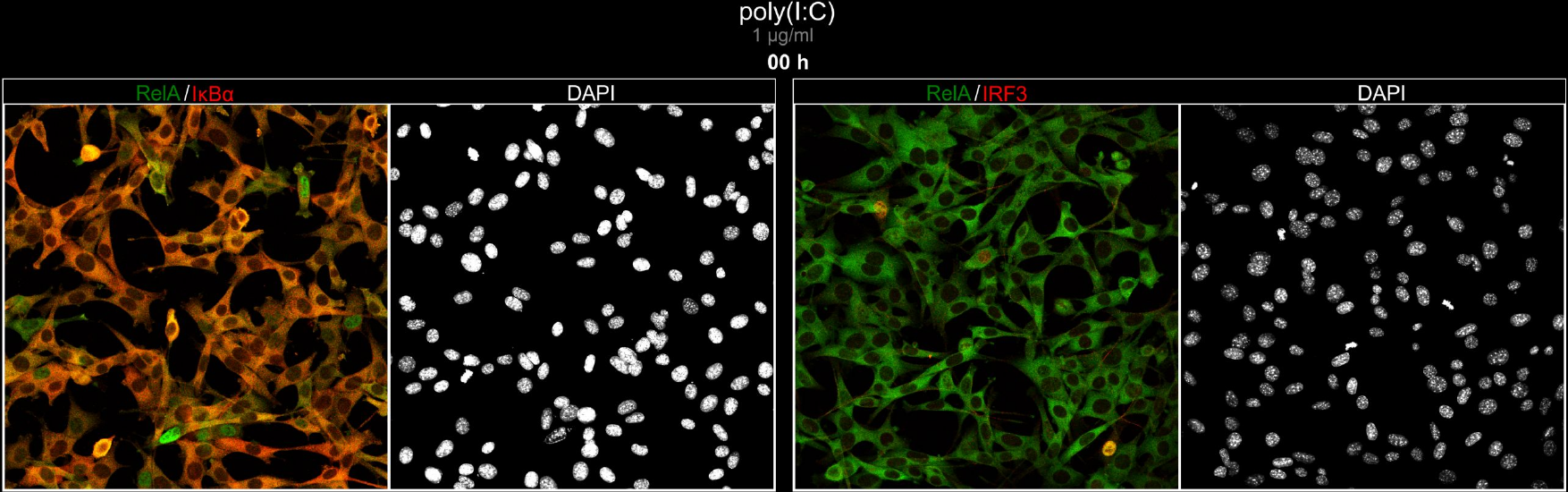

## Slide 2
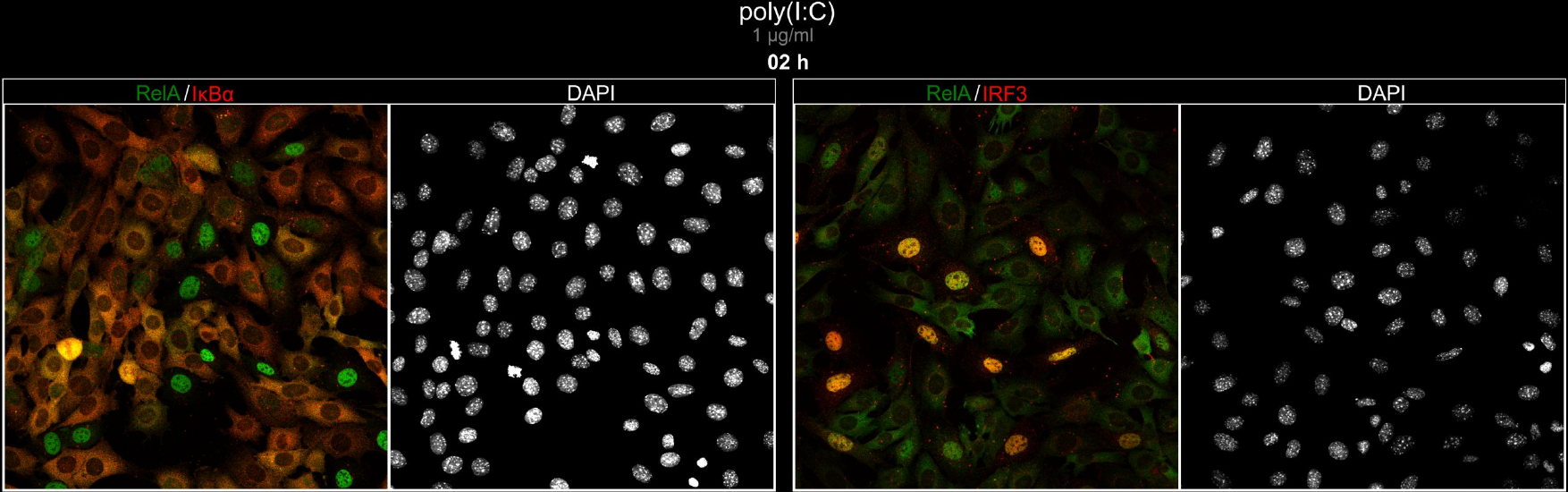

## Slide 3
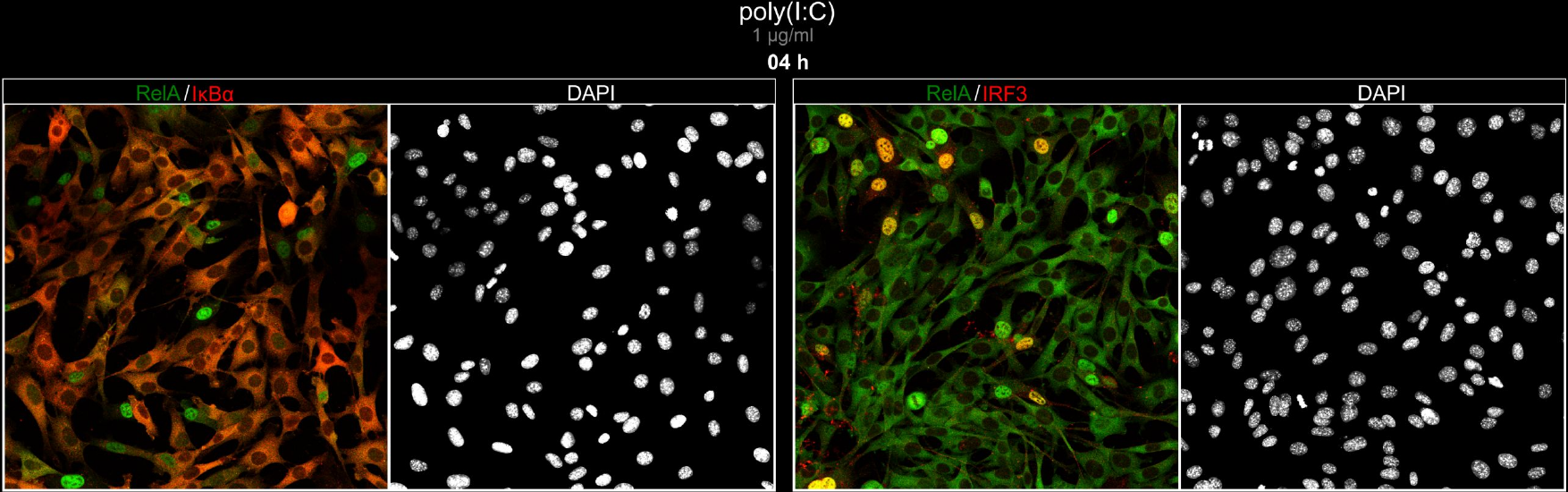

## Slide 4
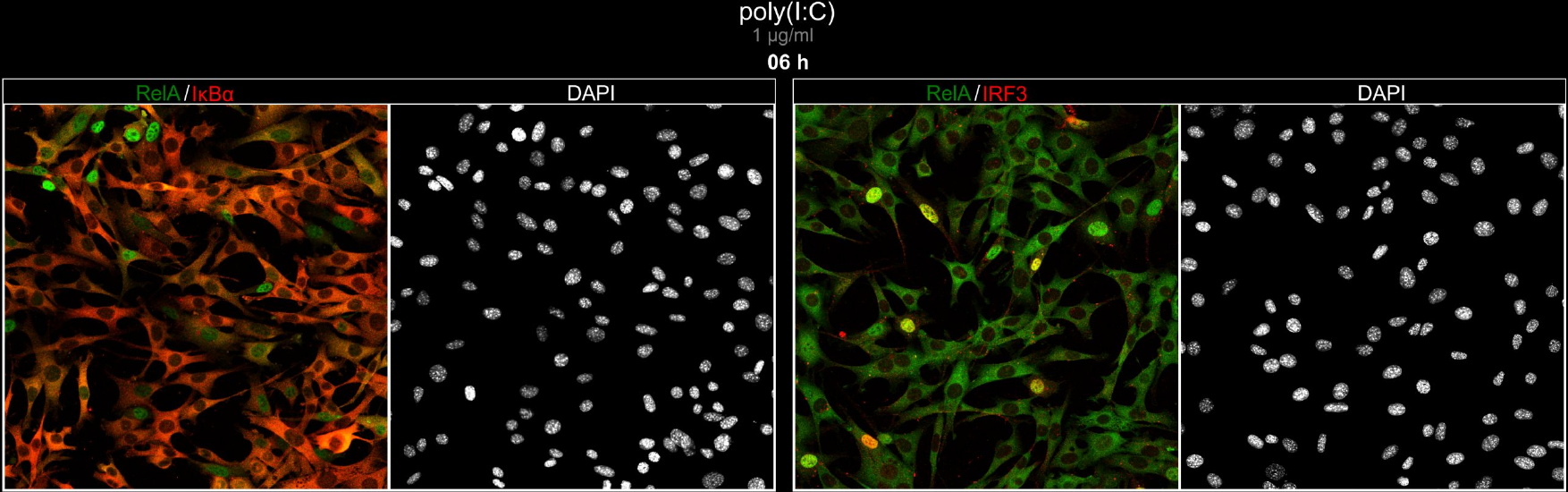

## Slide 5
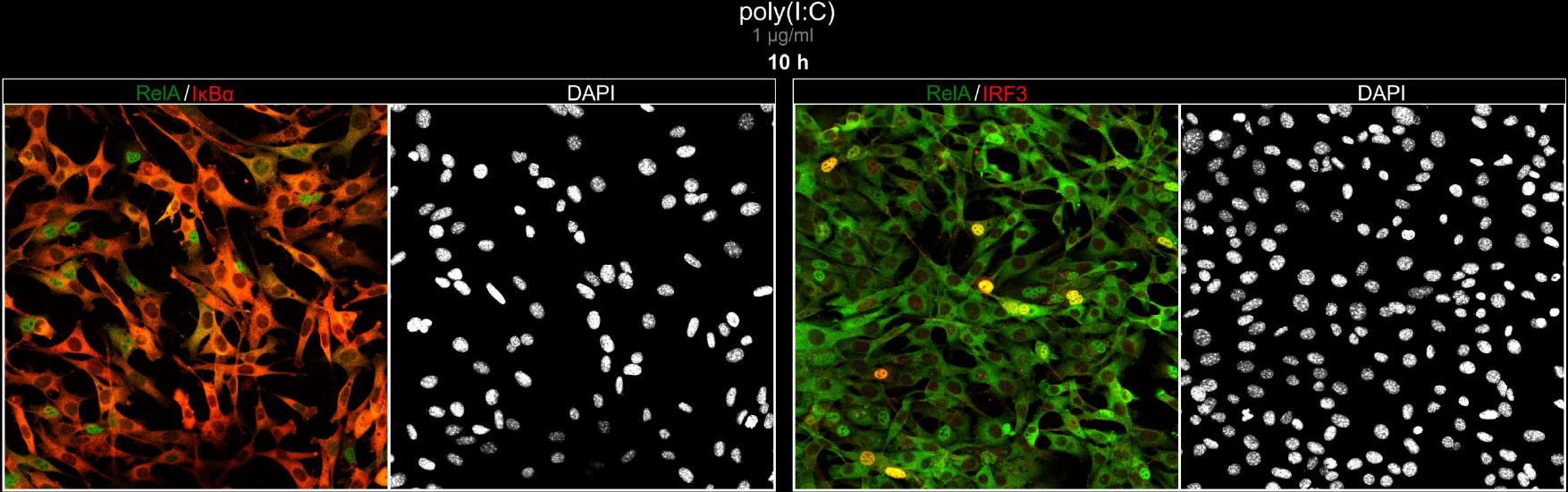

## Slide 6
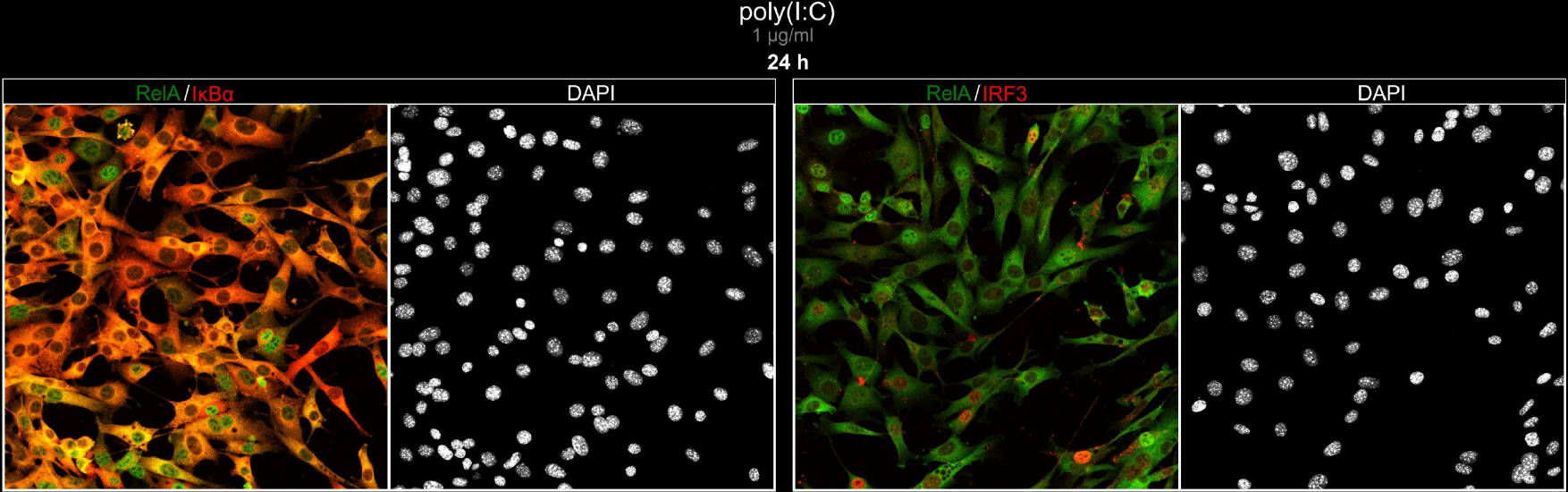

Supplement: Supplementary file 10 — Supplementary Data 7 [file 41467_2017_2640_MOESM10_ESM.ppt]

## Slide 1
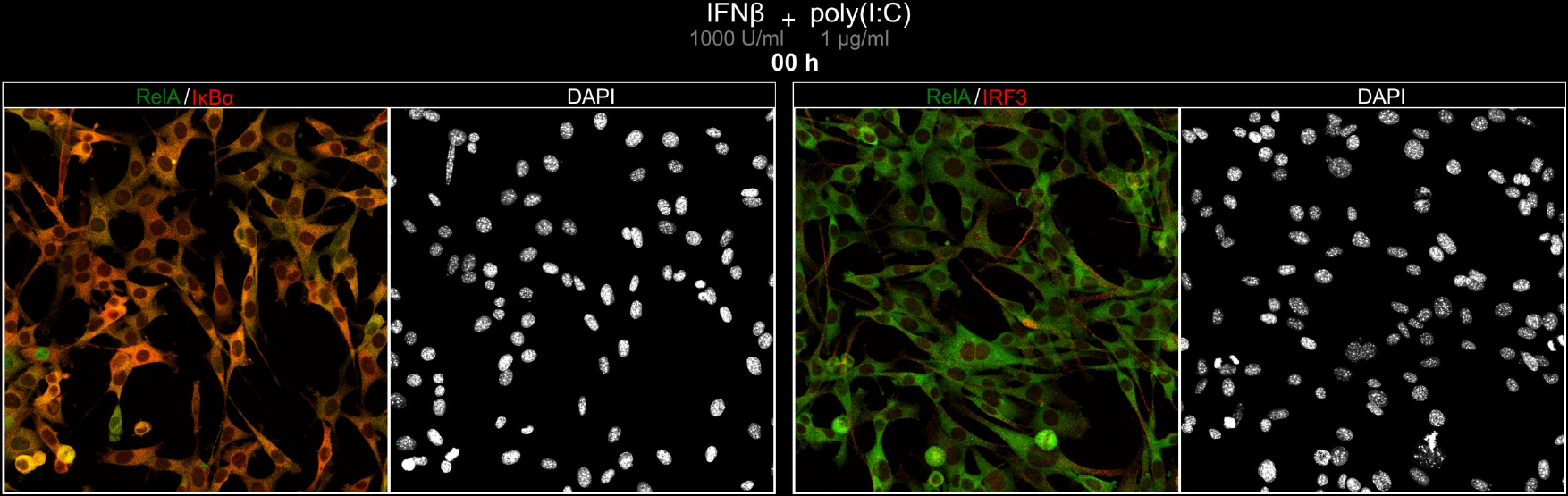

## Slide 2
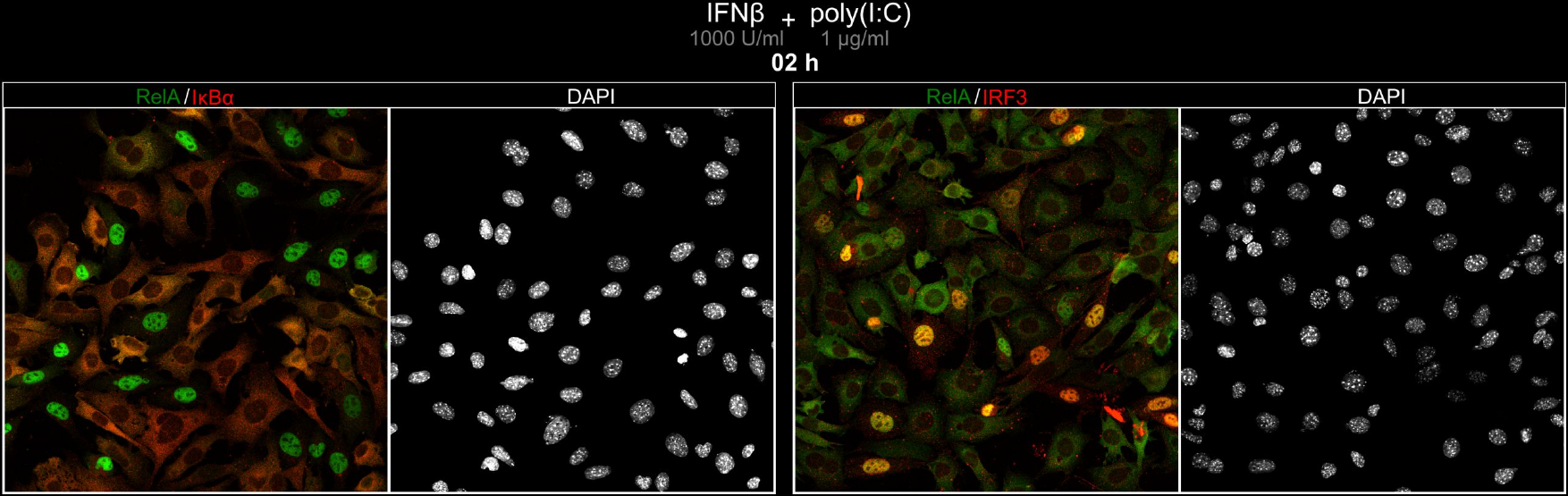

## Slide 3
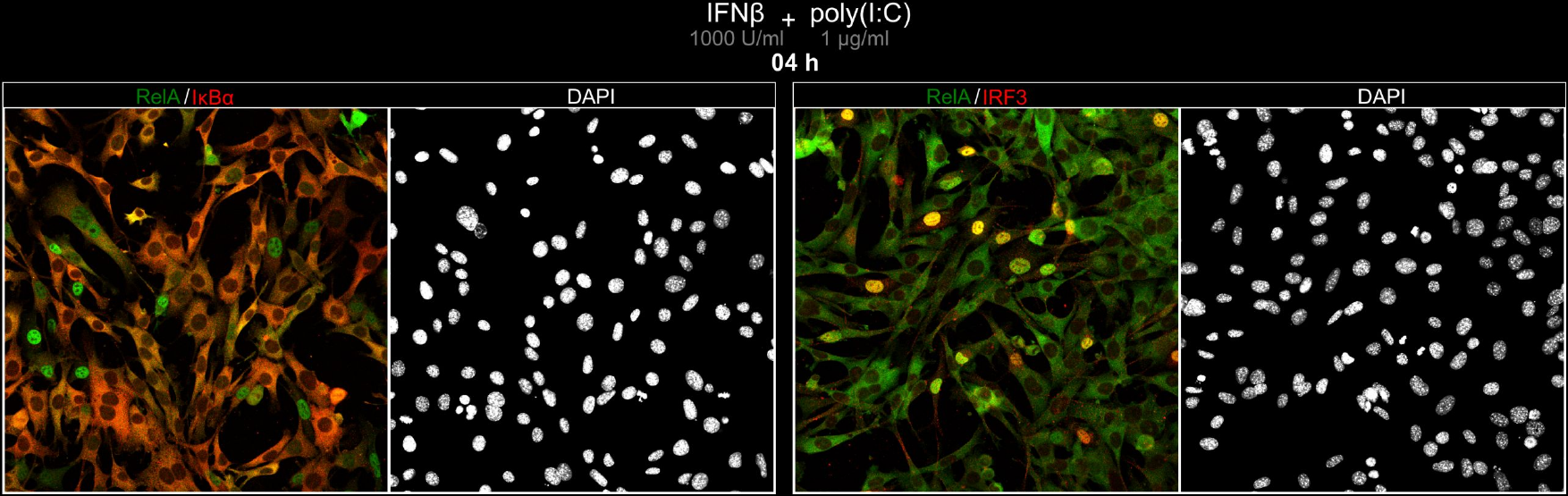

## Slide 4
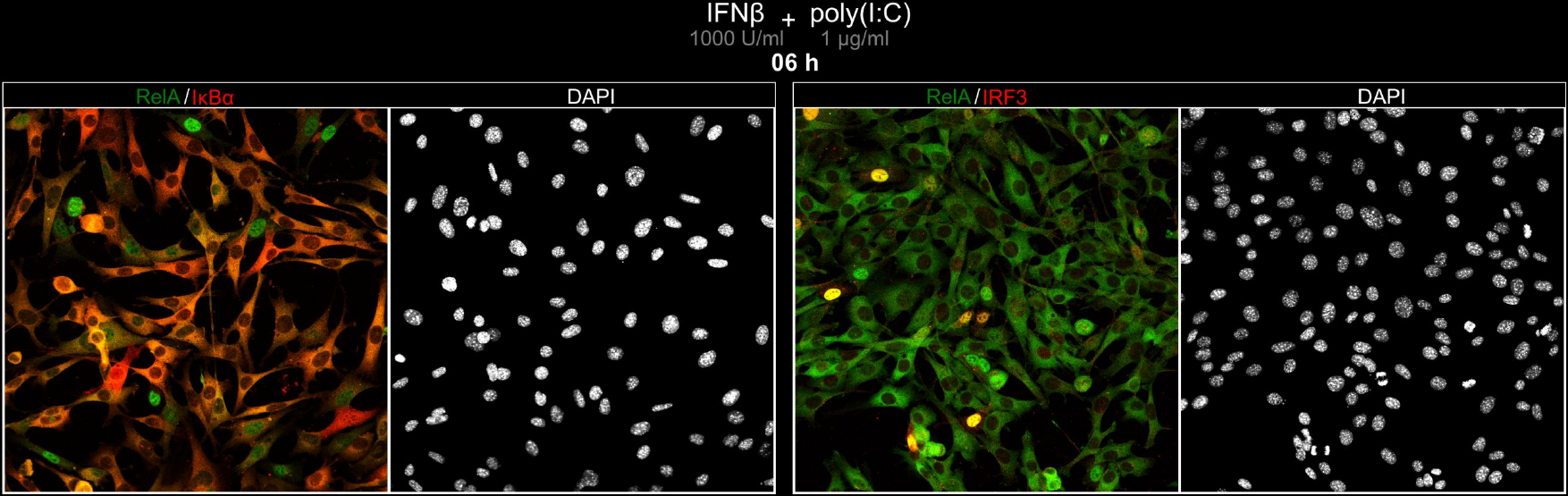

## Slide 5
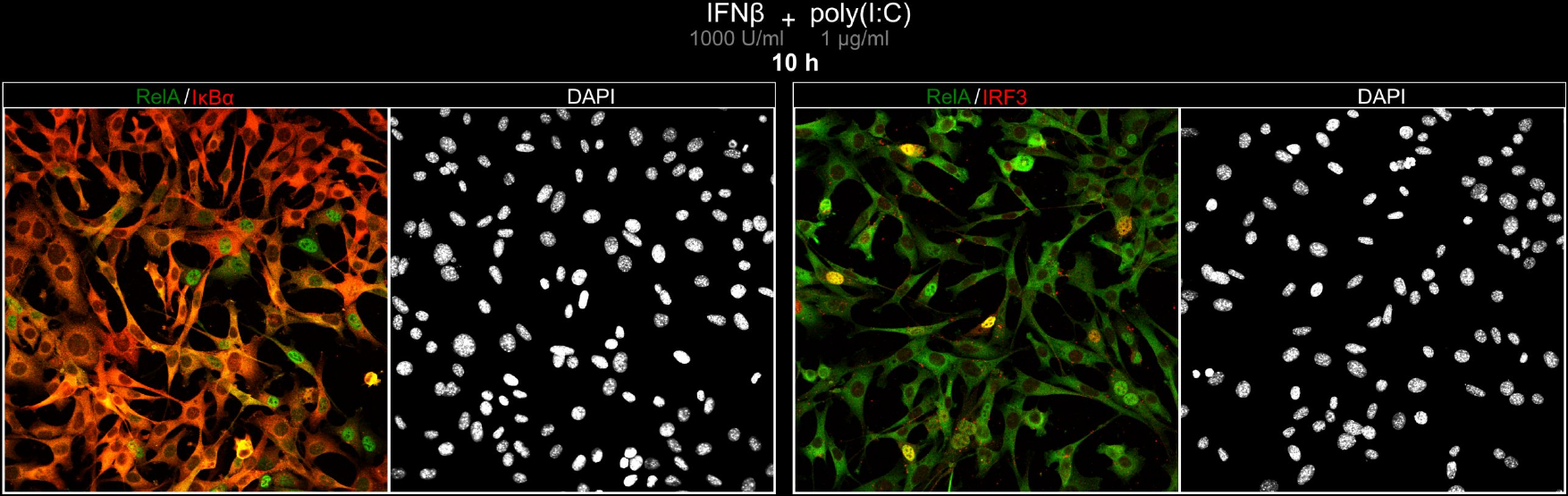

## Slide 6
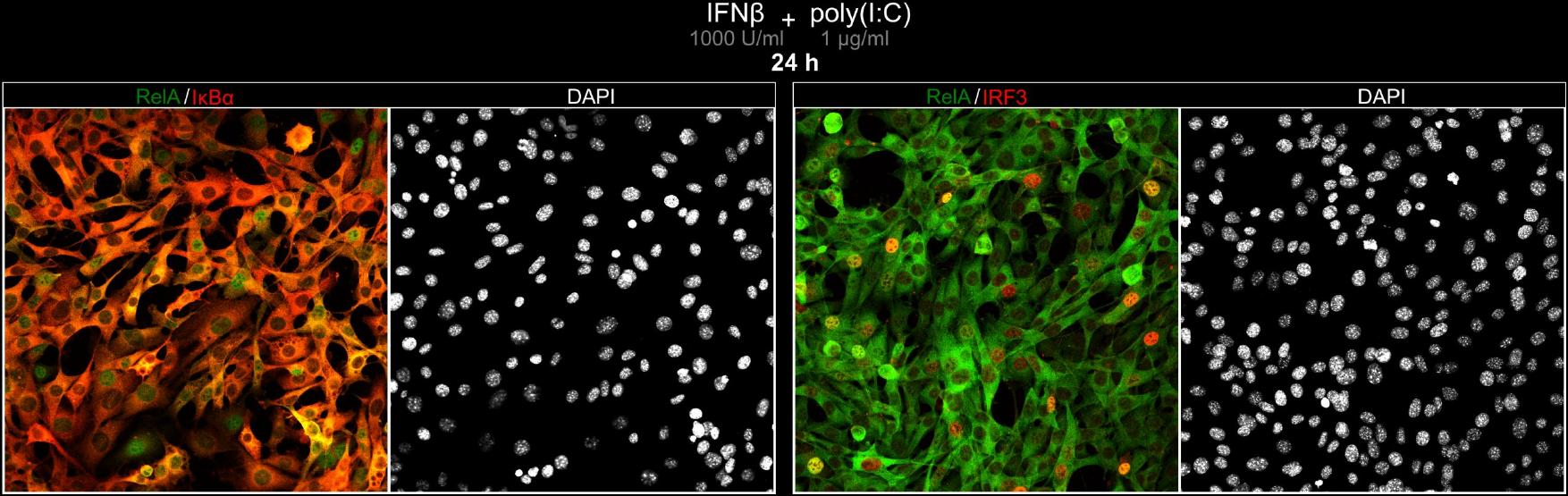

Supplement: Supplementary file 11 — Supplementary Data 8 [file 41467_2017_2640_MOESM11_ESM.ppt]

## Slide 1
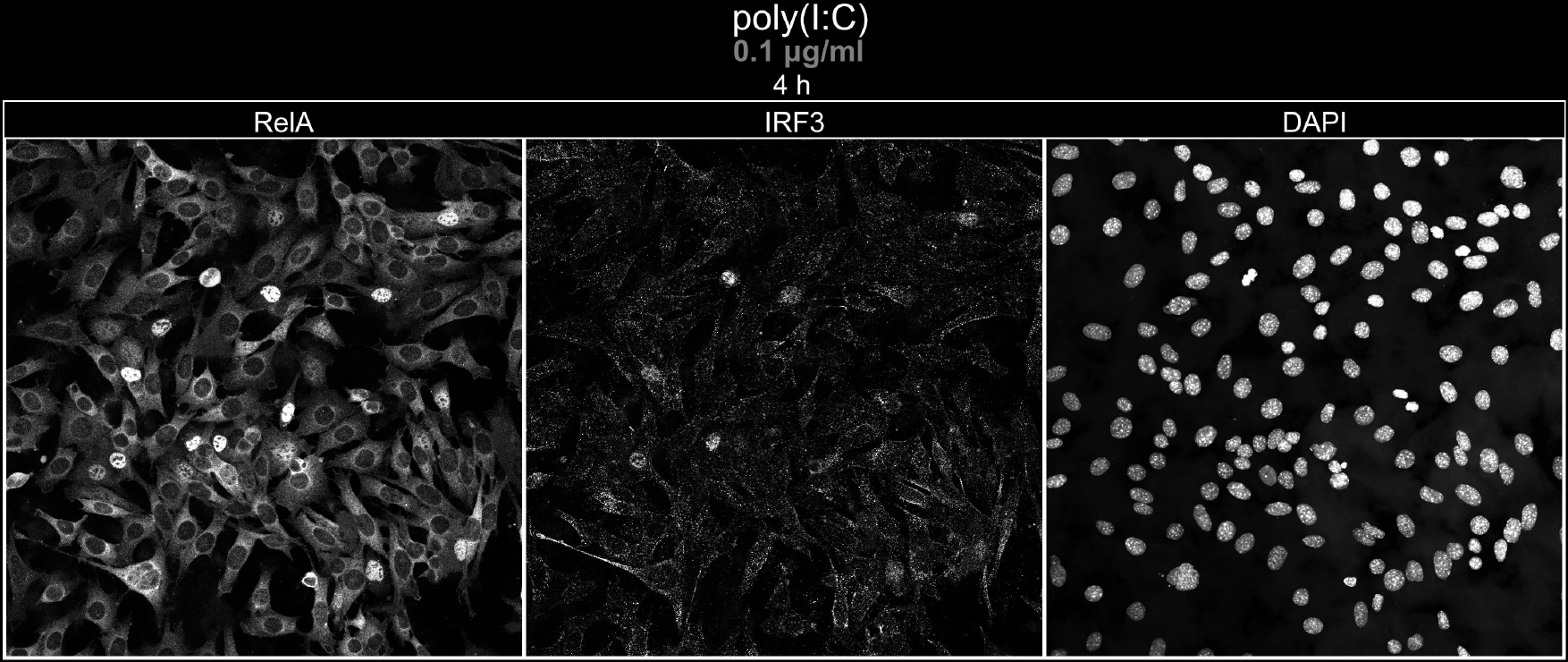

## Slide 2
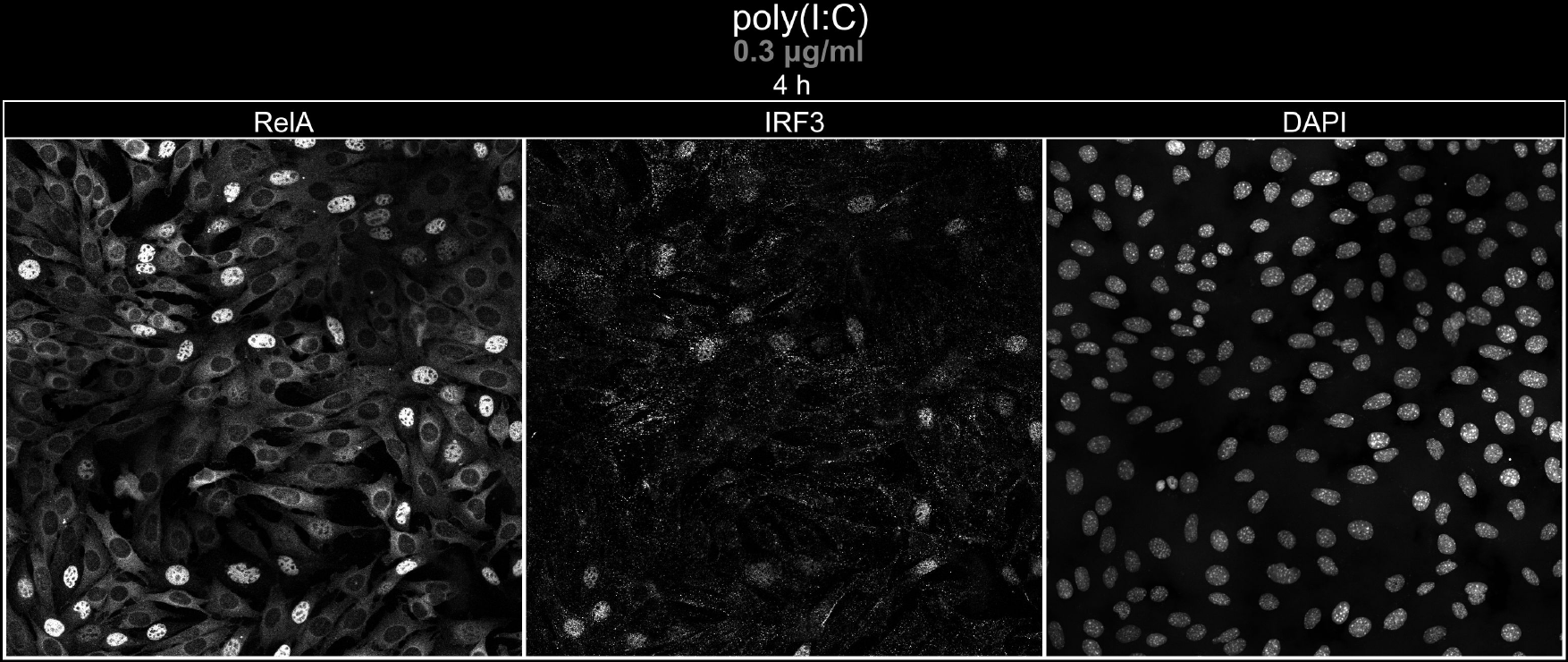

## Slide 3
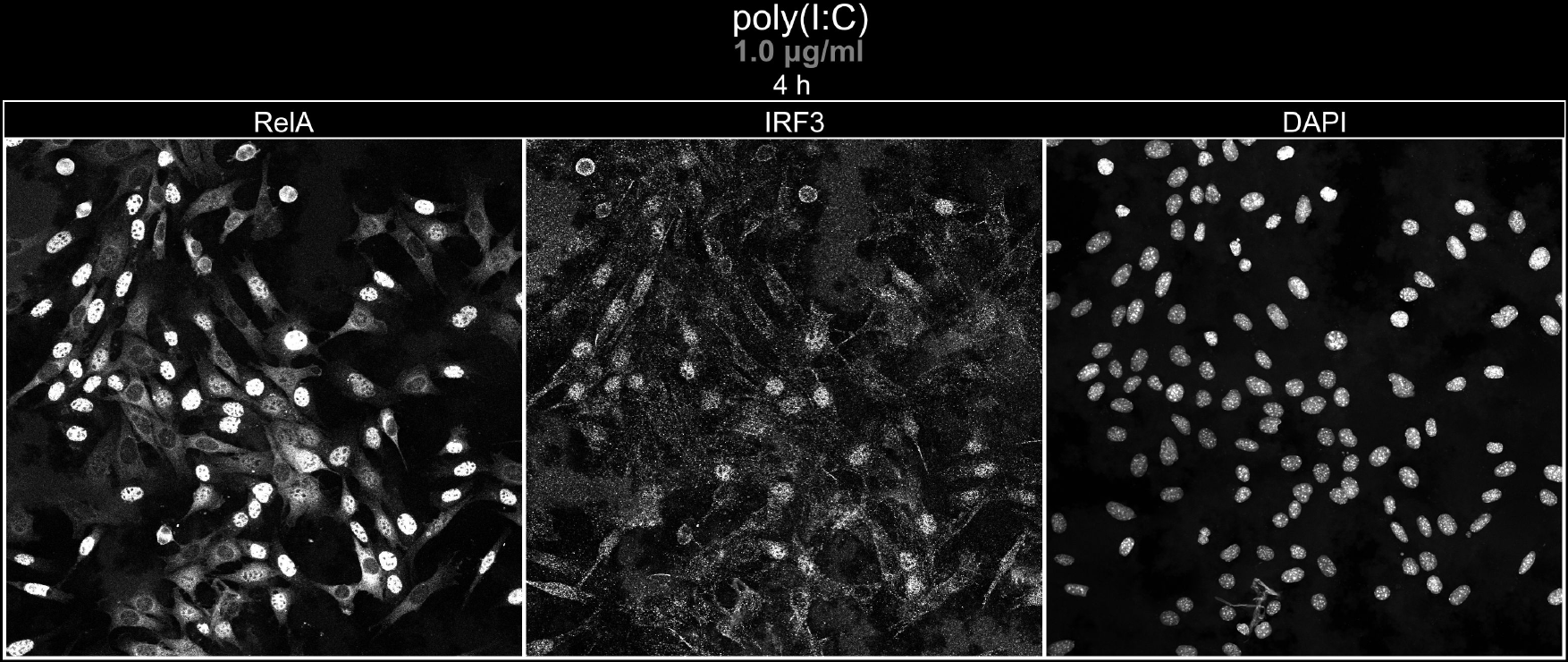

## Slide 4
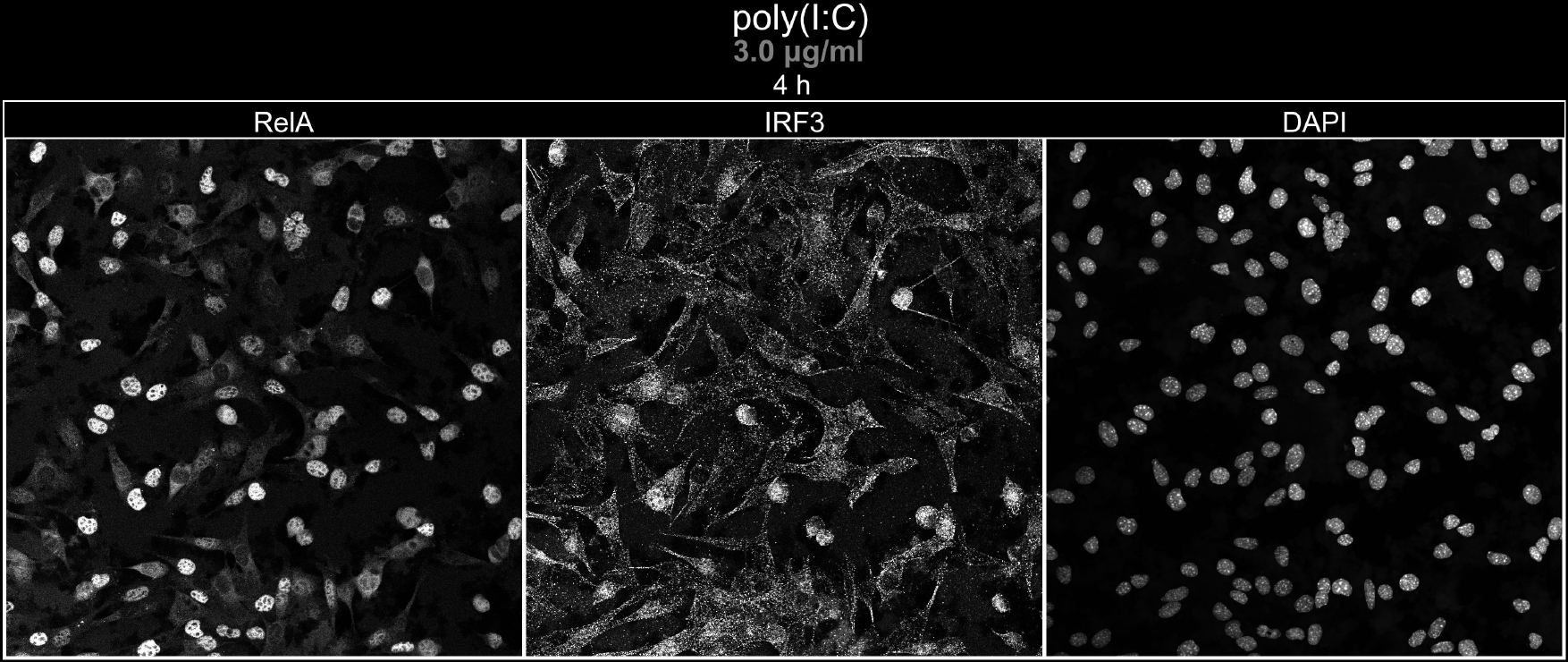

Supplement: Supplementary file 12 — Supplementary Data 9 [file 41467_2017_2640_MOESM12_ESM.ppt]

## Slide 1
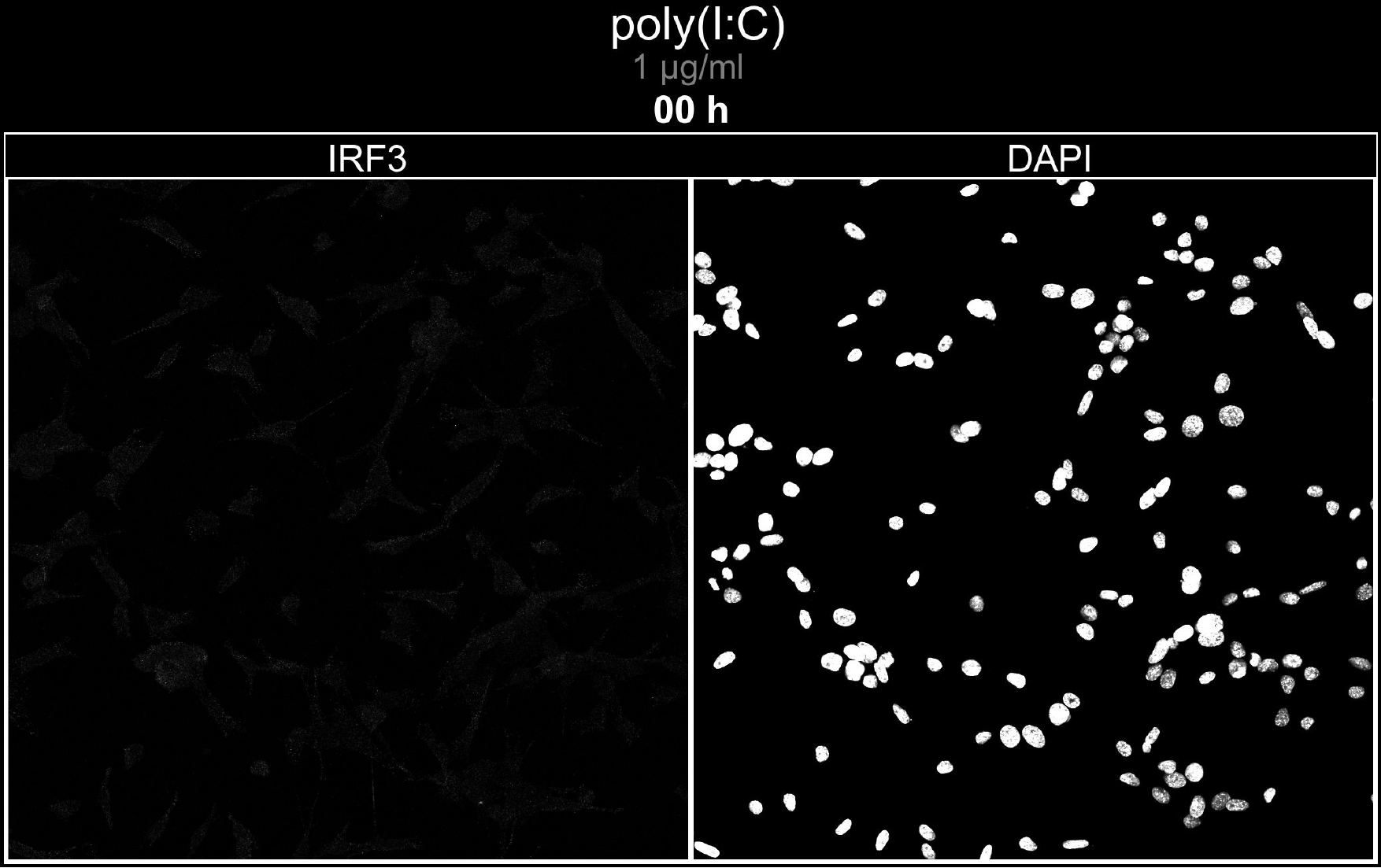

## Slide 2
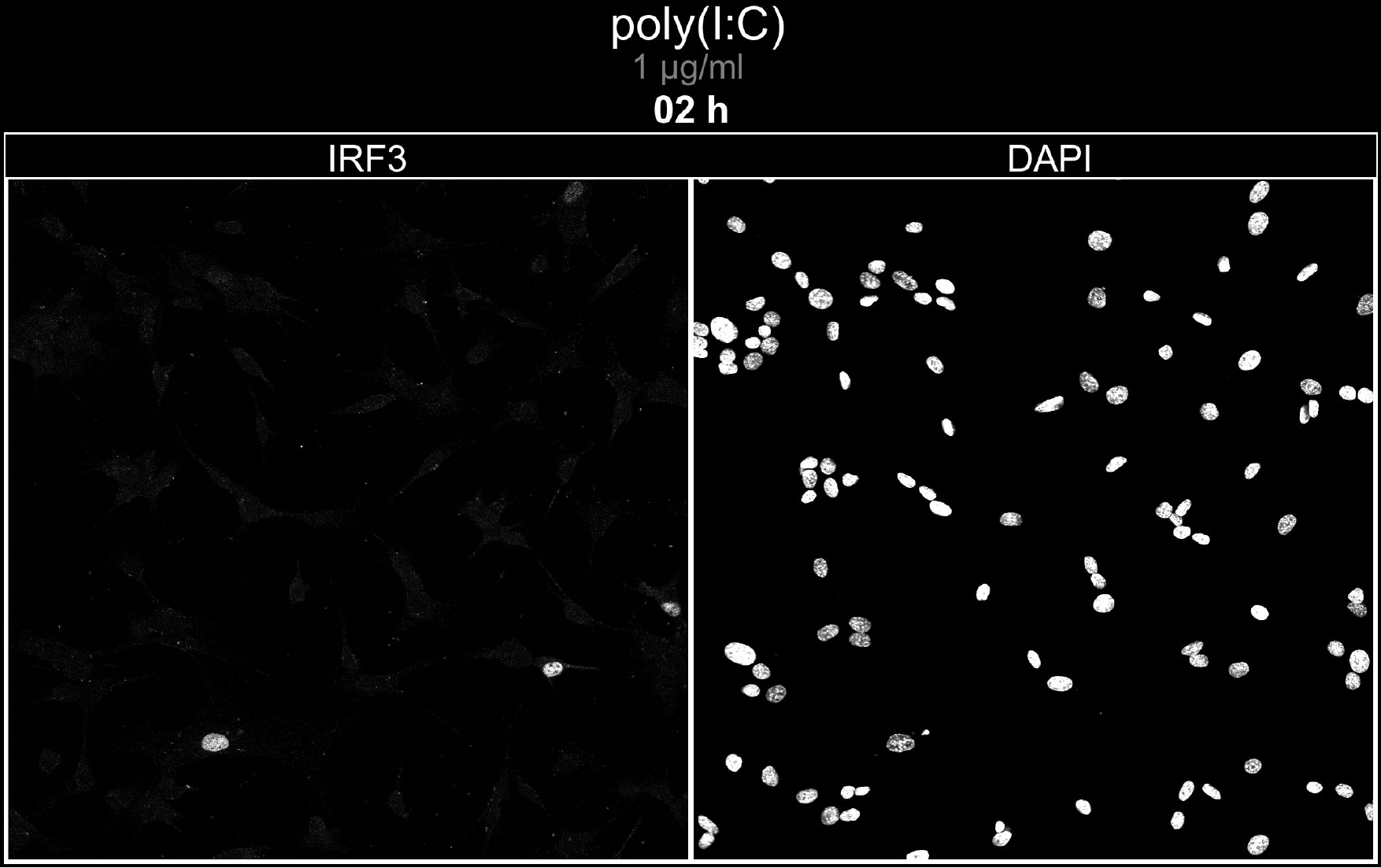

## Slide 3
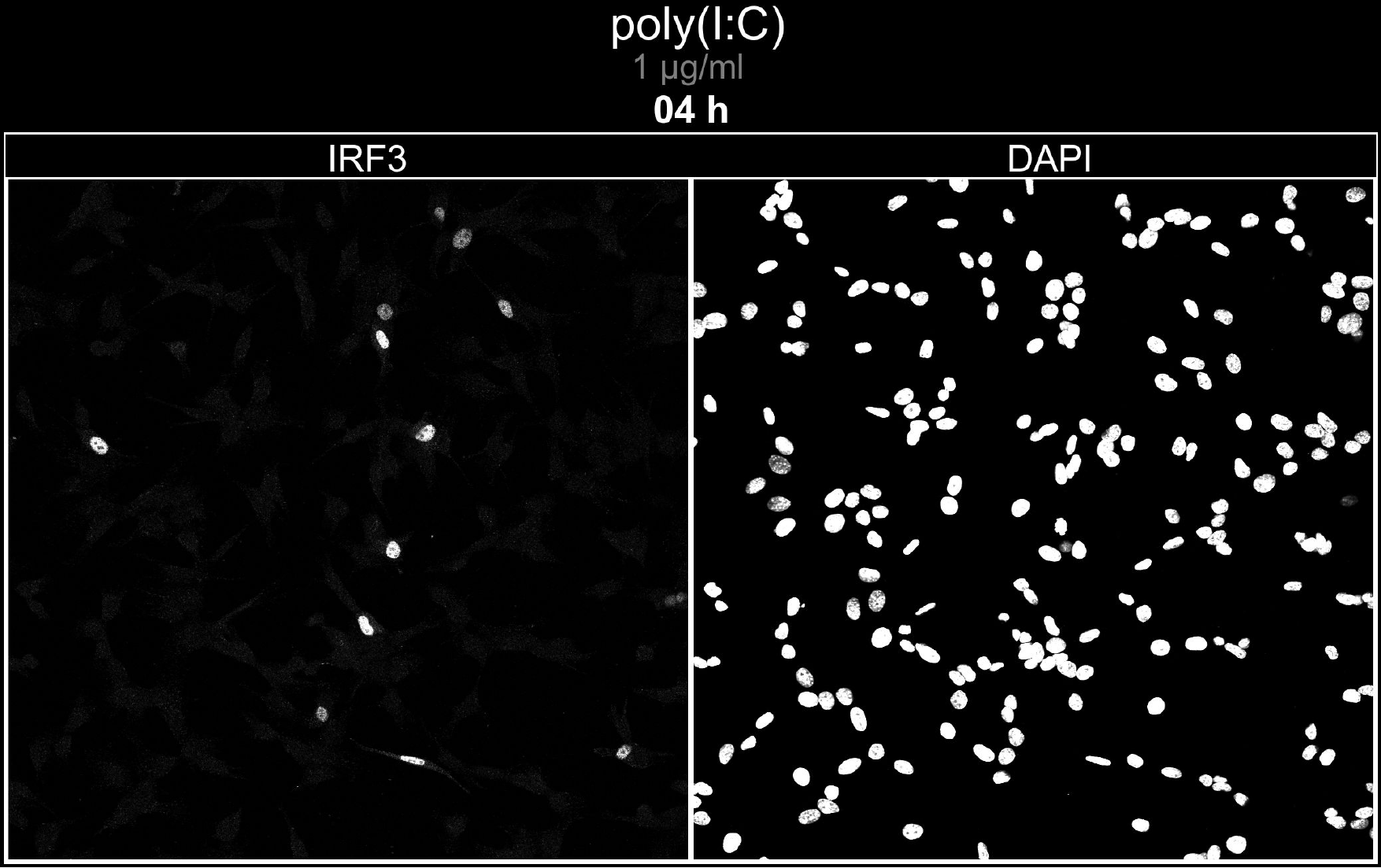

## Slide 4
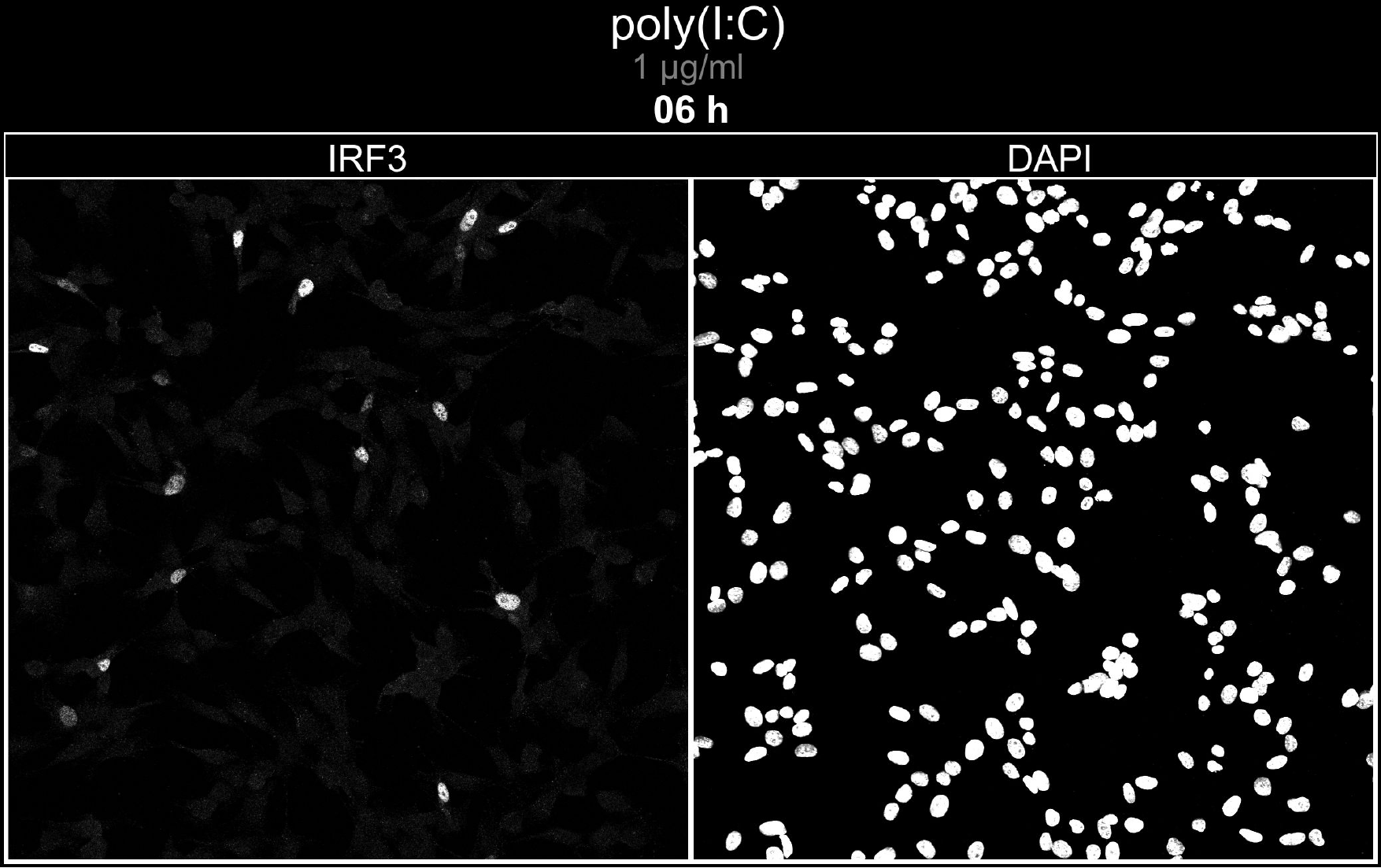

## Slide 5
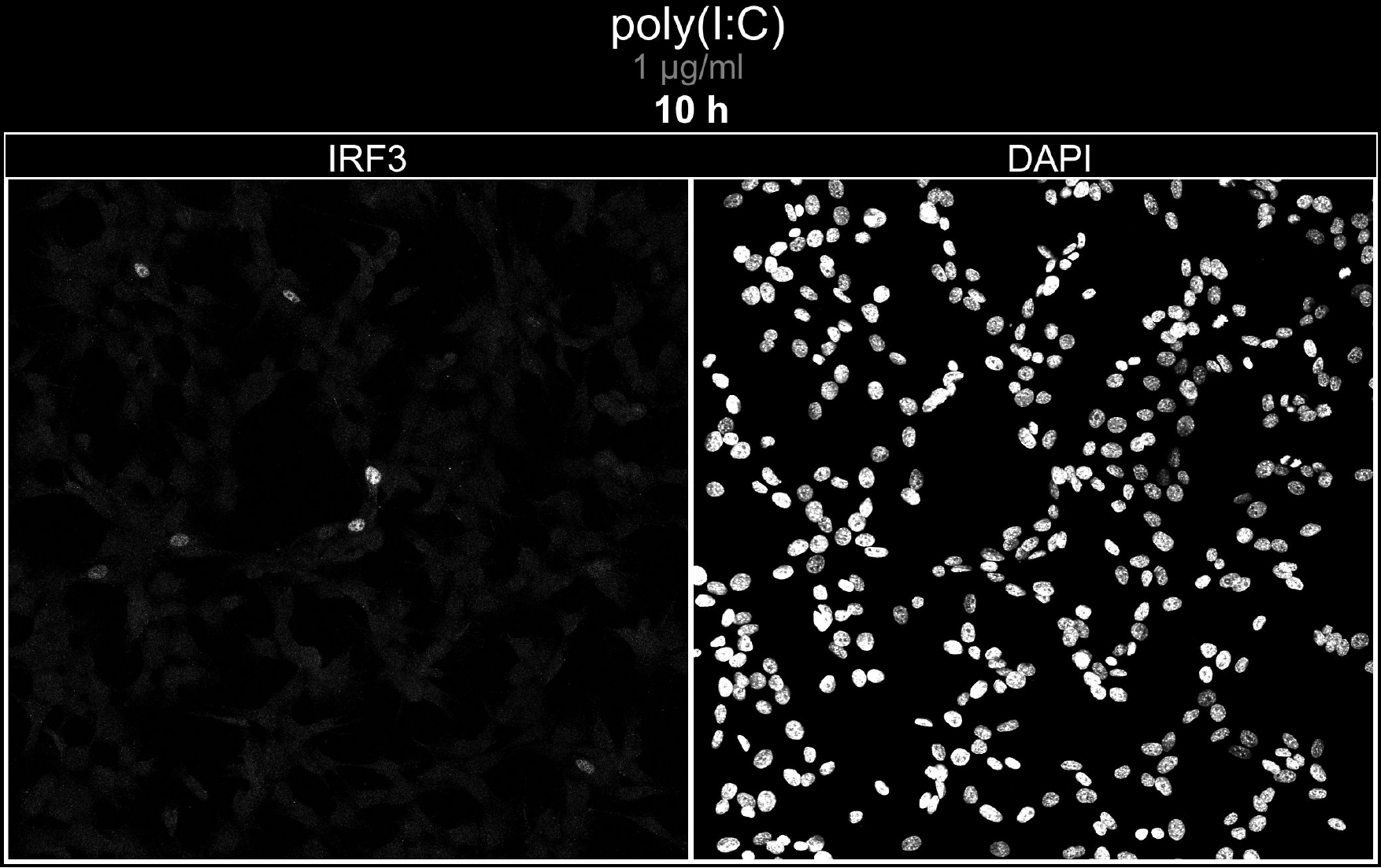

## Slide 6
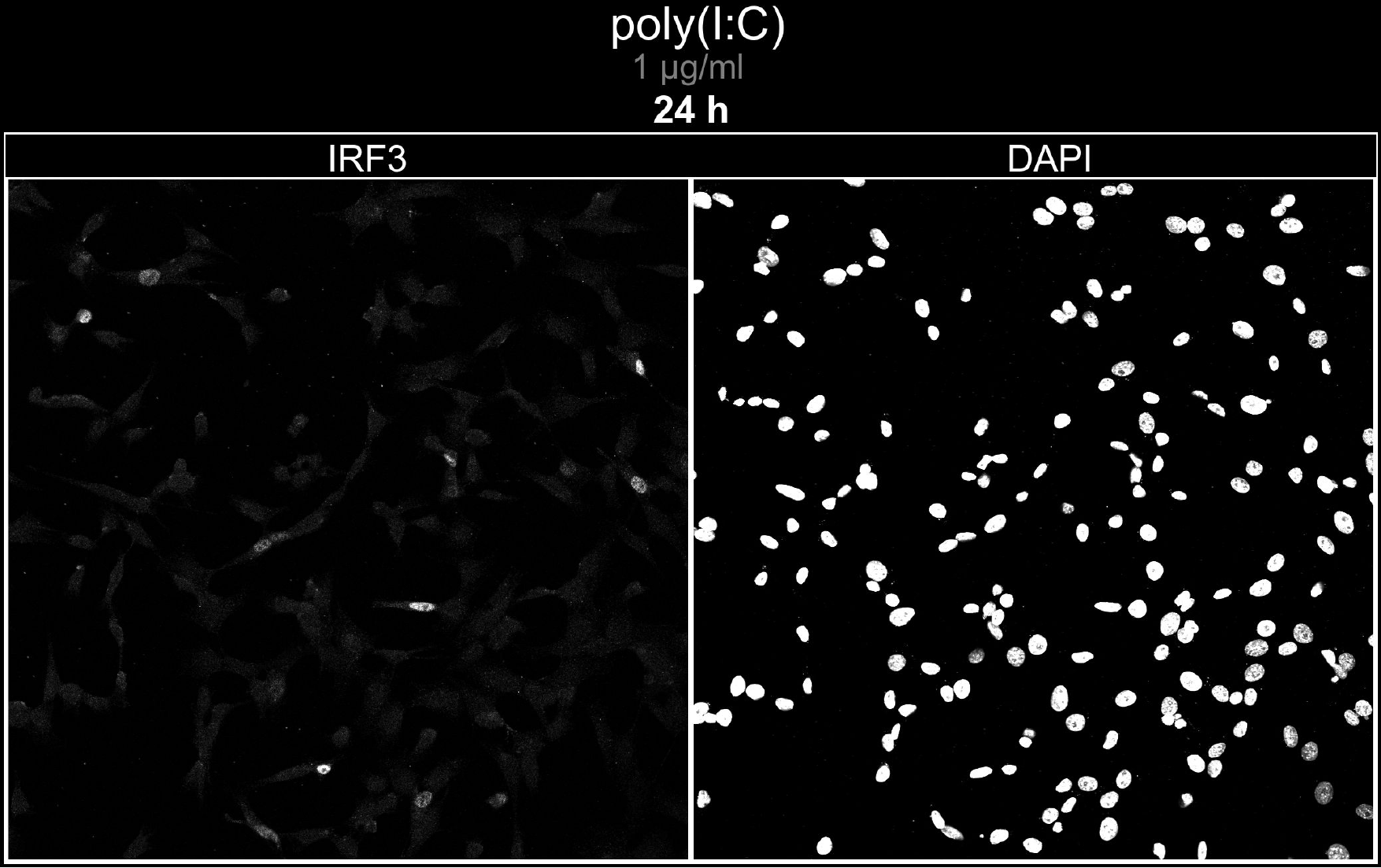

Supplement: Supplementary file 13 — Supplementary Data 10 [file 41467_2017_2640_MOESM13_ESM.ppt]

poly(I:C)

0.01  $\mu$ g 0.05  $\mu$ g 0.1  $\mu$ g 0.2  $\mu$ g 0.4  $\mu$ g

1000 bp

100 bp

1000 bp

500 bp

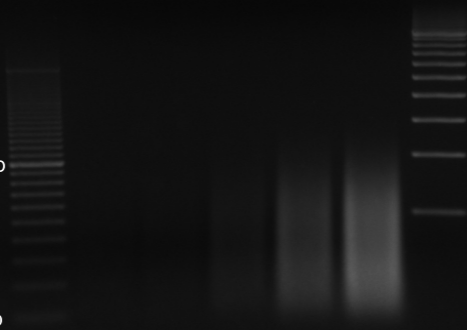

Supplement: Supplementary file 15 — Supplementary Data 12 [file 41467_2017_2640_MOESM15_ESM.pdf]
