# Supplementary material for: Cell fate in antiviral response arises in the crosstalk of IRF, NF-κB and JAK/STAT pathways
Source: Nat Commun. 2018 Feb 5;9:493. doi: 10.1038/s41467-017-02640-8 (PMC5799375; doi:10.1038/s41467-017-02640-8)
Supplement: Supplementary file 14 — Supplementary Data 11 [file 41467_2017_2640_MOESM14_ESM.pptx]

## Slide 1
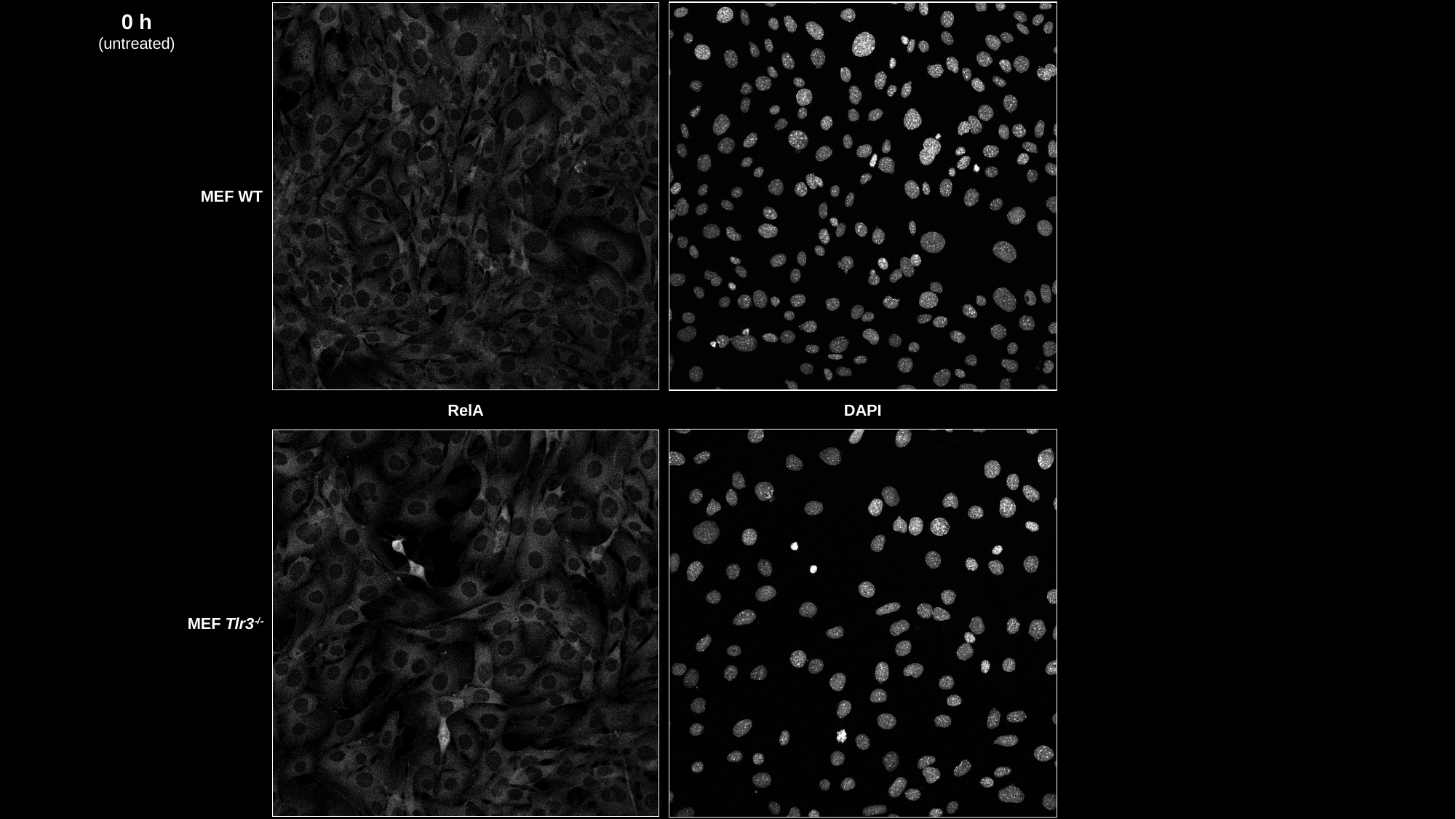

0 h
(untreated)
MEF WT
RelA
DAPI
MEF Tlr3−/−

## Slide 2
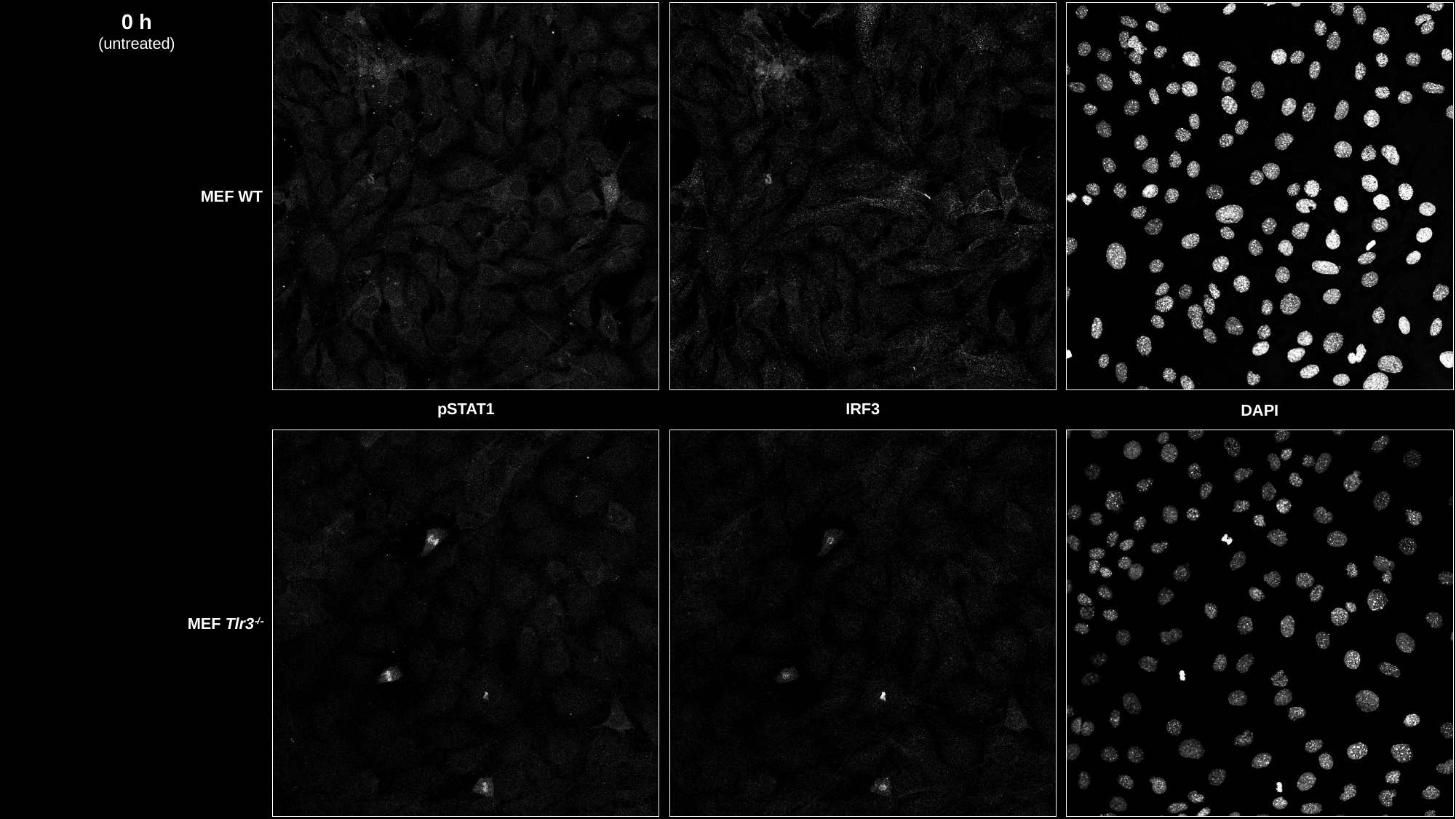

0 h
(untreated)
MEF WT
pSTAT1
IRF3
DAPI
MEF Tlr3−/−

## Slide 3
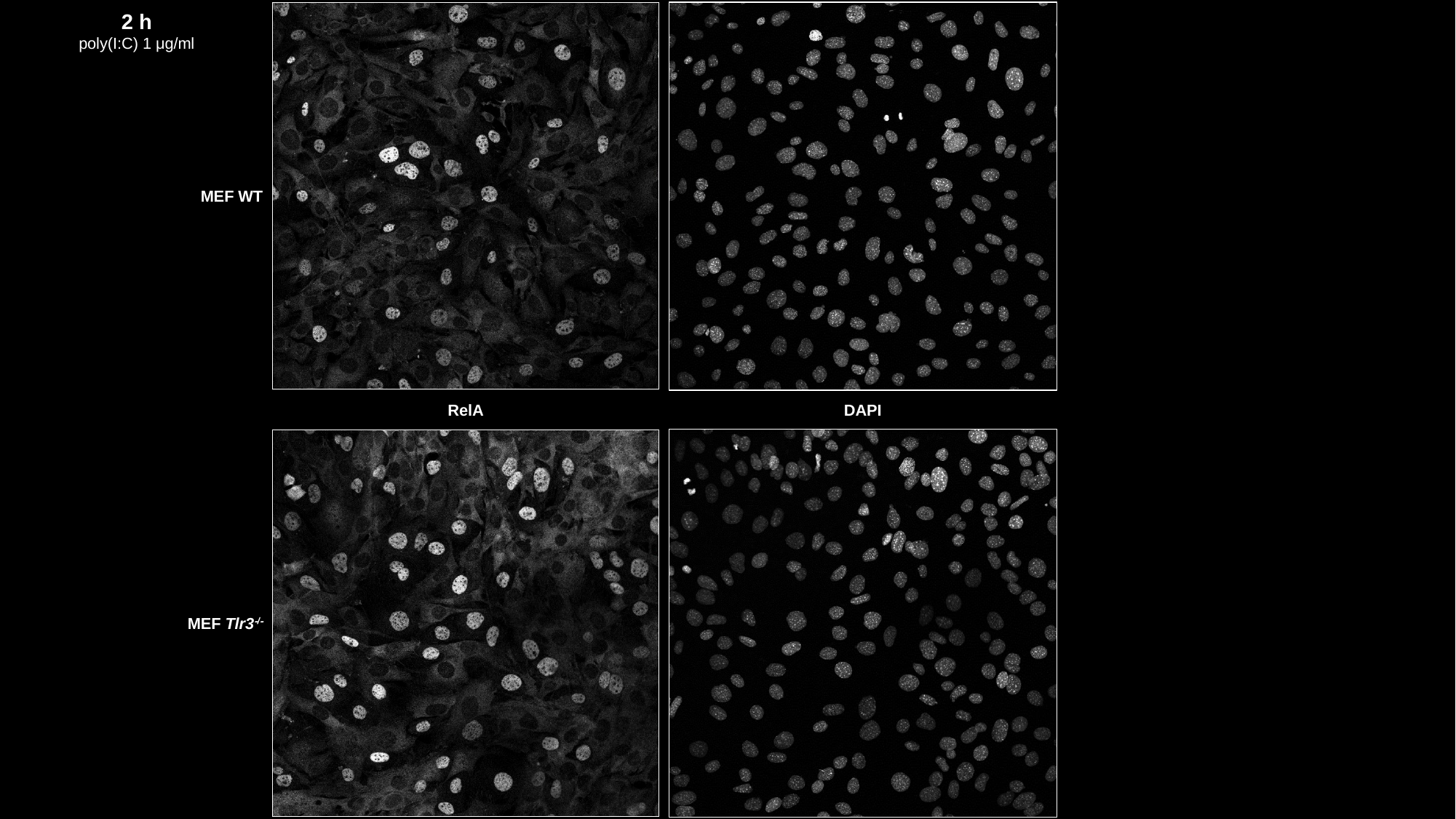

2 h
poly(I:C) 1 μg/ml
MEF WT
RelA
DAPI
MEF Tlr3−/−

## Slide 4
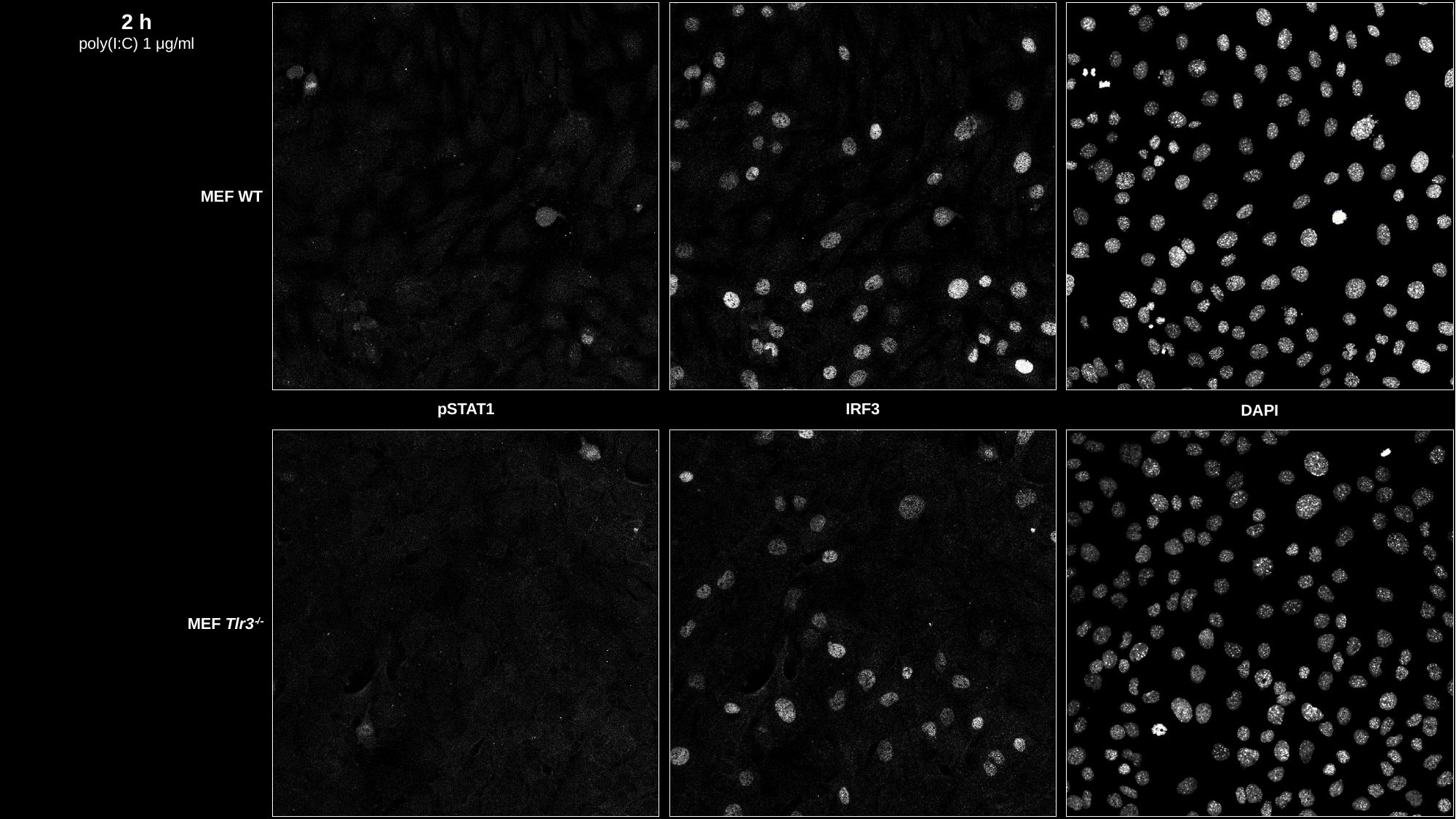

2 h
poly(I:C) 1 μg/ml
MEF WT
pSTAT1
IRF3
DAPI
MEF Tlr3−/−

## Slide 5
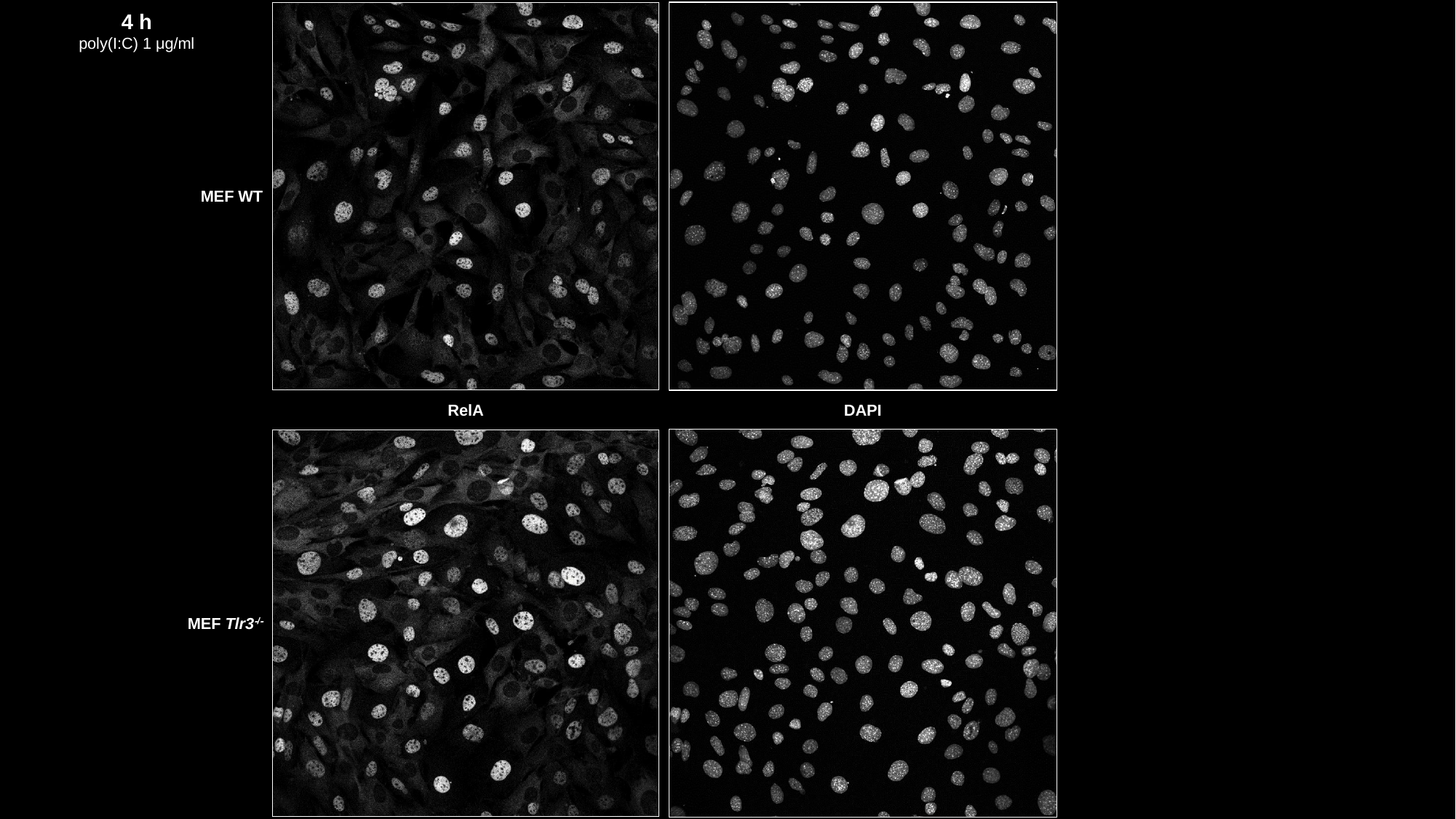

4 h
poly(I:C) 1 μg/ml
MEF WT
RelA
DAPI
MEF Tlr3−/−

## Slide 6
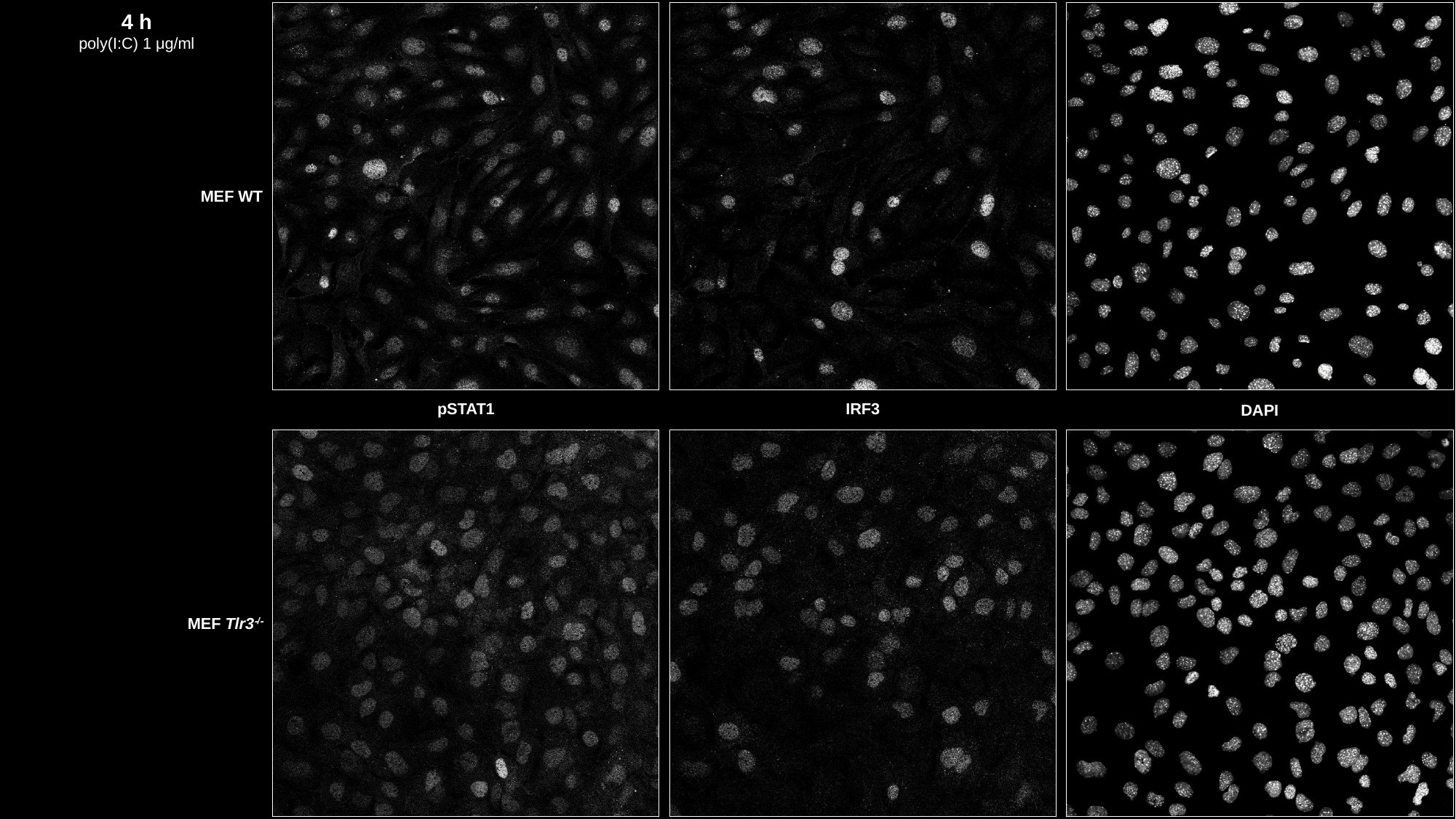

4 h
poly(I:C) 1 μg/ml
MEF WT
pSTAT1
IRF3
DAPI
MEF Tlr3−/−
