# Supplementary material for: Cell fate in antiviral response arises in the crosstalk of IRF, NF-κB and JAK/STAT pathways
Source: Nat Commun. 2018 Feb 5;9:493. doi: 10.1038/s41467-017-02640-8 (PMC5799375; doi:10.1038/s41467-017-02640-8)
Supplement: Supplementary file 16 — Supplementary Data 13 [file 41467_2017_2640_MOESM16_ESM.pdf]

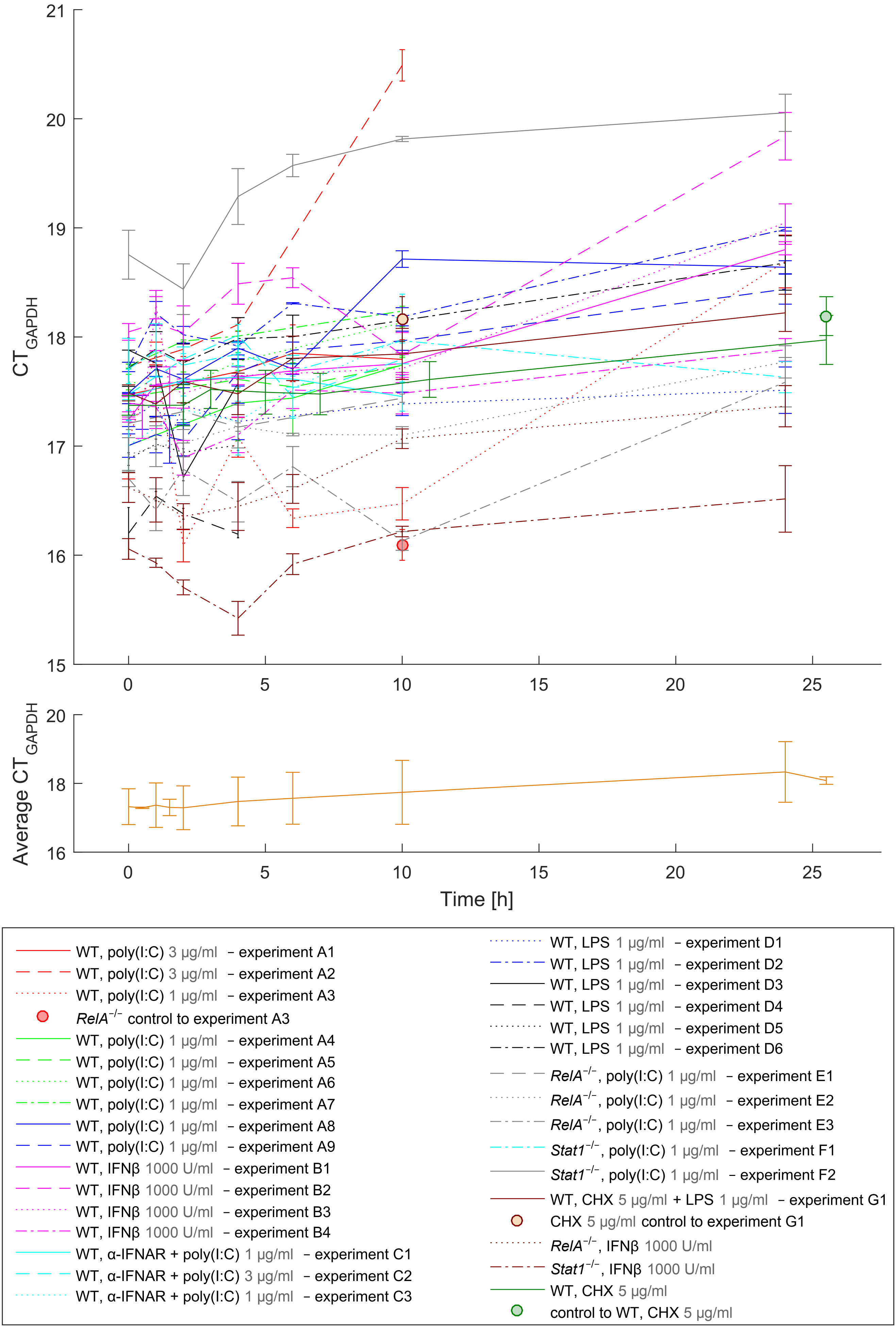

21
20
19
18
17
16
15
CT_{GAPDH}
0
5
10
15
20
25
Average CT_{GAPDH}
Time [h]
WT, poly(I:C) 3 μg/ml – experiment A1
WT, poly(I:C) 3 μg/ml – experiment A2
WT, poly(I:C) 1 μg/ml – experiment A3
RelA$^{-/-}$ control to experiment A3
WT, poly(I:C) 1 μg/ml – experiment A4
WT, poly(I:C) 1 μg/ml – experiment A5
WT, poly(I:C) 1 μg/ml – experiment A6
WT, poly(I:C) 1 μg/ml – experiment A7
WT, poly(I:C) 1 μg/ml – experiment A8
WT, poly(I:C) 1 μg/ml – experiment A9
WT, IFNβ 1000 U/ml – experiment B1
WT, IFNβ 1000 U/ml – experiment B2
WT, IFNβ 1000 U/ml – experiment B3
WT, IFNβ 1000 U/ml – experiment B4
WT, α-IFNAR + poly(I:C) 1 μg/ml – experiment C1
WT, α-IFNAR + poly(I:C) 3 μg/ml – experiment C2
WT, α-IFNAR + poly(I:C) 1 μg/ml – experiment C3
WT, LPS 1 μg/ml – experiment D1
WT, LPS 1 μg/ml – experiment D2
WT, LPS 1 μg/ml – experiment D3
WT, LPS 1 μg/ml – experiment D4
WT, LPS 1 μg/ml – experiment D5
WT, LPS 1 μg/ml – experiment D6
RelA$^{-/-}$, poly(I:C) 1 μg/ml – experiment E1
RelA$^{-/-}$, poly(I:C) 1 μg/ml – experiment E2
RelA$^{-/-}$, poly(I:C) 1 μg/ml – experiment E3
Stat1$^{-/-}$, poly(I:C) 1 μg/ml – experiment F1
Stat1$^{-/-}$, poly(I:C) 1 μg/ml – experiment F2
WT, CHX 5 μg/ml + LPS 1 μg/ml – experiment G1
CHX 5 μg/ml control to experiment G1
RelA$^{-/-}$, IFNβ 1000 U/ml
Stat1$^{-/-}$, IFNβ 1000 U/ml
WT, CHX 5 μg/ml
control to WT, CHX 5 μg/ml
